# Novel biphenyl ester derivatives as tyrosinase inhibitors: Synthesis, crystallographic, spectral analysis and molecular docking studies

**DOI:** 10.1371/journal.pone.0170117

**Published:** 2017-02-27

**Authors:** Huey Chong Kwong, C. S. Chidan Kumar, Siau Hui Mah, Tze Shyang Chia, Ching Kheng Quah, Zi Han Loh, Siddegowda Chandraju, Gin Keat Lim

**Affiliations:** 1 School of Chemical Sciences, Universiti Sains Malaysia, Penang, Malaysia; 2 Department of Engineering Chemistry, Vidya Vikas Institute of Engineering & Technology, Visvesvaraya Technological University, Alanahalli, Mysuru, Karnataka, India; 3 School of Biosciences, Taylor’s University, Lakeside Campus, Subang Jaya, Selangor, Malaysia; 4 X-ray Crystallography Unit, School of Physics, Universiti Sains Malaysia, Penang, Malaysia; 5 Department of Sugar Technology & Chemistry, Sir M.V. PG Center, University of Mysore, Tubinakere, India; Aligarh Muslim University, INDIA

## Abstract

Biphenyl-based compounds are clinically important for the treatments of hypertension and inflammatory, while many more are under development for pharmaceutical uses. In the present study, a series of 2-([1,1'-biphenyl]-4-yl)-2-oxoethyl benzoates, **2**(**a**-**q**), and 2-([1,1'-biphenyl]-4-yl)-2-oxoethyl pyridinecarboxylate, **2**(**r**-**s**) were synthesized by reacting 1-([1,1'-biphenyl]-4-yl)-2-bromoethan-1-one with various carboxylic acids using potassium carbonate in dimethylformamide at ambient temperature. Single-crystal X-ray diffraction studies revealed a more closely packed crystal structure can be produced by introduction of biphenyl moiety. Five of the compounds among the reported series exhibited significant anti-tyrosinase activities, in which **2p**, **2r** and **2s** displayed good inhibitions which are comparable to standard inhibitor kojic acid at concentrations of 100 and 250 μg/mL. The inhibitory effects of these active compounds were further confirmed by computational molecular docking studies and the results revealed the primary binding site is active-site entrance instead of inner copper binding site which acted as the secondary binding site.

## Introduction

Biphenyl are two adjoined benzene rings that attached through their 1,1'-positions. It appeared as a white crystal with pleasant odor, which served as an important structure analog in various synthesis. The most widely used biphenyl derivatives is polychlorinated biphenyls (PCBs) in electrical and chemical industries as dielectric fluids and heat transfer agents [1]. Biphenyl moiety also served as central building block for basic liquid crystal [2] and fluorescent layers in OLEDs [3]. As for pharmaceutical uses, to date, there are two simple biphenyl derivatives which have been applied in clinical usage to treat hypertension [4] and inflammatory [5]; and many more are in development as potential anti-cholinesterase [6], anti-diabetic [7], anti-tumor [8], anti-cancer [9] and anti-leukemia agent [10], and as a potential therapeutics for cardiovascular disease [11] and osteoporosis [12]. The anti-tyrosinase activities of biphenyl-based compounds were also reported [13–15]. Tyrosinase (EC 1.14.18.1) is a multi-functional copper-containing enzyme that plays a crucial role in melanin biosynthesis and melanin contributes to skin pigmentation. Therefore, tyrosinase inhibitors were useful in the treatment of dermatological disorder that associated with melanin hyperpigmentation, in cosmetic for whitening and in depigmentation after sunburn [16]. The biological activities of biphenyl derivatives and their use as tyrosinase inhibitor inspired us to work on the synthesis of a series of new biphenyl esters andto evaluate their anti-tyrosinase activites. In the current project, we focused on the design and synthesis of new anti-tyrosinase agents with biphenyl-based structure to reach more active analogs towards inhibition of tyrosinase. Besides, we hope the new analogs to render minimum side effects. We also investigated in-silico binding mode of the proposed ligands into tyrosinase enzyme in comparison with kojic acid as reference drug by docking procedure. In fact, it revealed biphenyl-based derivatives have similar pharmacophoric pattern like kojic acid and are able to bind at the active-site entrance.

## Material and methods

All reagents and solvents were obtained commercially from Sigma Aldrich Corporation with high purity. Melting points were determined on Stuart (UK) SMP10 apparatus. ^1^H and ^13^C nuclear magnetic resonance (NMR) spectra were recorded in CDCl_3_ at 500 MHz and 125 MHz, respectively, using Bruker Avance III 500 spectrometer. Fourier transform infrared spectroscopy (FTIR) spectra were recorded on Perkin Elmer Frontier FTIR spectrometer equipped with attenuated total reflection (ATR). The X-ray diffraction analysis were performed using Bruker APEX II DUO CCD diffractometer, employing MoKα radiation (λ = 0.71073 Å) with *φ* and *ω* scans. Data reduction and absorption correction were performed using SAINT and SADABS program [17]. All X-ray structures were solved by using direct methods and refined by using full-matrix least-squares techniques on *F*^*2*^ through SHELXTL software package [18]. The C-bound H atoms were calculated geometrically with isotropic displacement parameters set to 1.2times the equivalent isotropic *U* value of the parent carbon atoms. N-bound H atoms are located from difference Fourier map and refined freely [N—H = 0.87 (3)—0.93 (3) Å]. Similar geometry restraint (SAME) was applied to disordered biphenyl moiety of **2n**. Crystallographic data for **2b**-**2e**, **2g** and **2i**-**2s** were deposited in the Cambridge Crystallographic Data Centre with CCDC no. 1476974–1476982 and 1477101–1477107 as supplementary publications. Copies of available material can be obtained free of charge, on application to CCDC, 12 Union Road, Cambridge CB2 1EZ, UK, (Fax: +44-(0)1223-336033 or e-mail: deposit@ccdc.cam.ac.uk).

### Synthesis

**Fig 1:**
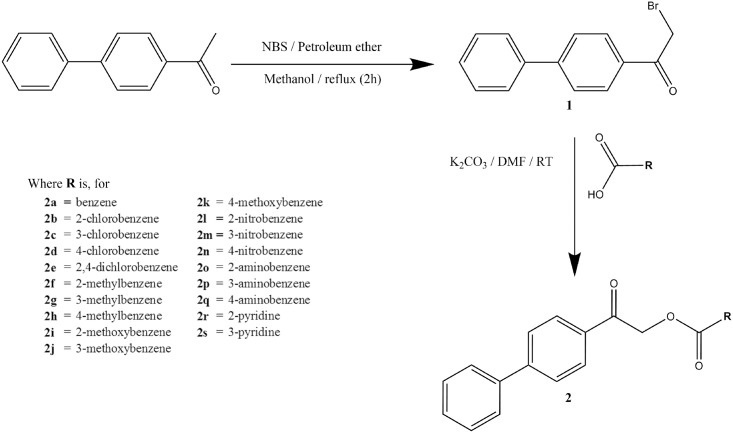
The reaction scheme for the synthesis of 2-([1,1'-biphenyl]-4-yl)-2-oxoethyl benzoates, 2(a-q), and 2-([1,1'-biphenyl]-4-yl)-2-oxoethyl pyridinecarboxylate, 2r&2s.

Target compounds were synthesized *via* a two-step reaction (Fig 1). First, 1-([1,1'-biphenyl]-4-yl)ethan-1-one was refluxed with *N*-bromosuccinimide and petroleum ether in methanol at 333K for two hours. The resultant precipitate of 1-([1,1'-biphenyl]-4-yl)-2-bromoethan-1-one (**1**) was filtered and recrystallized with ethanol. Next, **1** (0.55 g, 0.002 mol) was reacted with various carboxylic acids (0.003 mol) in the presence of potassium carbonate in DMF (5 ml) and stirred at room temperature for about four hours. The reaction progress was monitored by thin layer chromatography (TLC). The reaction mixture was poured into ice-cool water after the completion of reaction and was stirred for another 10 minutes. The precipitate obtained was filtered out and washed successively with distilled water [19]. The dried precipitate was purified using silica gel column chromatography, eluting with ethyl acetate/hexane (2:8). Suitable single crystal specimens were obtained *via* slow evaporation from various types of solvents as described below. All target compounds **2**(**a**-**s**) were synthesized in good yield and high purity. Their chemical structures were characterized by using NMR and FTIR spectroscopy. Crystal structures of all compounds except **2a**, **2f** and **2h** were determined by using single-crystal X-ray diffraction analysis.

*2-([1*,*1'-biphenyl]-4-yl)-2-oxoethyl benzoate* (**2a**): Yield: 73%; M.P. 442–444 K; FT-IR (ATR (solid) cm^-1^): 3063 (Ar C–H, v), 2936 (C–H, ν), 1718, 1696 (C = O, ν), 1599, 1451 (Ar, C–C, ν), 1277, 1234, 1123 (C–O, ν); ^1^H NMR (500 MHz, CDCl_3_): *δ* ppm δ 8.197–8.180 (d, 2H, *J* = 8.3 Hz, 17–CH, 21–CH), 8.090–8.073 (d, 2H, *J* = 8.6 Hz, 9–CH, 11–CH), 7.769–7.752 (d, 2H, *J* = 8.6 Hz, 8–CH, 12–CH), 7.675–7.661 (d, 2H, *J* = 7.1 Hz, 1–CH, 5–CH), 7.649–7.620 (t, 1H, *J* = 7.4 Hz, 19–CH), 7.530–7.496 (m, 4H, 2–CH, 4–CH, 18–CH, 20–CH), 7.463–7.434 (t, 1H, *J* = 7.1 Hz, 3–CH), 5.641 (s, 2H, 14–CH_2_); ^13^C NMR (125 MHz, CDCl_3_): *δ* 191.77 (C13), 166.11 (C15), 146.62 (C7), 139.68 (C6), 133.39 (C19), 133.01(C10), 130.02 (C9, C11), 129.44 (C16), 129.03 (C2, C4), 128.48 (C17, C21), 128.47 (C18, C20), 127.91 (C3), 127.53 (C8, C12), 127.31 (C1, C5), 66.53 (C14).

*2-([1*,*1'-biphenyl]-4-yl)-2-oxoethyl 2-chlorobenzoate* (**2b**): Solvent for growing crystal: acetone; Yield: 85%; M.P. 392–394 K; FT-IR (ATR (solid) cm^-1^): 3068 (Ar C–H, v) 2944 (C–H, ν), 1728, 1691 (C = O, ν), 1589, 1470 (Ar, C–C, ν), 1234, 1112, 1029 (C–O, ν), 736 (C–Cl, ν); ^1^H NMR (500 MHz, CDCl_3_): *δ* ppm 8.111–8.097 (d, 1H, *J* = 7.3 Hz, 21–CH), 8.085–8.068 (d, 2H, *J* = 8.6 Hz, 9–CH, 11–CH), 7.776–7.758 (d, 2H, *J* = 8.6 Hz, 8–CH, 12–CH), 7.681–7.664 (d, 2H, *J* = 8.4 Hz, 1–CH, 5–CH), 7.533–7.503 (m, 3H, 2–CH, 3–CH, 4–CH), 7.495–7.479 (d, 1H, *J* = 7.3 Hz, 18–CH), 7.466–7.437 (t, 1H, *J* = 7.4 Hz, 20–CH), 7.417–7.388 (t, 1H, *J* = 7.3 Hz, 19–CH), 5.656 (s, 2H, 14–CH_2_); ^13^C NMR (125 MHz, CDCl_3_): *δ* ppm 191.43 (C13), 165.27 (C15), 146.76 (C7), 140.26 (C6), 138.46 (C10), 134.14 (C19), 133.04 (C17), 132.88 (C16), 132.00 (C20), 131.24 (C3), 129.10 (C9, C11), 128.49 (C2, C4), 128.27 (C18), 127.59 (C8, C12), 127.35 (C1, C5), 126.78 (C21) 66.55 (C14)

*2-([1*,*1'-biphenyl]-4-yl)-2-oxoethyl 3-chlorobenzoate* (**2c**): Solvent for growing crystal: acetone; Yield: 80%; M.P. 427–429 K; FT-IR (ATR (solid) cm^-1^): 3076 (Ar C-H, v), 2941 (C–H, ν), 1727, 1696 (C = O, ν), 1600, 1412 (Ar, C–C, ν), 1295, 1232, 1131 (C–O, ν), 744 (C–Cl, ν); ^1^H NMR (500 MHz, CDCl_3_): *δ* ppm 8.169 (s, 1H, 17–CH), 8.080–8.063 (d, 3H, *J* = 8.6 Hz, 9–CH, 11–CH, 21–CH), 7.774–7.757 (d, 2H, *J* = 8.6 Hz, 8–CH, 12–CH), 7.676–7.662 (d, 2H, J = 7.1 Hz, 1–CH, 5–CH), 7.618–7.600 (d, 1H, *J* = 9.1 Hz, 19–CH), 7.534–7.504 (t, 2H, *J* = 7.1 Hz, 2–CH, 4–CH), 7.473–7.441 (m, 2H, 3–CH, 20–CH), 5.651 (s, 2H, 14–CH_2_); ^13^C NMR (125 MHz, CDCl_3_): *δ* ppm 191.31 (C13), 164.94 (C15), 146.76 (C7), 139.62 (C6), 134.66 (C10), 133.44 (C19), 132.81 (C10), 131.17 (C16), 130.08 (C20), 129.82 (C17), 129.05 (C9, C11), 128.50 (C21), 128.44 (C2, C4), 128.15 (C3), 127.58 (C8, C12), 127.32 (C1, C5) 66.72 (C14).

*2-([1*,*1'-biphenyl]-4-yl)-2-oxoethyl 4-chlorobenzoate* (**2d**): Solvent for growing crystal: acetone, acetonitrile (1:1 v/v); Yield: 84%; M.P. 435–437 K; FT-IR (ATR (solid) cm^-1^): 3066 (Ar C-H, v), 2944 (C–H, ν), 1719, 1690 (C = O, ν), 1598, 1425 (Ar, C–C, ν), 1274, 1232, 1107 (C–O, ν), 760 (C–Cl, ν); ^1^H NMR (500 MHz, CDCl_3_): *δ* ppm 8.134–8.116 (d, 2H, *J* = 8.8 Hz, 17–CH, 21–CH), 8.080–8.063 (d, 2H, *J* = 8.6 Hz, 9–CH, 11–CH), 7.772–7.755 (d, 2H, *J* = 8.6 Hz, 8–CH, 12–CH), 7.675–7.661 (d, 2H, *J* = 7.1 Hz, 1–CH, 5–CH), 7.533–7.503 (t, 2H, *J* = 7.1 Hz, 2–CH, 4–CH), 7.498–7.480 (d, 2H, *J* = 8.8 Hz, 18–CH, 20–CH), 7.467–7.437 (t, 1H, *J* = 7.1 Hz, 3–CH), 5.639 (s, 2H, 14–CH_2_); ^13^C NMR (125 MHz, CDCl_3_): *δ* ppm 191.52 (C13), 16532 (C15), 146.77 (C7), 139.95 (C6), 139.66 (C19), 132.93 (C10), 131.44 (C9, C11), 129.05 (C17, C21), 128.86 (C18, C20), 128.50 (C16), 128.44 (C2, C4), 127.89 (C3), 127.57 (C8, C12), 127.32 (C1, C5), 66.61 (C14).

*2-([1*,*1'-biphenyl]-4-yl)-2-oxoethyl 2*,*4-dichlorobenzoate* (**2e**): Solvent for growing crystal: chloroform; Yield: 79%; M.P. 383–385 K; FT-IR (ATR (solid) cm^-1^): 3092 (Ar C-H, v), 2933 (C–H, ν), 1735, 1693 (C = O, ν), 1603, 1415 (Ar, C–C, ν), 1229, 1134, 1105 (C–O, ν), 763 (C–Cl, ν); ^1^H NMR (500 MHz, CDCl_3_): *δ* ppm 8.078–8.052 (m, 3H, 9–CH, 11–CH, 17–CH), 7.769–7.752 (d, 2H, *J* = 8.5 Hz, 8–CH, 12–CH), 7.672–7.658 (d, 2H, *J* = 7.1 Hz, 1–CH, 5–CH), 7.546 (s, 1H, 20–CH), 7.530–7.500 (t, 2H, *J* = 7.1 Hz, 2–CH, 4–CH), 7.465–7.436 (t, 1H, *J* = 7.1 Hz, 3–CH), 7.392–7.375 (d, 1H, *J* = 8.5 Hz, 18–CH), 5.644 (s, 2H, 14–CH_2_); ^13^C NMR (125 MHz, CDCl_3_): *δ* ppm 191.17 (C13), 164.05 (C15), 146.79 (C7), 139.58 (C6), 138.84 (C19), 135.38 (C10), 133.09 (C21), 132.75 (C17), 131.11 (C18), 139.05 (C9, C11), 128.52 (C20), 128.43 (C2, C4), 127.57 (C8, C12), 127.50 (C16), 127.31 (C1, C5), 127.14 (C3), 66.74 (C14).

*2-([1*,*1'-biphenyl]-4-yl)-2-oxoethyl 2-methylbenzoate* (**2f**): Yield: 68%; M.P. 368–370 K; FT-IR (ATR (solid) cm^-1^): 3063 (Ar C-H, v), 2931 (C–H, ν), 1723, 1696 (C = O, ν), 1605, 1419 (Ar, C–C, ν), 1263, 1234, 1099 (C–O, ν); ^1^H NMR (500 MHz, CDCl_3_): *δ* ppm 8.126–8.112 (d, 1H, *J* = 7.3 Hz, 21–CH), 8.091–8.073 (d, 2H, *J* = 8.6 Hz, 9–CH, 11–CH), 7.770–7.753 (d, 2H, *J* = 8.6 Hz, 8–CH, 12–CH), 7.677–7.663 (d, 2H, *J* = 7.1 Hz, 1–CH, 5–CH), 7.530–7.500 (t, 2H, *J* = 7.1 Hz, 2–CH, 4–CH), 7.485–7.433 (m, 3H, 3–CH, 18–CH), 7.331–7.301 (t, 2H, *J* = 7.3 Hz, 19–CH, 20–CH), 5.621 (s, 2H, 14–CH_2_), 2.679 (s, 3H, 22–CH_3_); ^13^C NMR (125 MHz, CDCl_3_): *δ* ppm 189.12 (C13), 164.05 (C15), 146.67 (C7), 140.69 (C6), 139.55 (C17), 133.05 (C10), 132.40 (C19), 131.72 (C18), 131.03 (C21), 129.03 (C9, C11), 128.85 (C16), 128.45 (C2, C4), 127.71 (C3), 127.53 (C8, C12), 127.31 (C1, C5), 125.81 (C20), 66.28 (C14), 21.71 (C22).

*2-([1*,*1'-biphenyl]-4-yl)-2-oxoethyl 3-methylbenzoate* (**2g**): Solvent for growing crystal: acetone, ethanol and acetonitrile (1:1:1 v/v/v); Yield: 79%; M.P. 413–415 K; FT-IR (ATR (solid) cm^-1^): 3033 (Ar C-C, v), 2942 (C–H, ν), 1712, 1696 (C = O, ν), 1602, 1416, (Ar, C–H, ν), 1279, 1196, 1118 (C–O, ν); ^1^H NMR (500 MHz, CDCl_3_): *δ* ppm δ 8.090–8.073 (d, 2H, *J* = 8.6 Hz, 9–CH, 11–CH), 8.008 (s, 1H, 17–CH), 7.997–7.981 (d, 1H, *J* = 7.8 Hz, 21–CH), 7.768–7.751 (d, 2H, *J* = 8.6 Hz, 8–CH, 12–CH), 7.676–7.660 (d, 2H, *J* = 7.5 Hz, 1–CH, 5–CH), 7.531–7.501 (t, 2H, *J* = 7.5 Hz, 2–CH, 4–CH), 7.464–7.434 (m, 2H, 19–CH, 20–CH), 7.410–7.380 (t, 1H, *J* = 7.5 Hz, 3–CH), 5.633 (s, 2H, 14–CH_2_), 2.454 (s, 3H, 22–CH_3_); ^13^C NMR (125 MHz, CDCl_3_): *δ* ppm 191.81 (C13), 166.28 (C15), 146.62 (C7), 139.68 (C6), 138.29 (C18), 134.17 (C19), 133.02 (C10), 130.52 (C21), 129.33 (C16), 129.03 (C9, C11), 128.47 (C2, C4), 128.45 (C17), 128.39 (C20), 127.53 (C8, C12), 127.32 (C1, C5), 127.17 (C3), 66.45 (C14), 21.30 (C22).

*2-([1*,*1'-biphenyl]-4-yl)-2-oxoethyl 4-methylbenzoate* (**2h**): Yield: 71%; M.P. 438–440 K; FT-IR (ATR (solid) cm^-1^): 3038 (Ar C-H, v), 2928 (C–H, ν), 1715, 1696 (C = O, ν), 1602, 1411, (Ar, C–C, ν), 1274, 1234, 1129 (C–O, ν); ^1^H NMR (500 MHz, CDCl_3_): *δ* ppm 8.086–8.065 (m, 4H, 9–CH, 11–CH, 17–CH, 21–CH), 7.763–7.746 (d, 2H, *J* = 8.6 Hz, 8–CH, 12–CH), 7.673–7.659 (d, 2H, *J* = 7.2 Hz, 1–CH, 5–CH), 7.528–7.498 (t, 2H, *J* = 7.2 Hz, 2–CH, 4–CH), 7.460–7.431 (t, 1H, *J* = 7.2 Hz, 3–CH), 7.313–7.297 (d, 2H, *J* = 7.9 Hz, 18–CH, 20–CH), 5.616 (s, 2H, 14–CH2), 2.460 (s, 3H, 22–CH_3_); ^13^C NMR (125 MHz, CDCl_3_): *δ* ppm 191.94 (C13), 166.15 (C15), 146.58 (C7), 144.14 (C19), 139.69 (C6), 133.05 (C10), 130.05 (C9, C11), 129.20 (C17, C21), 129.02 (C18, C20), 128.47 (C2, C4), 128.44 (C3), 127.52 (C8, C12), 127.31 (C1, C5), 126.67 (C16), 66.38 (C14), 21.35 (C22).

*2-([1*,*1'-biphenyl]-4-yl)-2-oxoethyl 2-methoxybenzoate* (**2i**): Solvent for growing crystal: acetone; Yield: 85%; M.P. 400–402 K; FT-IR (ATR (solid) cm^-1^): 3073 (Ar C-H, v), 2998, 2936, 2843 (C–H, ν), 1731, 1699 (C = O, ν), 1599, 1411 (Ar, C–H, ν), 1244, 1225, 1102, 1016 (C–O, ν); ^1^H NMR (500 MHz, CDCl_3_): *δ* ppm 8.088–8.072 (d, 2H, *J* = 8.3 Hz, 9–CH, 11–CH), 8.059–8.044 (d, 1H, *J* = 7.6 Hz, 17–CH), 7.762–7.745 (d, 2H, *J* = 8.3 Hz, 8–CH, 12–CH), 7.674–7.660 (d, 2H, *J* = 7.2 Hz, 1–CH, 5–CH), 7.565–7.550 (d, 1H, *J* = 7.6 Hz, 20–CH), 7.529–7.498 (t, 2H, *J* = 7.2 Hz, 2–CH, 4–CH), 7.460–7.431 (t, 1H, *J* = 7.2 Hz, 3–CH), 7.070–7.031 (m, 2H, 18–CH, 19–CH), 5.603 (s, 2H, 14–CH_2_), 3.957 (s, 3H, 22–CH_3_); ^13^C NMR (125 MHz, CDCl_3_): *δ* ppm 191.05 (C13), 165.30 (C15), 159.63 (C17), 146.50 (C7), 139.74 (C6), 134.10 (C19), 133.17 (C10), 132.30 (C21), 129.04 (C9, C11), 128.52 (C2, C4), 128.42 (C3), 127.48 (C8, C12), 127.32 (C1, C5), 120.26 (C20), 119.04 (C16), 112.09 (C18), 66.29 (C14), 56.08 (C22).

*2-([1*,*1'-biphenyl]-4-yl)-2-oxoethyl 3-methoxybenzoate* (**2j**): Solvent for growing crystal: acetone; Yield: 84%; M.P. 397–399 K; FT-IR (ATR (solid) cm^-1^): 3092 (Ar C-H, v), 2933, 2838 (C–H, ν), 1719, 1704 (C = O, ν), 1603, 1417 (Ar, C–C, ν), 1288, 1110, 1030 (C–O, ν); ^1^H NMR (500 MHz, CDCl_3_): *δ* ppm 8.088–8.071 (d, 2H, *J* = 8.6 Hz, 9–CH, 11–CH), 7.802–7.787 (d, 1H, *J* = 7.5 Hz, 21–CH), 7.769–7.752 (d, 2H, *J* = 8.6 Hz, 8–CH, 12–CH), 7.696 (s, 1H, 17–CH), 7.676–7.659 (d, 2H, *J* = 7.9 Hz, 1–CH, 5–CH), 7.531–7.501 (t, 2H, *J* = 7.9 Hz, 2–CH, 4–CH), 7.464–7.435 (t, 1H, *J* = 7.5 Hz, 20–CH), 7.431–7.399 (t, 1H, *J* = 7.9 Hz, 3–CH), 7.189–7.174 (d, 1H, *J* = 7.5 Hz, 19–CH), 5.634 (s, 2H, 14–CH_2_), 3.898 (s, 3H, 22–CH_3_); ^13^C NMR (125 MHz, CDCl_3_): *δ* ppm 191.69 (C13), 165.99 (C15), 159.62 (C17), 146.64 (C7), 139.66 (C6), 132.98 (C10), 130.68 (C16), 129.52 (C20), 129.04 (C9, C11), 128.47 (C2, C4), 127.54 (C8, C12), 127.53 (C3), 127.32 (C1, C5), 122.49 (C19), 120.15 (C21), 114.22 (C17), 66.59 (C14), 55.49 (C22).

*2-([1*,*1'-biphenyl]-4-yl)-2-oxoethyl 4-methoxybenzoate* (**2k**): Solvent for growing crystal: acetone and acetonitrile (1:1 v/v); Yield: 79%; M.P. 437–439 K; FT-IR (ATR (solid) cm^-1^): 3005 (Ar C-H, v), 2976, 2931, 2841 (C–H, ν), 1714, 1698 (C = O, ν), 1603, 1420 (Ar, C–C, ν), 1256, 1168, 1126, 1028 (C–O, ν); ^1^H NMR (500 MHz, CDCl_3_): *δ* ppm 8.150–8.133 (d, 2H, *J* = 9.0 Hz, 17–CH, 21–CH), 8.087–9.070 (d, 2H, *J* = 8.6 Hz, 9–CH, 11–CH), 7.763–7.746 (d, 2H, *J* = 8.6 Hz, 8–CH, 12–CH), 7.674–7.659 (d, 2H, *J* = 7.5 Hz, 1–CH, 5–CH), 7.529–7.499 (t, 2H, *J* = 7.5 Hz, 2–CH, 4–CH), 7.461–7.432 (t, 1H, *J* = 7.5 Hz, 3–CH), 6.994–6.976 (d, 2H, *J* = 9.0 Hz, 18–CH, 20–CH), 5.605 (s, 2H, 14–CH_2_), 3.910 (s, 3H, 22–CH_3_); ^13^C NMR (125 MHz, CDCl_3_): *δ* ppm 192.07 (C13), 165.79 (C15), 163.74 (C19), 146.56 (C7), 139.70 (C6), 133.07 (C10), 132.11 (C17, C21), 129.02 (C9, C11), 128.47 (C2, C4), 128.43 (C3), 127.51 (C8, C12), 127.31 (C1, C5), 121.80 (C16), 113.75 (C18, C20), 66.30 (C14), 55.49 (C22).

*2-([1*,*1'-biphenyl]-4-yl)-2-oxoethyl 2-nitrobenzoate* (**2l**): Solvent for growing crystal: acetone, acetonitrile (1:1 v/v); Yield: 80%; M.P. 413–415 K; FT-IR (ATR (solid) cm^-1^): 3092 (Ar C-H, v), 2941, 2864 (C–H, ν), 1743, 1690 (C = O, ν), 1603, 1423 (Ar, C–C, ν), 1529, 1343 (N–O, ν), 1290, 1240, 1123, 1078 (C–O, ν); ^1^H NMR (500 MHz, CDCl_3_): *δ* ppm 8.068–8.051 (d, 2H, *J* = 8.6 Hz, 9–CH, 11–CH), 8.039–8.011 (m, 2H, 18–CH, 19–CH), 7.797–7.762 (m, 3H, 8–CH, 12–CH, 21–CH), 7.723–7.692 (t, 1H, *J* = 7.9 Hz, 20–CH), 7.681–7.660 (d, 2H, *J* = 7.5 Hz, 1–CH, 5–CH), 7.534–7.504 (t, 2H, *J* = 7.5 Hz, 2–CH, 4–CH), 7.468–7.439 (t, 1H, *J* = 7.5 Hz, 3–CH), 5.664 (s, 2H, 14–CH_2_); ^13^C NMR (125 MHz, CDCl_3_): *δ* ppm 190.92 (C13), 165.10 (C15), 147.79 (C7), 146.88 (C17), 139.57 (C6), 133.28 (C20), 132.62 (C10), 131.94 (C19), 130.40 (C21), 129.05 (C9, C11), 128.53 (C3), 128.45 (C2, C4), 127.61 (C8, C12), 127.37 (C16), 127.32 (C1, C5), 124.05 (C18), 67.36 (C14).

*2-([1*,*1'-biphenyl]-4-yl)-2-oxoethyl 3-nitrobenzoate* (**2m**): Solvent for growing crystal: acetone, acetonitrile (1:1 v/v); Yield: 82%; M.P. 426–428 K; FT-IR (ATR (solid) cm^-1^): 3092 (Ar, C–H, ν), 2925, 2880 (C–H, ν), 1738, 1690 (C = O, ν), 1603, 1441 (Ar, C–C, ν), 1537, 1348 (N–O, ν), 1229, 1136 (C–O, ν); ^1^H NMR (500 MHz, CDCl_3_): *δ* ppm 9.027 (s, 1H, 21–CH), 8.525–8.492 (t, 2H, *J* = 8.0 Hz, 18–CH, 19–CH), 8.085–8.068 (d, 2H, *J* = 8.6 Hz, 9–CH, 11–CH), 7.787–7.769 (d, 2H, *J* = 8.6 Hz, 8–CH, 12–CH), 7.751–7.719 (t, 1H, *J* = 8.0 Hz, 17–CH), 7.680–7.666 (d, 2H, *J* = 7.2 Hz, 1–CH, 5–CH), 7.538–7.508 (t, 2H, *J* = 7.2 Hz, 2–CH, 4–CH), 7.473–7.443 (t, 1H, *J* = 7.2 Hz, 3–CH), 5.715 (s, 2H, 14–CH_2_); ^13^C NMR (125 MHz, CDCl_3_): *δ* ppm 190.88 (C13), 164.07 (C15), 148.35 (C18), 146.93 (C7), 139.56 (C6), 135.67 (C21), 132.63 (C10), 131.25 (C16), 129.77 (C20), 129.07 (C9, C11), 128.56 (C3), 128.44 (C2, C4), 127.82 (C19), 127.63 (C8, C12), 127.32 (C1, C5), 126.06 (C17), 67.05 (C14).

*2-([1*,*1'-biphenyl]-4-yl)-2-oxoethyl 4-nitrobenzoate* (**2n**): Solvent for growing crystal: acetone, ethanol and acetonitrile (1:1:1 v/v/v); Yield: 75%; M.P. 459–461 K; FT-IR (ATR (solid) cm^-1^): 3116 (Ar, C–H, ν), 2931, 2859 (C–H, ν), 1733, 1696 (C = O, ν), 1603, 1420 (Ar, C–C, ν), 1518, 1348 (N–O, ν), 1282, 1237, 1120, 1105 (C–O, ν); ^1^H NMR (500 MHz, CDCl_3_): *δ* ppm 8.361 (s, 4H, 17–CH, 18–CH, 20–CH, 21–CH), 8.080–8.063 (s, 2H, *J* = 8.6 Hz, 9–CH, 11–CH), 7.784–7.767 (d, 2H, *J* = 8.6 Hz, 8–CH, 12–CH), 7.677–7.663 (d, 2H, *J* = 7.2 Hz, 1–CH, 5–CH), 7.536–7.506 (t, 2H, *J* = 7.2 Hz, 2–CH, 4–CH), 7.472–7.443 (t, 1H, *J* = 7.2 Hz, 3–CH), 5.701 (s, 2H, 14–CH_2_); ^13^C NMR (125 MHz, CDCl_3_): *δ* ppm 190.87(C13), 164.28 (C15), 150.81 (C19), 146.95 (C7), 139.53 (C6), 134.86 (C16), 132.63 (C10), 131.15 (C17, C21), 129.07 (C9, C11), 128.57 (C3), 128.43 (C2, C4), 127.63 (C8, C12), 127.31 (C1, C5), 123.65 (C18, C20), 67.05 (C14).

*2-([1*,*1'-biphenyl]-4-yl)-2-oxoethyl 2-aminobenzoate* (**2o**): Solvent for growing crystal: acetone, ethanol and acetonitrile (1:1:1 v/v); Yield: 73%; M.P. 445–447 K; FT-IR (ATR (solid) cm^-1^): 3479, 3368 (N–H, ν), 3058 (Ar C-H, v), 2933 (C–H, ν), 1688 (C = O, ν), 1619, 1423 (Ar, C–C, ν), 1603 (N–H, δ), 1232, 1145 (C–O, ν); ^1^H NMR (500 MHz, CDCl_3_): *δ* 8.093–8.076 (d, 2H, *J* = 8.6 Hz, 9–CH, 11–CH), 8.065–8.048 (d, 1H, *J* = 8.4 Hz, 21–CH), 7.767–7.750 (d, 2H, *J* = 8.6 Hz, 8–CH, 12–CH), 7.678–7.663 (d, 2H, *J* = 7.3 Hz, 1–CH, 5–CH), 7.530–7.500 (t, 2H, *J* = 7.3 Hz, 2–CH, 4–CH), 7.462–7.433 (t, 1H, *J* = 7.3 Hz, 3–CH), 7.350–7.316 (t, 1H, *J* = 8.4 Hz, 19–CH), 6.735–6.706 (m, 2H, 18–CH, 20–CH), 5.592 (s, 2H, 14–CH_2_); ^13^C NMR (125 MHz, CDCl_3_): *δ* ppm 192.01 (C13), 167.36 (C15), 150.67 (C17), 146.61 (C7), 139.67 (C6), 134.59 (C19), 133.00 (C10), 131.67 (C21), 129.03 (C9, C11), 128.49 (C2, C4), 128.45 (C3), 127.53 (C8, C12), 127.32 (C1, C5), 116.76 (C18), 116.51 (C20), 110.10 (C16), 66.08 (C14).

*2-([1*,*1'-biphenyl]-4-yl)-2-oxoethyl 3-aminobenzoate* (**2p**): Solvent for growing crystal: acetone, ethanol (1:1 v/v); Yield: 78%; M.P. 424–426 K; FT-IR (ATR (solid) cm^-1^): 3458, 3356 (N–H, ν), 3038 (Ar C-H, v), 2939 (C–H, ν), 1707, 1685 (C = O, ν), 1632, 1403 (Ar, C–C, ν), 1602 (N–H, δ), 1303, 1223, 1110 (C–O, ν); ^1^H NMR (500 MHz, CDCl3): *δ* ppm 8.083–8.066 (d, 2H, *J* = 8.6 Hz, 9–CH, 11–CH), 7.764–7.747 (d, 2H, *J* = 8.6 Hz, 8–CH, 12–CH), 7.673–7.657 (d, 2H, *J* = 7.4 Hz, 1–CH, 5–CH), 7.581–7.566 (d, 1H, *J* = 7.8 Hz, 21–CH), 7.528–7.498 (t, 1H, *J* = 7.4 Hz, 2–CH, 4–CH), 7.486 (s, 1H, 17–CH), 7.460–7.431 (t, 1H, *J* = 7.4 Hz, 3–CH), 7.297–7.265 (t, 1H, *J* = 7.8 Hz, 20–CH), 6.936–6.920 (d, 1H, *J* = 7.8 Hz, 19–CH), 5.608 (s, 2H, 14–CH_2_); ^13^C NMR (125 MHz, CDCl3): *δ* ppm 191.88 (C13), 165.29 (C15), 152.04 (C18), 146.01 (C7), 139.74 (C6), 135.75 (C10), 130.40 (C20), 129.41 (C16), 129.06 (C9, C11), 128.58 (C2, C4), 128.48 (C3), 127.54 (C8, C12), 127.33 (C1, C5), 120.21 (C19), 119.89 (C21), 116.12 (C16), 66.45 (C14).

*2-([1*,*1'-biphenyl]-4-yl)-2-oxoethyl 4-aminobenzoate* (**2q**): Solvent for growing crystal: acetone Yield: 83%; M.P. 477–479 K; FT-IR (ATR (solid) cm^-1^): 3437, 3342, 3219 (N–H, ν), 3028 (Ar C-H, v), 2931 (C–H, ν), 1683 (C = O, ν), 1629, 1419 (Ar, C–C, ν), 1594 (N–H, δ), 1282, 1236, 1169, 1126 (C–O, ν); ^1^H NMR (500 MHz, CDCl_3_): *δ* ppm 8.082–8.065 (d, 2H, *J* = 8.5 Hz, 9–CH, 11–CH), 7.999–7.981 (d, 2H, *J* = 8.8 Hz, 17–CH, 21–CH), 7.754–7.737 (d, 2H, *J* = 8.5 Hz, 8–CH, 12–CH), 7.669–7.655 (d, 2H, *J* = 7.2 Hz, 1–CH, 5–CH), 7.524–7.494 (t, 2H, *J* = 7.2 Hz, 2–CH, 4–CH), 7.455–7.426 (t, 1H, *J* = 7.2 Hz, 3–CH), 6.705–6.688 (d, 2H, *J* = 8.8 Hz, 18–CH, 20–CH), 5.568 (s, 2H, 14–CH_2_); ^13^C NMR (125 MHz, CDCl_3_): *δ* ppm 191.66 (C13), 166.18 (C15), 151.22 (C18), 146.01 (C7), 139.74 (C6), 133.00 (C10), 132.16 (C17, C21), 129.02 (C9, C11), 128.49 (C2, C4), 128.40 (C3), 127.49 (C8, C12), 127.32 (C1, C5), 118.90 (C16), 113.85 (C18, C20), 66.21 (C14).

*2-([1*,*1'-biphenyl]-4-yl)-2-oxoethyl picolinate* (**2r**): Solvent for growing crystal: acetone, acetonitrile (1:1 v/v); Yield: 77%; M.P. 389–391 K; FT-IR (ATR (solid) cm^-1^): 3061 (Ar C-H, v), 2931 (C–H, ν), 1741, 1717, 1693 (C = O, ν), 1603, 1404 (Ar, C–C, ν), 1309 (C–N, ν) 1234, 1131 (C–O, ν); ^1^H NMR (500 MHz, CDCl_3_): *δ* ppm 8.851–8.837 (d, 1H, *J* = 7.5 Hz, 17–CH), 8.277–8.262 (d, 1H, *J* = 7.5 Hz, 20–CH), 8.085–8.068 (d, 2H, *J* = 8.5 Hz, 9–CH, 11–CH), 7.935–7.904 (t, 1H, *J* = 7.5 Hz, 19–CH), 7.766–7.748 (d, 2H, *J* = 8.5 Hz, 8–CH, 12–CH), 7.669–7.655 (d, 2H, *J* = 7.2 Hz, 1–CH, 5–CH), 7.571–7.547 (t, 1H, *J* = 7.5 Hz, 18–CH), 7.526–7.496 (t, 2H, *J* = 7.2 Hz, 2–CH, 4–CH), 7.459–7.430 (t, 1H, *J* = 7.2 Hz, 3–CH) 5.735 (s, 2H, 14–CH_2_); ^13^C NMR (125 MHz, CDCl_3_): *δ* ppm 190.92 (C13), 164.64 (C15), 149.99 (C16), 147.41 (C7), 146.71 (C20), 139.65 (C6), 137.13 (C18), 132.83 (C10), 129.03 (C9, C11), 128.51 (C2, C4), 128.45 (C3), 127.55 (C8, C12), 127.31 (C1, C5), 127.28 (C19), 125.69 (C17), 67.18 (C14).

*2-([1*,*1'-biphenyl]-4-yl)-2-oxoethyl nicotinate* (**2s**): Solvent for growing crystal: acetone, ethanol and acetonitrile (1:1:1 v/v/v); Yield: 72%; M.P. 389–391 K;FT-IR (ATR (solid) cm^-1^):3034 (Ar C-H, v), 2928 (C–H, ν), 1735, 1722, 1696 (C = O, ν), 1595, 1417 (Ar, C–H, ν), 1327 (C–N, ν) 1285, 1134 (C–O, ν);^1^H NMR (500 MHz, CDCl_3_): *δ* ppm 9.483 (s, 1H, 20–CH), 8.989–8.978 (d, 1H, *J* = 5.4 Hz, 19–CH), 8.956–8.940 (d, 1H, *J* = 5.4 Hz, 17–CH), 8.066–8.049 (d, 2H, *J* = 8.6 Hz, 9–CH, 11–CH), 7.978–7.955 (t, 1H, *J* = 5.4 Hz, 18–CH), 7.795–7.778 (d, 2H, *J* = 8.6 Hz, 8–CH, 12–CH), 7.679–7.665 (d, 2H, *J* = 7.4 Hz, 1–CH, 5–CH), 7.540–7.510 (t, 2H, *J* = 7.4 Hz, 2–CH, 4–CH), 7.477–7.448 (t, 1H, *J* = 7.4 Hz, 3–CH) 5.759 (s, 2H, 14–CH_2_); ^13^C NMR (125 MHz, CDCl_3_): *δ* ppm 189.96 (C13), 165.89 (C15), 147.26 (C7), 143.37 (C6), 139.55 (C19), 138.62 (C20), 133.22 (C10), 132.28 (C17), 129.08 (C9, C11), 128.95 (C18), 128.66 (C3), 128.43 (C2, C4), 127.71 (C8, C12), 127.32 (C1, C5) 126.14 (C16), 67.78 (C14).

### Anti-tyrosinase assay

The evaluations of anti-tyrosinase activities of biphenyl esters **2**(**a**-**s**) were carried out according to the methods reported by Nithitanakool *et al*. (2009) with some modifications [20]. Briefly, the biphenyl esters with concentrations of 50, 100 and 250 μg/mL were diluted with 40 μL of acetone and mixed with 80 μL of mushroom tyrosinase (100 U/mL) in 0.1 M PBS (pH 6.8) solution. A similar volume of acetone with tyrosinase was used as control. Each biphenyl ester and control was prepared in triplicate. The mixtures were incubated at 37°C for 10 min. Then, 40 μL of 0.01 M L-DOPA solution was added and further incubated at 37°C for 25 min. The absorbance was measured at 475 nm using a microplate reader. Kojic acid was used as the standard drug. The percentage of inhibition of tyrosinase enzyme was calculated by using the following formula:

$$\%\;\text{Inhibition}=\;\frac{\text{Absorbance of control}-\text{Absorbance of sample}}{\text{Absorbance of control}}\;\text{x}\;100$$

### Statistical analysis

The results of anti-tyrosinase assay were expressed as mean ± standard deviation (SD) and were labeled if p < 0.05 by using ANOVA of IBM SPSS Statistics for Windows, Version 23.0 (IBM, New York).

### Docking protocol

The crystal structures of tyrosinase from *Bacillus megaterium* (TyrBm) in complex with inhibitor kojic acid (PDB entry: 3NQ1) [21] and biphenyl esters (**2i**, **2o**, **2p**, **2r** and **2s**) were used as target and ligands, respectively, for molecular docking using Genetic Optimization for Ligand Docking (GOLD) package 5.4.1 [22–24]. Genetic algorithm (GA) was used to explore the ligand-protein binding space and the conformational flexibility of ligand inside the protein. A spherical binding site with a radius of 6 Å was used across residues Phe197, Pro201, Asn205 and Arg209 in the active-site entrance. 100 GA runs were carried out and the top 100 ranked docking poses were scored using the Piecewise Linear Potential (PLP) scoring function. Default values were used for all other parameters. The intermolecular interaction of the best scored pose of each ligand was analyzed and illustrated using the Discovery Studio 4.5 software [25].

All spectral, crystallography data, crystal packing and tyrosinase assay data are described in detail in the S1 Dataset.

## Results and discussion

### Spectroscopic analysis

The IR spectra of biphenyl esters **2**(**a**-**s**) showed absorption bands above 3000 cm^-1^, indicating the presence of unsaturated C–H (benzene and biphenyl) groups, whereas the aromatic v(C = C) were shown near 1600 cm^-1^ and 1410 cm^-1^. The methyl (–CH_3_) and methylene (–CH_2_–) group’s C–H stretching were observed around 2970 and 2940 cm^-1^. In addition, distinct v(C = O) and v(C–O) bands were found in the range of 1743–1683 cm^-1^ and 1300–1028 cm^-1^. Absorption band for aryl halides were revealed at far right of the spectra, near 750 cm^-1^. The N = O stretching (**2l-2m**), N–H stretching in (**2o-2q**) and C–N stretching in (**2r**-**2s**) were observed at ~1530 cm^−1^, ~3450 cm^−1^ and ~1310 cm^−1^, respectively [26, 27]. The ^1^H NMR spectra showed presences of –CH2– protons centering around *δ*≈ 5.65 ppm and revealed two well-resolved sets of doublet centering around *δ*≈ 8.09 and 7.77 ppm with the integration values of 2:2, ascribed to the -CH- protons of second phenyl ring. The first phenyl ring was shown as a doublet and two triplet near *δ*≈7.67, 7.53 and 7.47 ppm with the integration values of 2:2:1. Furthermore, the benzene protons were revealed at down-field region in the ^1^H NMR spectra with different set of multiplicity and integration values due to different position of substituent. Biphenyl and benzene rings can be distinguished by their identical *J*-coupling values. In addition, protons of –CH_3_ and –OCH_3_ substitutions in compounds **2**(**f**-**k**) were revealed at the up-field region near *δ*≈2.50 and 3.90 ppm. Based on the integration values, numbers of protons are in agreement with the proposed values. ^13^C NMR spectra of **2**(**a**-**s**) showed three distinct sets of carbonyl carbon, aromatic carbon and saturated carbon signals. In the down-field region, both *δ*(C = O) and *δ*(COO) carbonyl signals are centering around *δ*≈191 and 165 ppm, respectively, whereas the –CH_2_– saturated carbon signals are at the up-field region located around *δ*≈66 ppm. The aromatic carbon signals of biphenyl and benzene groups were found in the range of *δ*≈152 to 110 ppm. The –CH_3_ and –OCH_3_ carbon signals of compounds **2**(**f**-**k**) were located in the up-field region centering at *δ*≈21 and 56 ppm, respectively [28, 29].

### Single crystal structure commentary

**Fig 2:**
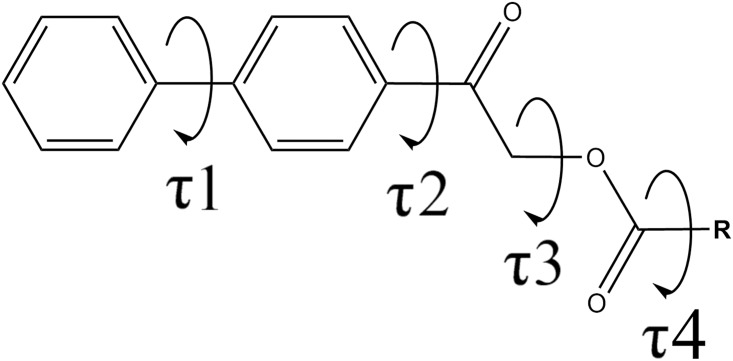
General scheme of biphenyl esters with torsion angles τ1, τ2, τ3 and τ4.

The asymmetric unit (*Z*’) of all studied compounds consists of a crystallographic independent molecule except **2m**, **2r**, and **2s**, which each consists of two crystallographic independent molecules (denoted as molecules *A* and *B*, respectively). The molecular conformation of biphenyl esters (Fig 2, Table 1) can be characterized by four degree-of-freedom, which are torsion angles C5—C6—C7—C12 (τ1), C9—C10—C13—C14 (τ2), C13—C14—O1—C15 (τ3) and O1—C15—C16—C17 (τ4).

**Table 1:** Summary of torsion angles*.

Compound	Substituent	τ1	τ2	τ3	τ4
**2b**	2-chlorobenzene	-176.57	-160.15	70.26	172.44
**2c**	3-chlorobenzene	-27.21	172.23	76.16	8.26
**2d**	4-chlorobenzene	149.13	173.43	73.32	154.95
**2e**	2,4-dichlorobenzene	-151.14	-175.31	77.09	174.74
**2g**	3-methylbenzene	-176.21	2.23	76.32	0.04
**2i**	2-methoxybenzene	-127.41	-170.83	77.17	-148.66
**2j**	3-methoxybenzene	149.09	7.15	-75.65	-6.36
**2k**	4-methoxybenzene	178.39	-6.54	-72.21	-178.03
**2l**	2-nitrobenzene	-177.09	6.76	76.59	-101.32
**2m**	3-nitrobenzene	-80.25, -109.38	5.94, 164.3	-79.04, 78.89	173.27, 173.28
**2n**	4-nitrobenzene	-178.13	-5.47	83.95	-158.17
**2o**	2-aminobenzene	149.63	-14.78	77.31	169.15
**2p**	3-aminobenzene	-156.75	170.24	89.69	1.4
**2q**	4-aminobenzene	161.03	12.88	71.34	-5.13
**2r**	2-pyridine	-164.34, -178.05	-176.66, -33.91	-78.65, -79.57	177.08, -16.00
**2s**	3-pyridine	141.69, 145.88	5.35, 26.92	74.9, -77.11	16.14, 1.67

* τ1 = Torsion angle of C5—C6—C7—C12; τ2 = Torsion angle of C9—C10—C13—C14; τ3 = Torsion angle of C13—C14—O1—C15; τ4 = Torsion angle of O1—C15—C16—C17.

From the classical point of view, the planarity of biphenyl ring is distorted by the non-bonded steric repulsion force between two *ortho*-hydrogen atoms [30]. However, molecular X-ray structure showed τ1 varied from almost perpendicular (τ1 = -80.25° and -109.38° in **2m**) to almost planar (τ1 = 178.39° in **2k**) which opposed to the classical view. Instead of steric repulsion force between the *ortho*-hydrogen atoms, the planarity of τ1 in compounds **2d**, **2i**, **2j**, **2m** and **2p** might be influenced by C—H···π interaction in the crystal packing. The torsion angle between biphenyl moiety and adjacent carbonyl group, C9—C10—C13—C14 (τ2), is nearly planar for most of the compounds and the largest deviation from planarity is observed in molecule *B* of compound **2r** (τ2 = -33.91°). Torsion angle τ3, which interconnecting two carbonyl groups, in phenacyl benzoates tends to adopt two types of conformations, either *synclinal* or *periplanar* [19]. However, τ3 for all crystals in this report only adopts *synclinal* conformation, ranging from 70.26° to 89.69°, which is similar to adamantyl-based ester derivatives [31]. The torsion angle between carboxylate group and the attached phenyl ring, O1—C15—C16—C17 (τ4), is observed in the ranges from 0.04° to 16.14° and 101.32° to 178.03°. The torsion angle τ4 of compound with methoxy– (**2i**) or nitro—substituted (**2l**) at *ortho*–position is largely twisted due to the steric repulsion between *ortho*–substituent and adjacent carbonyl oxygen atom. The benzoate groups of **2b** (2-chlorobenzene), **2d** (2,4-dichlorobenzene) and **2o** (2-aminobenzene) are nearly planar with angle τ4 of 172.44°, 174.74° and 169.15°, respectively. The *ortho*-amino substituent in **2o** forms a strong intramolecular N–H···O hydrogen bond with the adjacent carbonyl group, featuring a *S*(6) ring motif.

### Crystal packing similarity and structural occupancy

**Fig 3:**
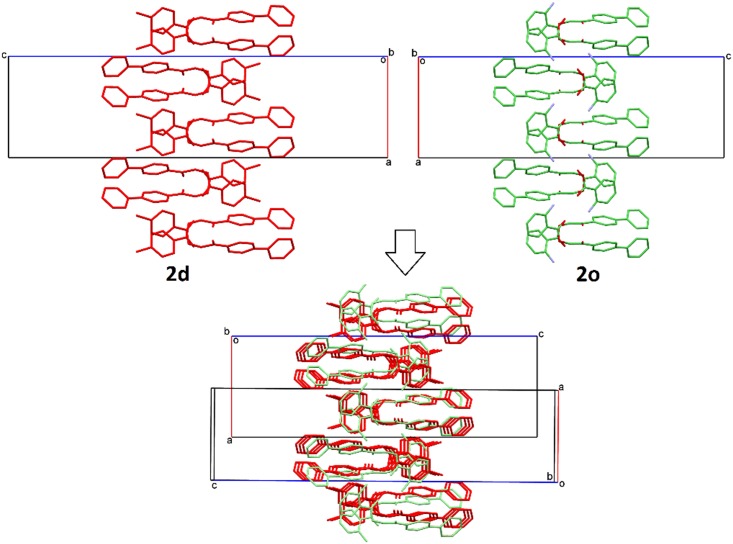
Partial crystal packing of 2d and 2o and their overlay diagram.

A Cambridge Structure Database (CSD) search using phenacyl benzoate was performed to locate previously reported phenacyl benzoate and adamantyl benzoate derivatives and 58 similar structures were found. In order to identify the effect of the replacement of phenyl ring with relatively more electron rich biphenyl rings on the crystal packing similarity and structure occupancy, sixteen of the present biphenyl benzoate derivatives were compared with 42 reported phenacyl benzoate derivatives and sixteen adamantyl benzoate derivatives. In contrast to the high occurrence of isostructures in adamantanyl benzoate derivatives [31], there is only a pair of isostructural crystals (**2d** and **2o**) (Fig 3) is observed in the present work.

**Fig 4:**
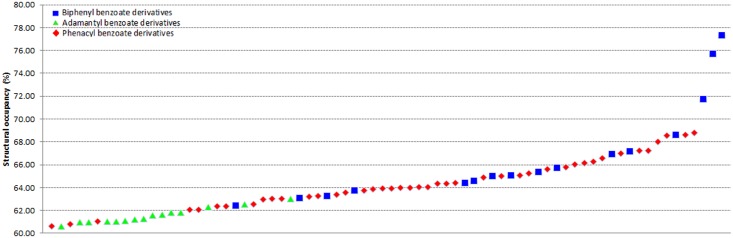
Structural occupancy comparison of biphenyl benzoates, phenacyl benzoates and adamantyl benzoates.

The comparison of crystal structure occupancy between the search results and the present compounds are summarized in Table 2. The introduction of adamantane moiety in phenacyl benzoate had reduced the occurrence of π···π interactions as compared to phenacyl benzoates which consist of two terminal phenyl rings. In this study, the replacement of adamantane moiety with biphenyl moiety encouraged the formation of weak intermolecular π···π and C–H···π interactions in crystal packing. Thus, the packing coefficient of most of the present compounds (12 out of 16) laid above 64% and some are even higher than phenacyl benzoates (**2c** = 71%, **2m** = 75% and **2r** = 77%) (Fig 4).

**Table 2:** Summary of structural occupancy of present and reported compounds.

Compound	Packing coefficient (%)	Compound	Packing coefficient (%)	Compound	Packing coefficient (%)
**2b**	64.59	**BUTPOX**[31]	61.08	**EVEGOC**[32]	63.22
**2c**	71.73	**BUTPUD**[31]	61.07	**EVEVEH**[33]	63.04
**2d**	65.37	**BUTQAK**[31]	61.85	**GARCEJ**[34]	65.80
**2e**	63.11	**BUTQEO**[31]	63.05	**GITHUN**[35]	64.40
**2g**	63.28	**BUTQIS**[31]	61.24	**IDIWID** [36]	65.26
**2i**	65.07	**BUTQOY**[31]	61.81	**KULLIO** [37]	62.07
**2j**	66.93	**BUTQUE**[31]	60.61	**MANGIR**[38]	61.06
**2k**	63.76	**AZULUD**[39]	63.85	**OBOYIP**[40]	67.22
**2l**	65.74	**BOQXOW** [41]	66.55	**OCAKUA** [42]	63.92
**2m**	75.69	**CIQNEW**[43]	64.07	**OCAQUG**[44]	66.98
**2n**	64.99	**CIXVUC**[19]	63.94	**OCEFEJ**[45]	68.55
**2o**	62.42	**CIXWAJ**[19]	64.33	**PAXCOI** [46]	66.18
**2p**	67.17	**CIXWEN**[19]	62.08	**PECZAA**[47]	64.37
**2q**	68.61	**CIXWIR**[19]	63.98	**PODQIK**[48]	60.66
**2r**	77.33	**CIYCAQ**[19]	67.27	**PODRAD** [49]	63.77
**2s**	64.42	**CIYCEU**[19]	65.07	**USIWID**[50]	62.53
**BUVCIG**[31]	61.11	**CIYCIY**[19]	68.83	**USIWID01** [51]	62.37
**BUVCOM**[31]	61.32	**CIYCOE**[19]	62.96	**USIWOJ**[52]	66.04
**BUVCUS**[31]	62.33	**CIYFUN**[19]	62.38	**VOBYUI**[53]	63.97
**BUVDAZ**[31]	62.53	**CIYGAU**[19]	64.89	**WIGTUD** [54]	64.99
**BUVDED**[31]	60.79	**EVAFOX**[55]	68.00	**YAFWEJ**[56]	66.25
**BUVDIH** [31]	60.97	**EVAJAN**[57]	65.64	**YAFZAI**[58]	68.65
**BUVDON**[31]	61.68	**EVAJIV**[59]	64.03	**YAHGUL**[60]	63.55
**BUVDUT**[31]	61.02	**EVAZEH**[61]	63.03	**YAHYOX**[62]	63.37
**BUVFAB**[31]	61.58	**EVEGIW**[63]	63.25		

### Anti-tyrosinase activities

**Fig 5:**
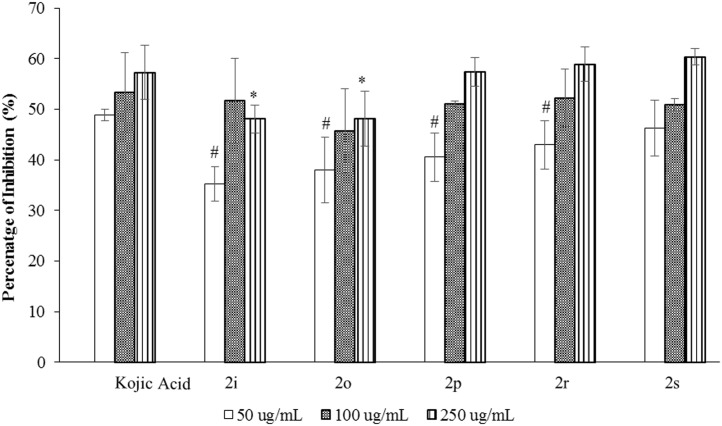
The percentage of tyrosinse inhibition (mean±SD) with n = 3. # and * indicate p<0.05.

In the present study, all synthesized compounds were screened for tyrosine inhibitory activity. Five out of nineteen compounds (**2i**, **2o**, **2p**, **2r** and **2s**) with electron-donating substituents (–methoxy &–amino) and pyridine ring showed positive results. The anti-tyrosinase effects of biphenyl esters were evaluated at concentrations of 50, 100 and 250 μg/mL. The percentage of inhibition against tyrosinase enzyme of **2i**, **2o**, **2p**, **2r** and **2s** are presented in Fig 5. At the concentration of 250 μg/mL, compounds **2p** (3-amino), **2r** (2-pyridine) and **2s** (3-pyridine) showed strong activities with inhibition percentage of 57.33%, 58.90% and 60.34%, respectively, which are comparable to standard drug, kojic acid (57.22%), with no statistical difference. On the other hand, all five active compounds showed an average inhibition percentage of 50% at 100 μg/mL. All compounds showed weaker effect than kojic acid at 50 μg/mL, except **2s**, which is the best inhibitor in this study, able to inhibit 46.24% of tyrosinase enzyme. In summary, biphenyl compounds which consist of *ortho-*/*meta*-amino group and pyridine ring showed significant response towards tyrosinase enzyme inhibition.

### Docking and structure-activity relationship

**Fig 6:**
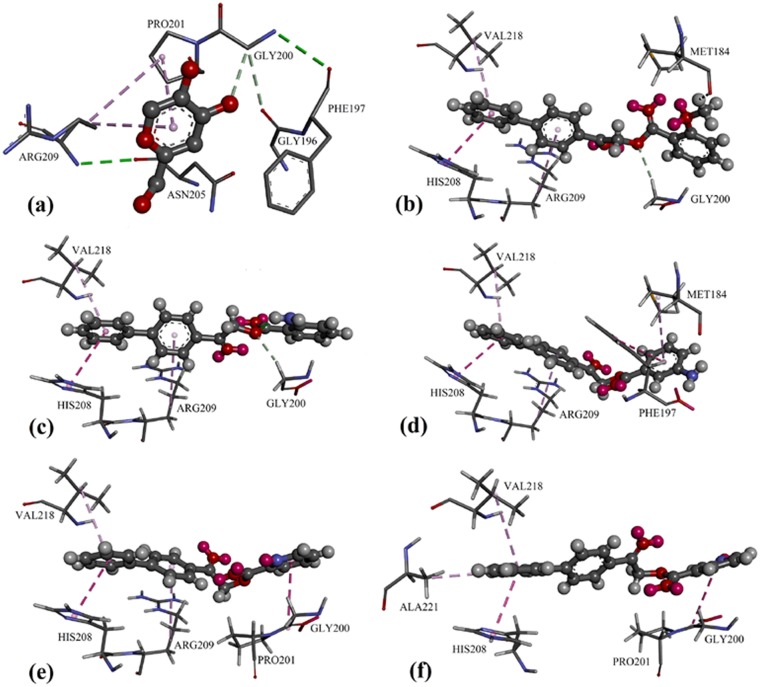
**(a)** Kojic acic binding mode in the crystal structure of TyrBm. Putative binding mode inside the binding gorge of TyrBm of ligands: **(b) 2i**, **(c) 2o, (d) 2p, (e)2r** and **(f) 2s**.

In the protein crystal structure of 3NQ1, inhibitor kojic acid was bound to the active-site entrance of protein TyrBm with hydrogen bond and C–H···π interaction, involving residues Gly200, Arg209 and Pro201 (Fig 6a). The docking models of active compounds **2i**, **2o**, **2p**, **2r** and **2s** are illustrated in Fig 6b and 6f. The first phenyl ring in biphenyl moiety was bound to the copper binding site via C–H···π and π···π interactions with residues His208 and Val218 (additional C–H···π interaction with residue Ala221 for ligand **2s**), while the second phenyl ring in biphenyl moiety was bound to residue Arg209 through C-H···π interaction. For compounds **2i** and **2o**, their carbonyl moiety was bound with residue Gly200 but the substituted phenyl ring failed to bind with other residues at the active-site entrance (except Met184 for compound **2i**) and this leads to a decrease in enzyme inhibition activities. For compound **2p**, its amino benzene moiety was bound with two residues (Met184 and Phe197) *via* C–H···π interaction, thus exhibiting stronger inhibition effects than **2i** and **2o**. Similar to kojic acid, the compounds **2r** and **2s**, which showed the strongest activities, contain electron donating pyridine ring that are able to bind with both Pro201 and Gly200 residues at the active-site entrance. The docking results showed that the compound with biphenyl moiety are able to penetrate the active-site entrance and bind to the copper binding site (Ala221, His208 and Val218) by using the advantage of electron mobilized benzene ring, in succession suggesting that the key for strong tyrosinase inhibition effects are the attached heterocyclic ring which are able to shield the active-site entrance by binding itself with residue Pro201 and Gly200.

## Conclusion

A series of 2-([1,1'-biphenyl]-4-yl)-2-oxoethyl benzoates, **2**(**a**-**q**), and 2-([1,1'-biphenyl]-4-yl)-2-oxoethyl pyridinecarboxylate, **2**(**r**-**s**), were synthesized and characterized by FTIR, ^1^H and ^13^C NMR spectroscopic analysis, its 3D structure was further confirmed by single-crystal X-ray diffraction studies. Introduction of biphenyl moiety into the synthesis of 2-oxopropyl benzoate derivatives produced crystal structure with higher structural occupancy by augment of the weak π···π and C–H···π interactions. Five compounds showed tyrosine inhibitory activities, while at 250 μg/mL, **2p**, **2r**, and **2s**exhibited high inhibition comparable to the standard drug, kojic acid. In addition, the computational molecular docking results suggested pyridine ring has a better binding affinity toward TyrBm. Thus, further modification of biphenyl compounds substituted with heterocyclic ring can potentially produce promising anti-tyrosinase agents for clinical use in the future.

## Supporting information

S1 Dataset (DOCX)
