# Supplementary material for: Novel biphenyl ester derivatives as tyrosinase inhibitors: Synthesis, crystallographic, spectral analysis and molecular docking studies
Source: PLoS One. 2017 Feb 27;12(2):e0170117. doi: 10.1371/journal.pone.0170117 (PMC5328250; doi:10.1371/journal.pone.0170117)
Supplement: S1 Dataset — (DOCX) [file pone.0170117.s001.docx]

***Supporting data***

**Novel Biphenyl Ester Derivatives as Tyrosinase Inhibitors: Synthesis, Crystallographic, Spectral Analysis and Molecular Docking Studies**

**Huey Chong Kwong^1^, C. S. Chidan Kumar^2^**^#^**, Siau Hui Mah^3^, Tze Shyang Chia^4^, Ching Kheng Quah^4^*****, Zi Han Loh^3^, Siddegowda Chandraju^5^ and Gin Keat Lim^1^**

^1^School of Chemical Sciences, Universiti Sains Malaysia, Penang 11800 USM, Malaysia

^2^Department of Engineering Chemistry, Vidya Vikas Institute of Engineering & Technology, Visvesvaraya Technological University, Alanahalli, Mysuru 570028, Karnataka, India

^3^School of Biosciences, Taylor’s University, Lakeside Campus, Subang Jaya 47500, Selangor,

Malaysia

^4^X-ray Crystallography Unit, School of Physics, Universiti Sains Malaysia, Penang 11800 USM, Malaysia

^5^Department of Sugar Technology & Chemistry, Sir M.V. PG Center, University of Mysore, Tubinakere 571402, India.

*Corresponding author email: [ckquah@usm.my](mailto:ckquah@usm.my)

^#^Co-corresponding author email: [chidankumar@gmail.com](mailto:chidankumar@gmail.com)

Tel.: +604 653 3888;Ext. 3690; Fax: +6046579150.

Contents Page

[1.0 ^1^H NMR, ^13^C NMR and FTIR spectra 3](#_Toc469491585)

[1.1 2-([1,1'-biphenyl]-4-yl)-2-oxoethyl benzoate (**2a**) 3](#_Toc469491586)

[1.2 2-([1,1'-biphenyl]-4-yl)-2-oxoethyl 2-chlorobenzoate (**2b**) 5](#_Toc469491587)

[1.3 2-([1,1'-biphenyl]-4-yl)-2-oxoethyl 3-chlorobenzoate (**2c**) 7](#_Toc469491588)

[1.4 2-([1,1'-biphenyl]-4-yl)-2-oxoethyl 4-chlorobenzoate (**2d**) 9](#_Toc469491589)

[1.5 2-([1,1'-biphenyl]-4-yl)-2-oxoethyl 2,4-dichlorobenzoate (**2e**) 11](#_Toc469491590)

[1.6 2-([1,1'-biphenyl]-4-yl)-2-oxoethyl 2-methylbenzoate (**2f**) 13](#_Toc469491591)

[1.7 2-([1,1'-biphenyl]-4-yl)-2-oxoethyl 3-methylbenzoate (**2g**) 15](#_Toc469491592)

[1.8 2-([1,1'-biphenyl]-4-yl)-2-oxoethyl 4-methylbenzoate (**2h**) 17](#_Toc469491593)

[1.9 2-([1,1'-biphenyl]-4-yl)-2-oxoethyl 2-methoxybenzoate (**2i**) 19](#_Toc469491594)

[1.10 2-([1,1'-biphenyl]-4-yl)-2-oxoethyl 3-methoxybenzoate (**2j**) 21](#_Toc469491595)

[1.11 2-([1,1'-biphenyl]-4-yl)-2-oxoethyl 4-methoxybenzoate (**2k**) 23](#_Toc469491596)

[1.12 2-([1,1'-biphenyl]-4-yl)-2-oxoethyl 2-nitrobenzoate (**2l**) 25](#_Toc469491597)

[1.13 2-([1,1'-biphenyl]-4-yl)-2-oxoethyl 3-nitrobenzoate (**2m**) 27](#_Toc469491598)

[1.14 2-([1,1'-biphenyl]-4-yl)-2-oxoethyl 4-nitrobenzoate (**2n**) 29](#_Toc469491599)

[1.15 2-([1,1'-biphenyl]-4-yl)-2-oxoethyl 2-aminobenzoate (**2o**) 31](#_Toc469491600)

[1.16 2-([1,1'-biphenyl]-4-yl)-2-oxoethyl 3-aminobenzoate (**2p**) 33](#_Toc469491601)

[1.17 2-([1,1'-biphenyl]-4-yl)-2-oxoethyl 4-aminobenzoate (**2q**) 35](#_Toc469491602)

[1.18 2-([1,1'-biphenyl]-4-yl)-2-oxoethyl picolinate (**2r**) 37](#_Toc469491603)

[1.19 2-([1,1'-biphenyl]-4-yl)-2-oxoethyl nicotinate (**2s**) 39](#_Toc469491604)

[2.0 Single crystal X-ray crystallography data 41](#_Toc469491605)

[2.1 Refined site occupancy 41](#_Toc469491606)

[2.2 Crystal data and parameters 42](#_Toc469491607)

[2.3 Ortep diagram and atom numbering 44](#_Toc469491608)

[2.4 Supramolecular feature 46](#_Toc469491609)

[2.5 Hydrogen-bond geometry 56](#_Toc469491610)

[3.0 Anti-tyrosinase Assay 58](#_Toc469491611)

[3.1 Percentage of inhibition 58](#_Toc469491612)

# 1.0 ^1^H NMR, ^13^C NMR and FTIR spectra

## 1.1 2-([1,1'-biphenyl]-4-yl)-2-oxoethyl benzoate (**2a**)

**
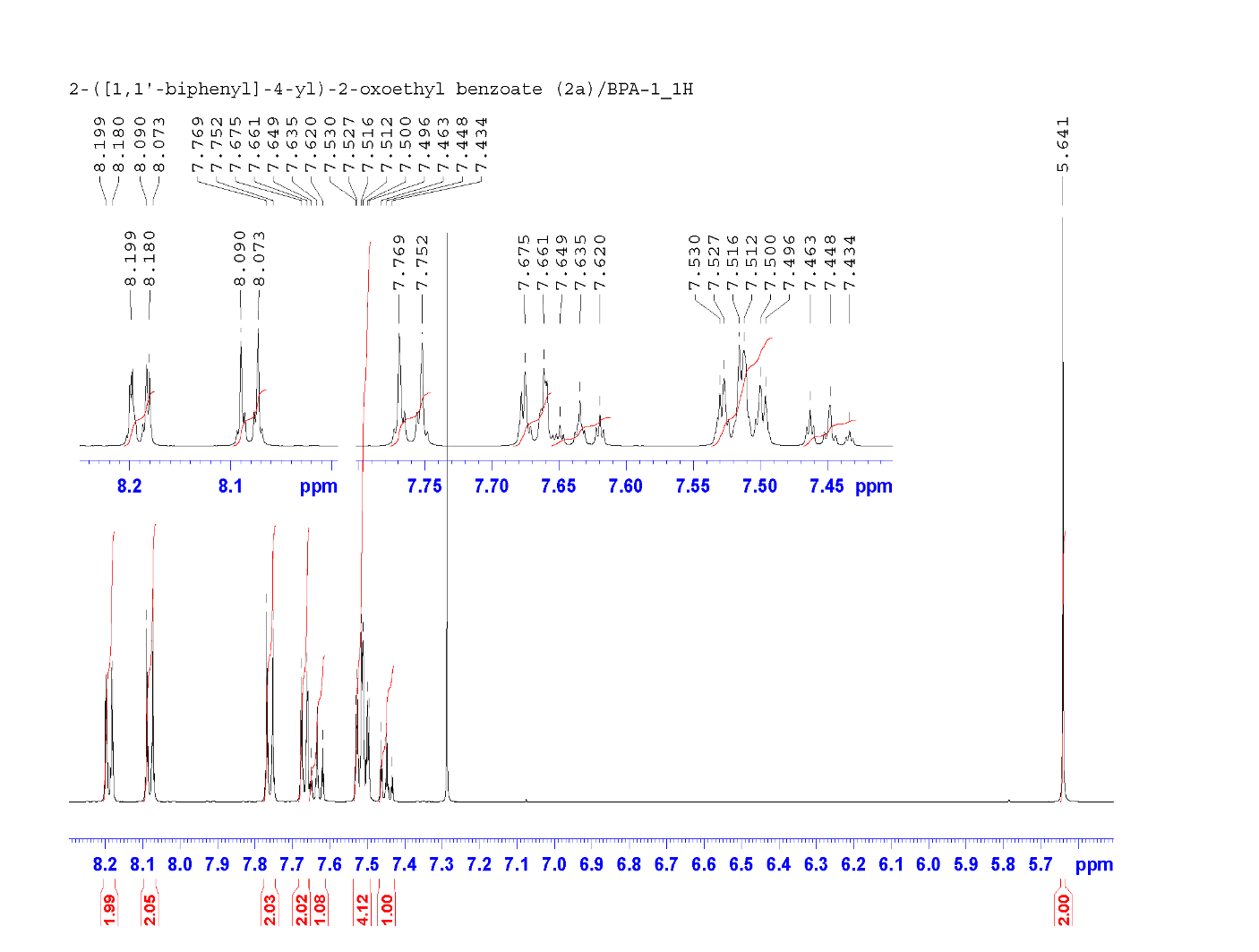
**

**
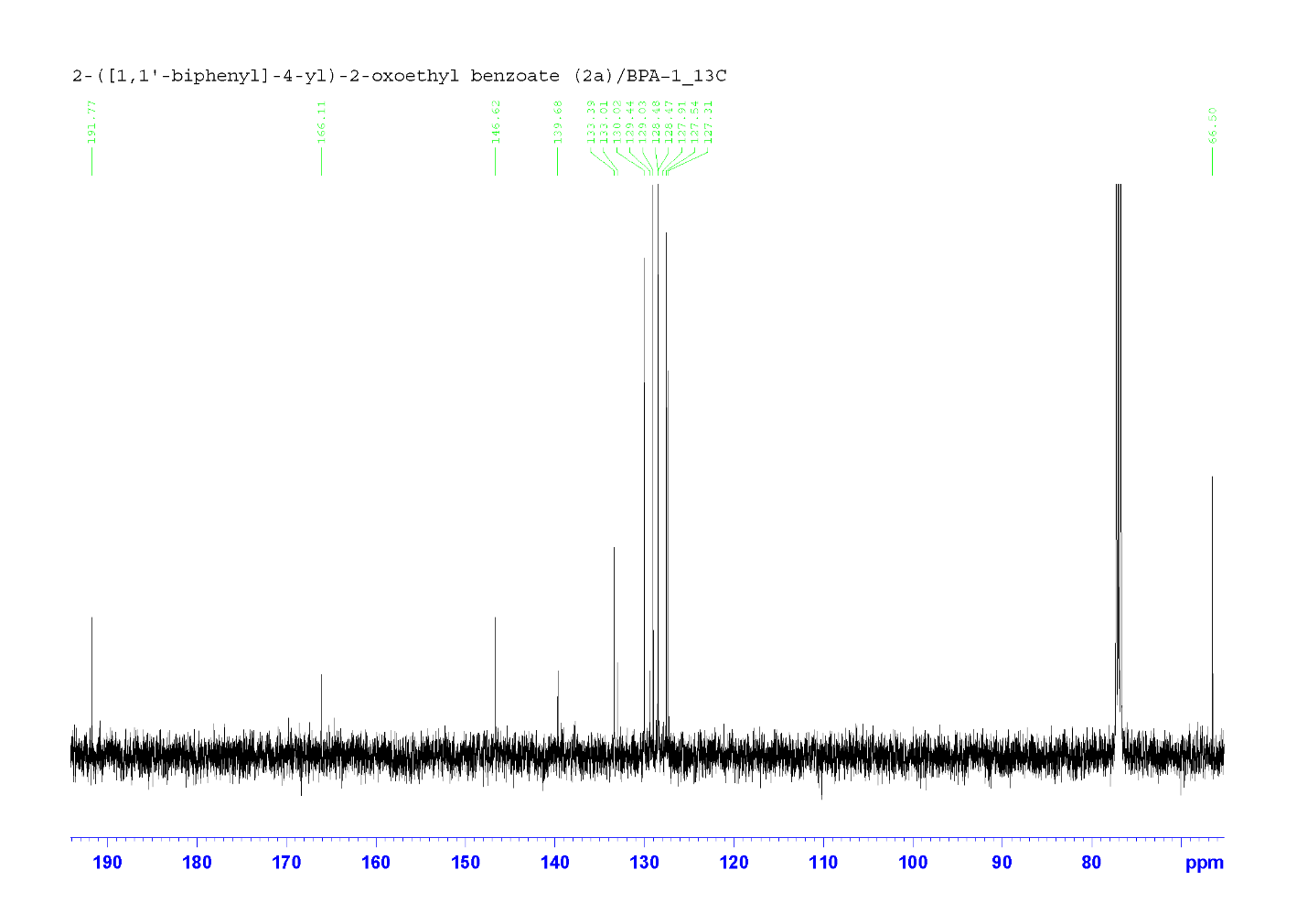
**

*2-([1,1'-biphenyl]-4-yl)-2-oxoethyl benzoate* (**2a**)/BPA_1_FTIR

BPA_1

Name

Sample 012 By Administrator Date Thursday, April 30 2015

Description

4000

600

3500

3000

2500

2000

1500

1000

95

28

30

35

40

45

50

55

60

65

70

75

80

85

90

cm-1

%T

3062.6, Ar C-H, v

2936.2, C-H, v

1717.6, C=O, v

1696.1, C=O, v

1599.3, Ar C-C, v

1451.3, Ar C-C, v

1276.5, C-O, v

1233.5, C-O, v

1123.2, C-O, v

## 1.2 2-([1,1'-biphenyl]-4-yl)-2-oxoethyl 2-chlorobenzoate (**2b**)

**
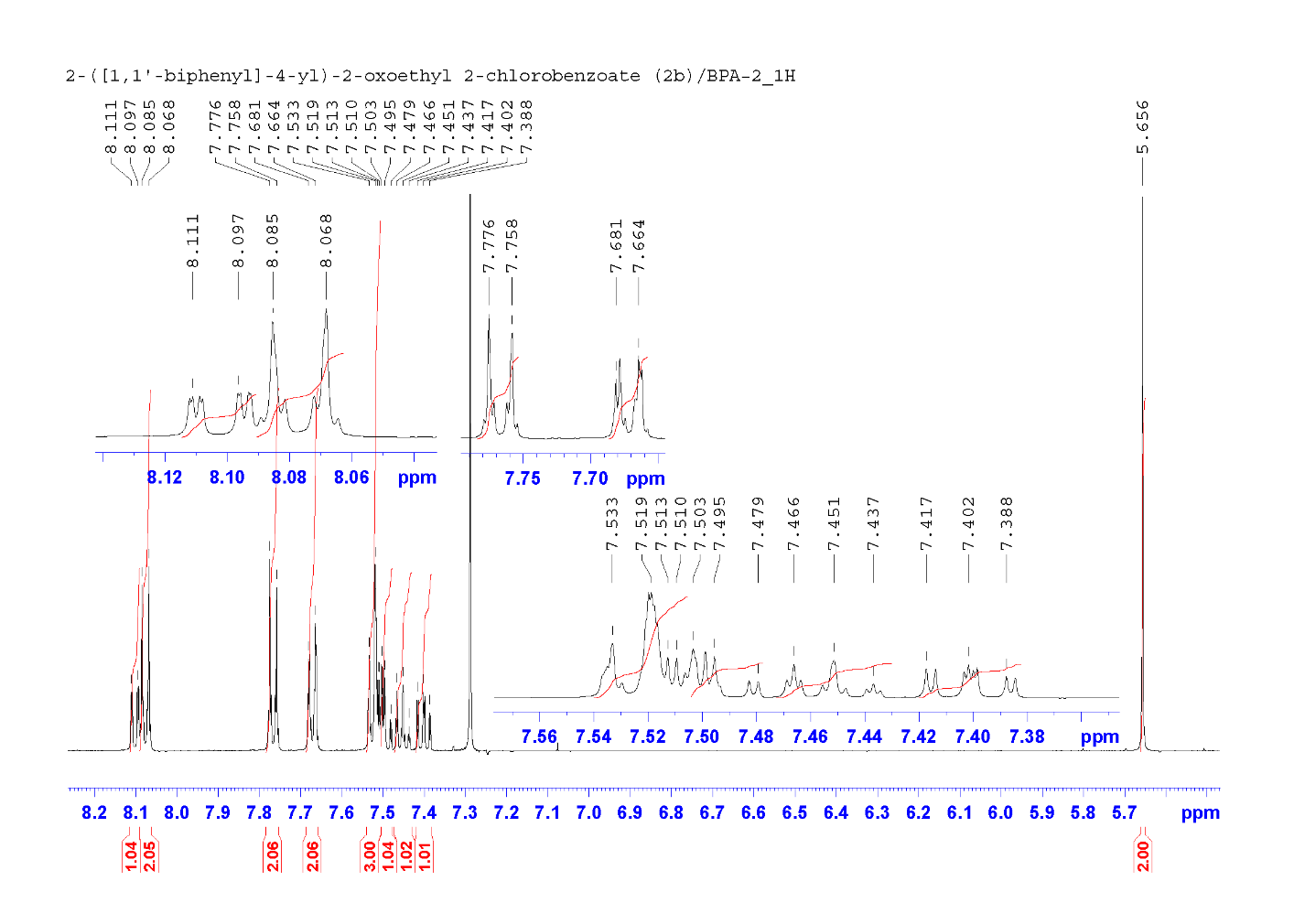
**

**
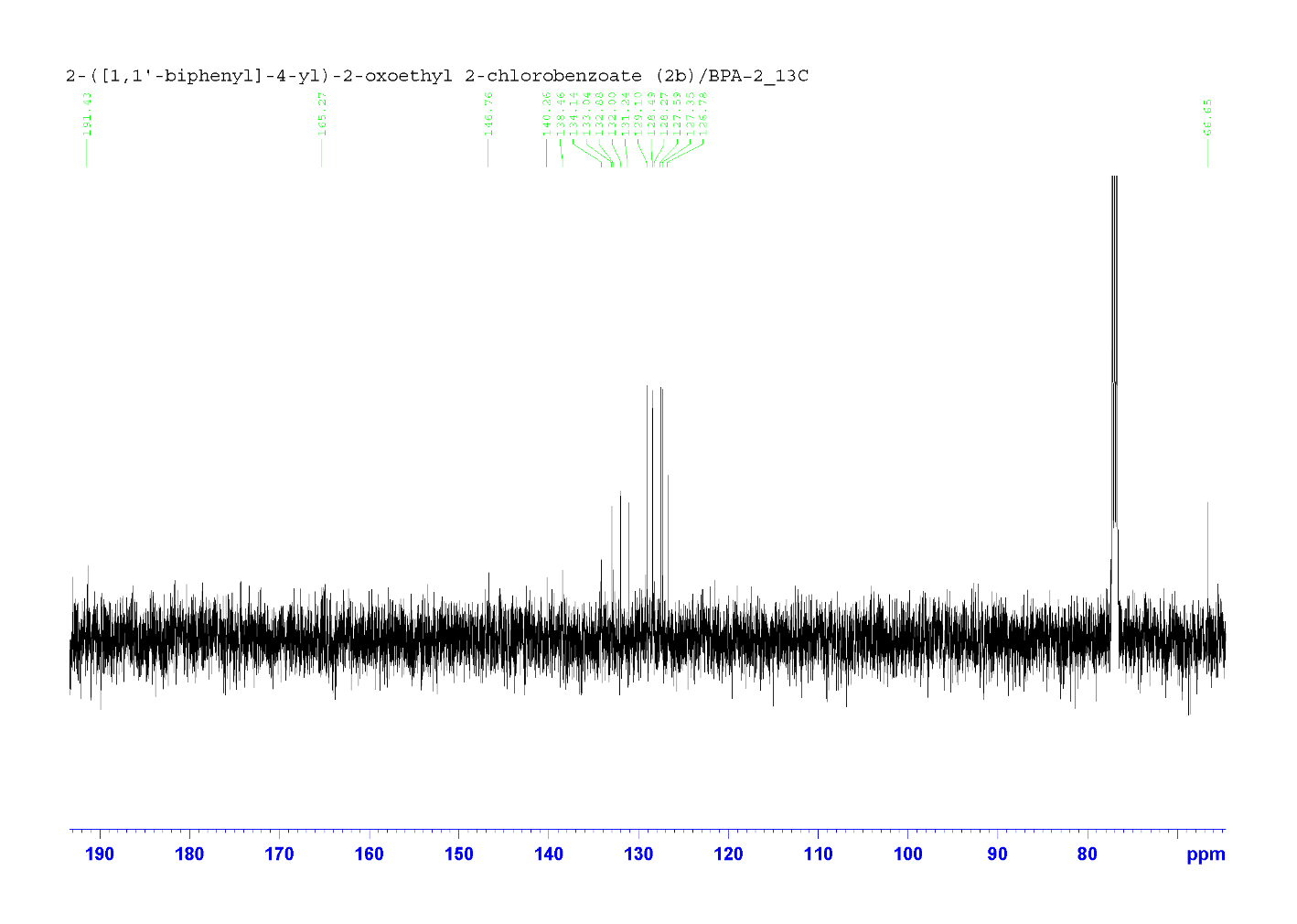
**

*2-([1,1'-biphenyl]-4-yl)-2-oxoethyl 2-chlorobenzoate* (**2b**)/BPA_2_FTIR

## 1.3 2-([1,1'-biphenyl]-4-yl)-2-oxoethyl 3-chlorobenzoate (**2c**)

**
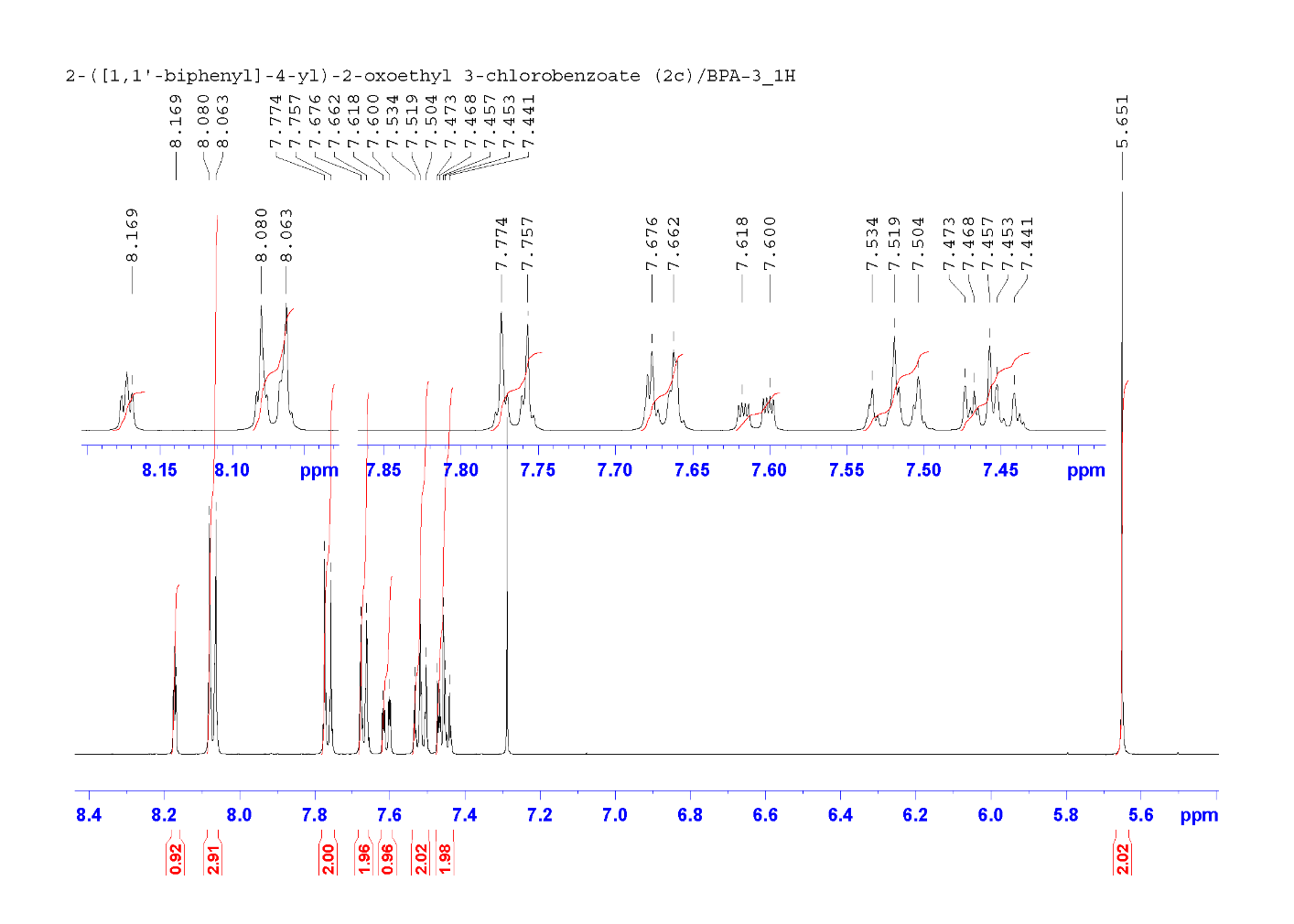
**

**
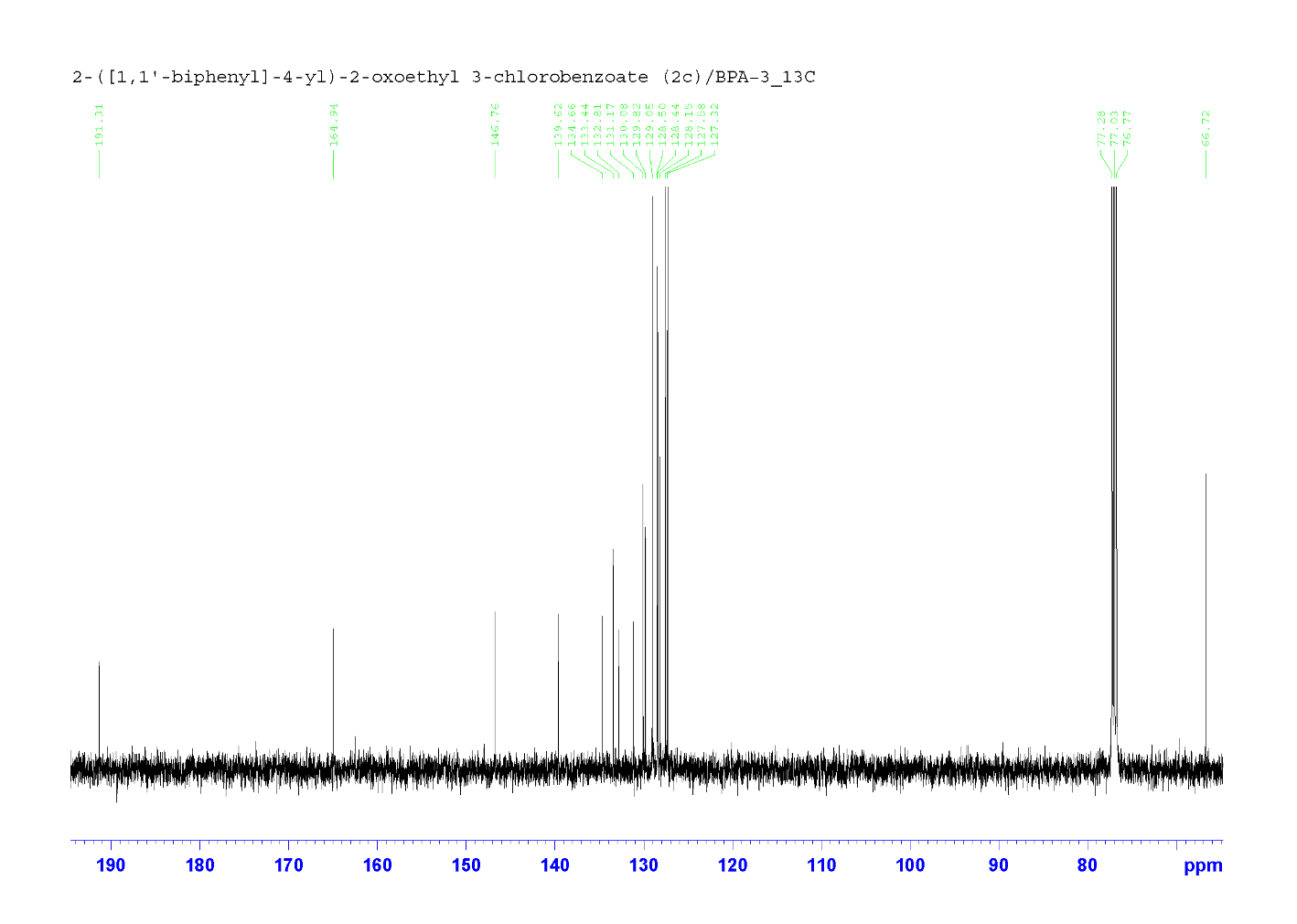
**

*2-([1,1'-biphenyl]-4-yl)-2-oxoethyl 3-chlorobenzoate* (**2c**)/BPA_3_FTIR

## 1.4 2-([1,1'-biphenyl]-4-yl)-2-oxoethyl 4-chlorobenzoate (**2d**)

**
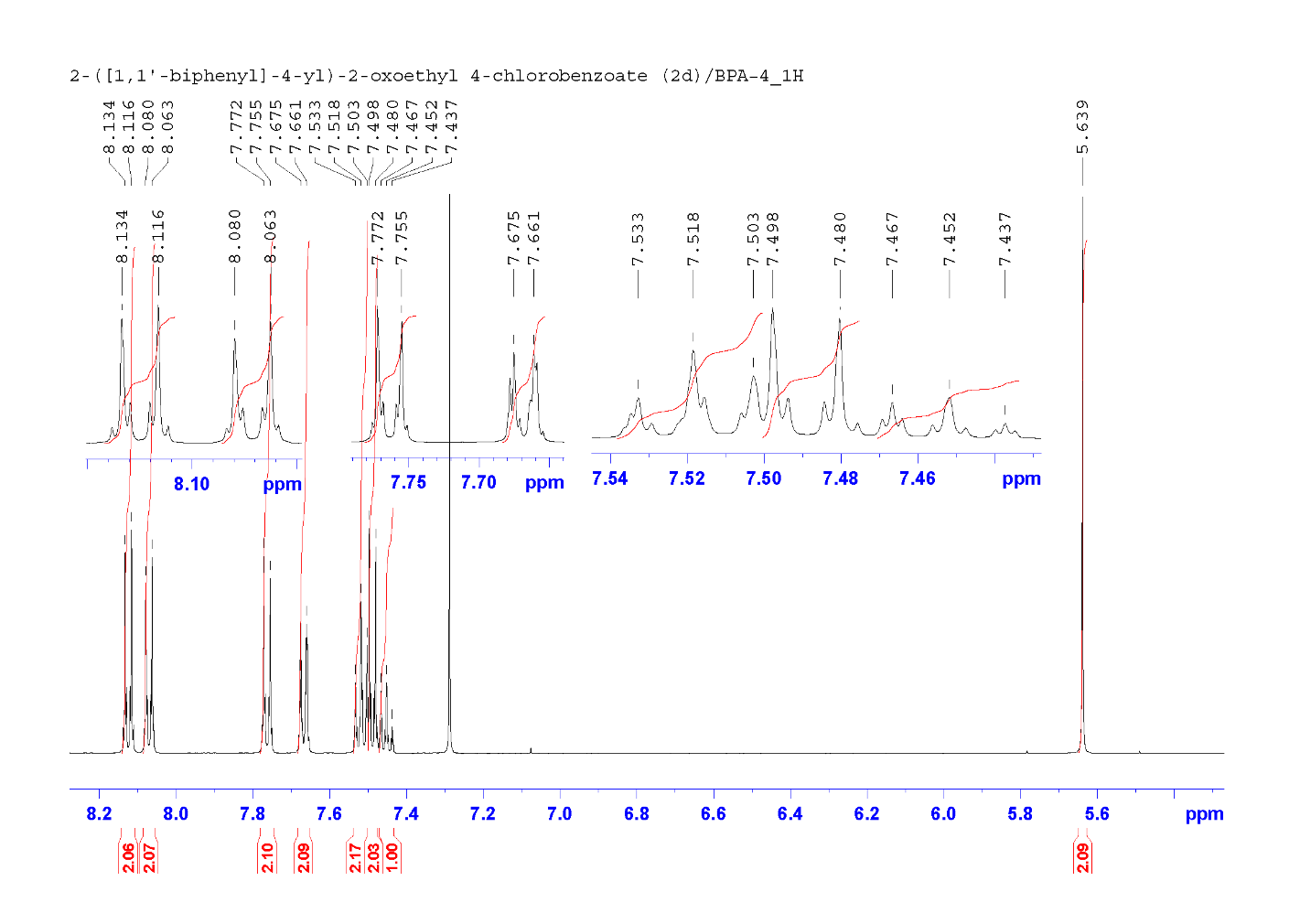
**

**
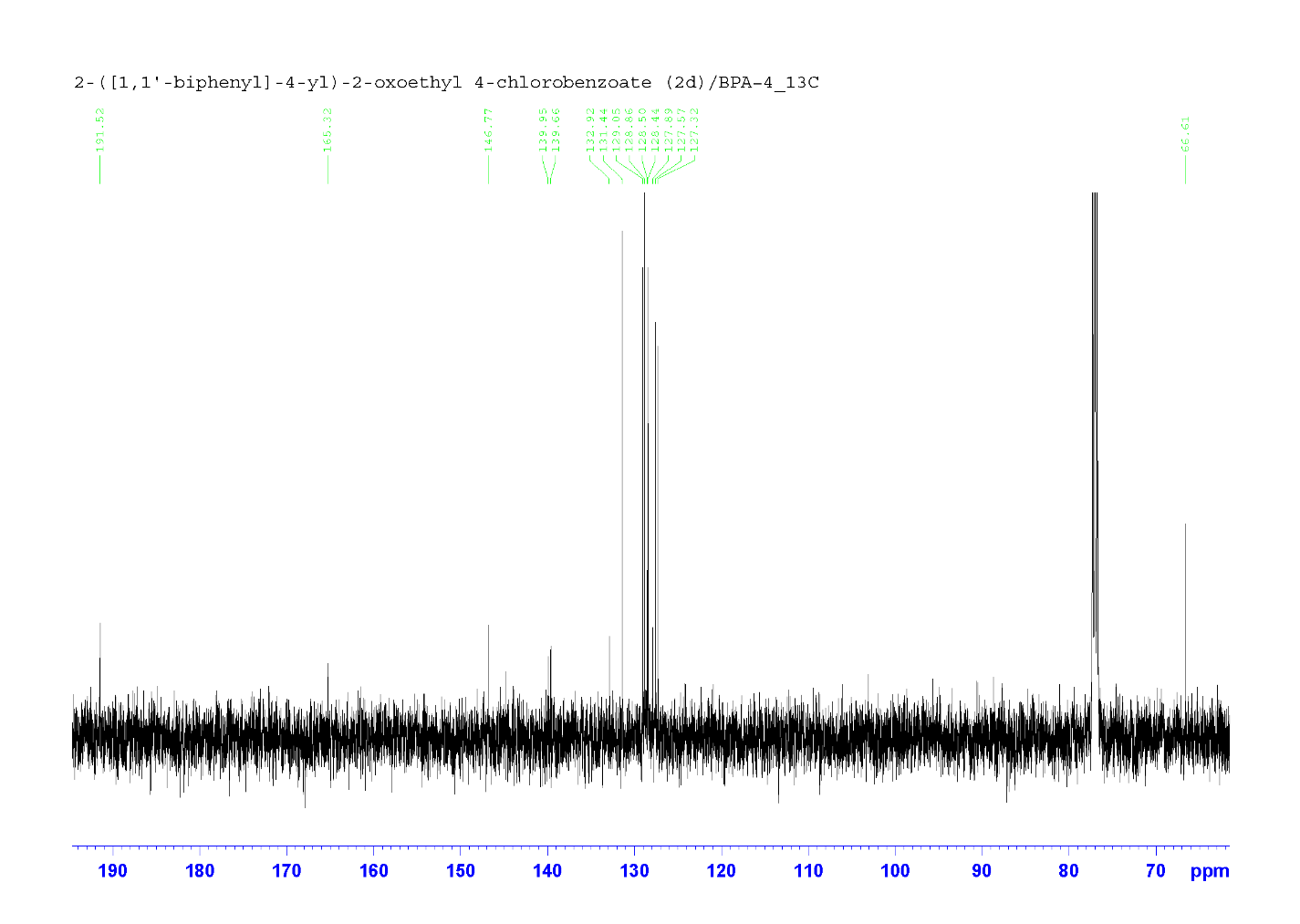
**

*2-([1,1'-biphenyl]-4-yl)-2-oxoethyl 4-chlorobenzoate* (**2d**)/BPA_4_FTIR

## 1.5 2-([1,1'-biphenyl]-4-yl)-2-oxoethyl 2,4-dichlorobenzoate (**2e**)

**
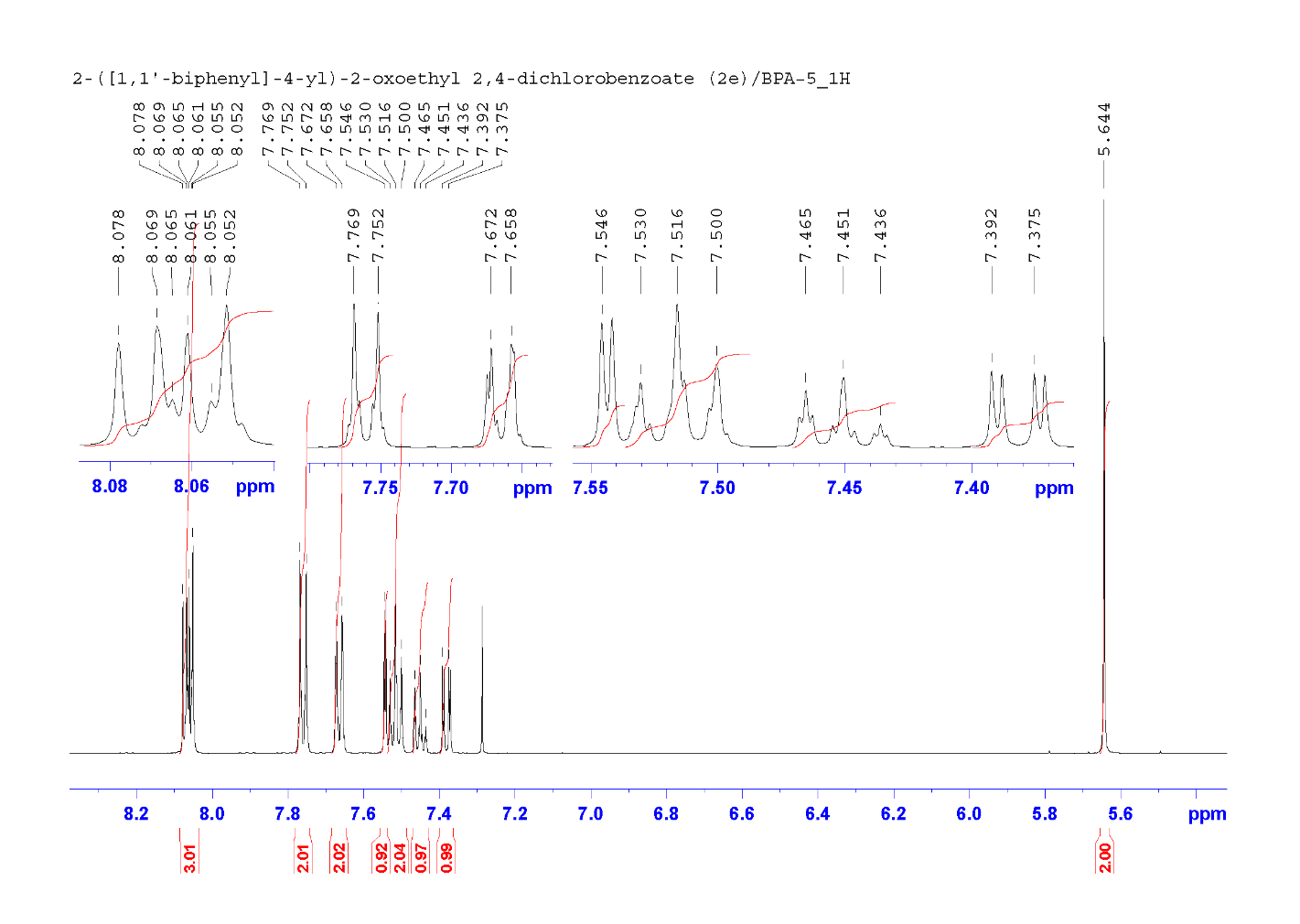
**

**
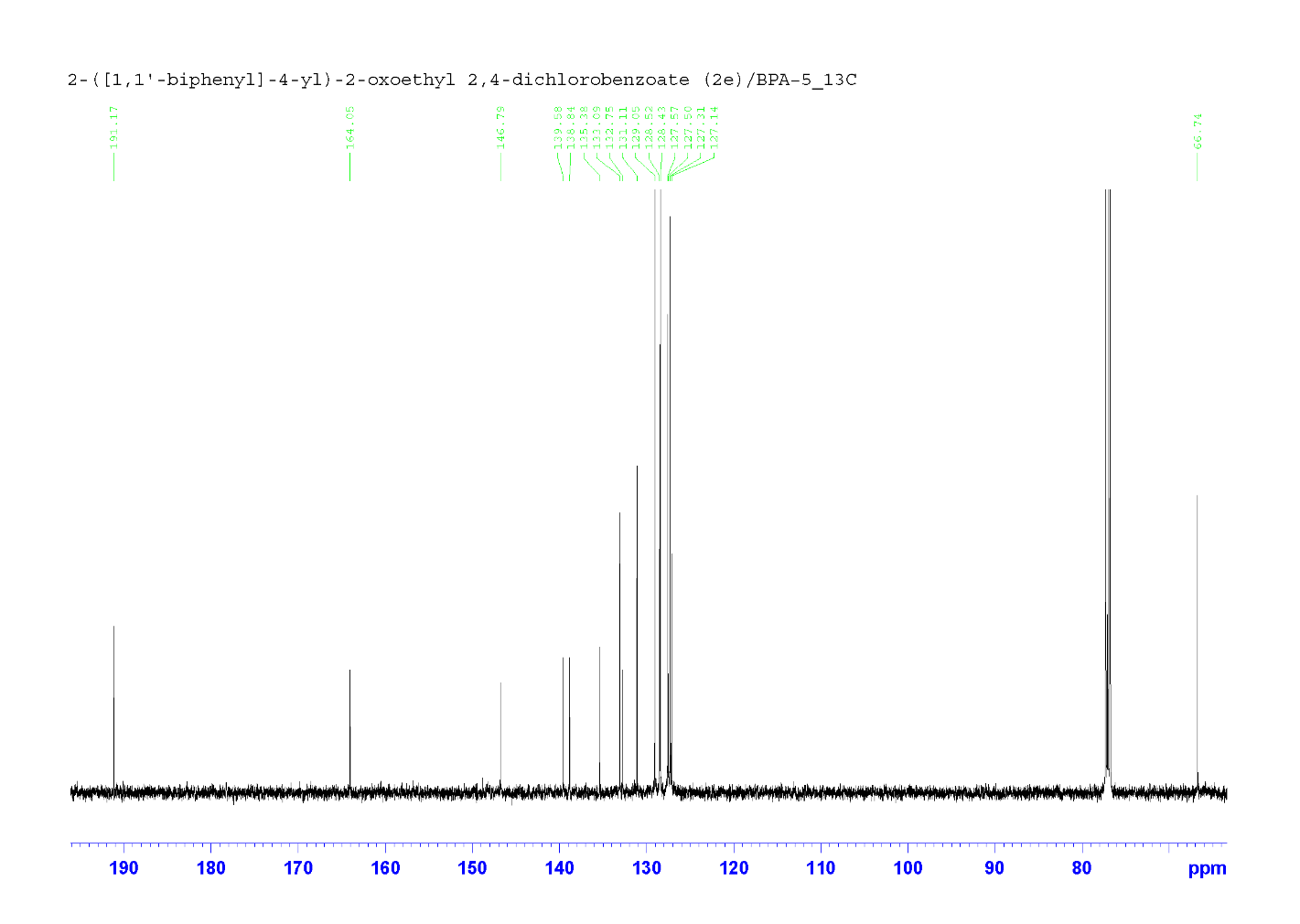
**

*2-([1,1'-biphenyl]-4-yl)-2-oxoethyl 2,4-dichlorobenzoate* (**2e**)/BPA_5_FTIR

BPA_5

Name

Diphenyl oxalate

Description

4000

650

3500

3000

2500

2000

1500

1000

93

7

10

20

30

40

50

60

70

80

90

cm-1

%T

3092.3, Ar C-H, v

2933.2, C-H, v

1735.3, C=O, v

1692.9, C=O, v

1602.8, Ar C-C, v

1414.6, Ar C-C, v

1229, C-O, v

1133.7, C-O, v

1104.5, C-O, v

762.64, C-Cl, v

## 1.6 2-([1,1'-biphenyl]-4-yl)-2-oxoethyl 2-methylbenzoate (**2f**)

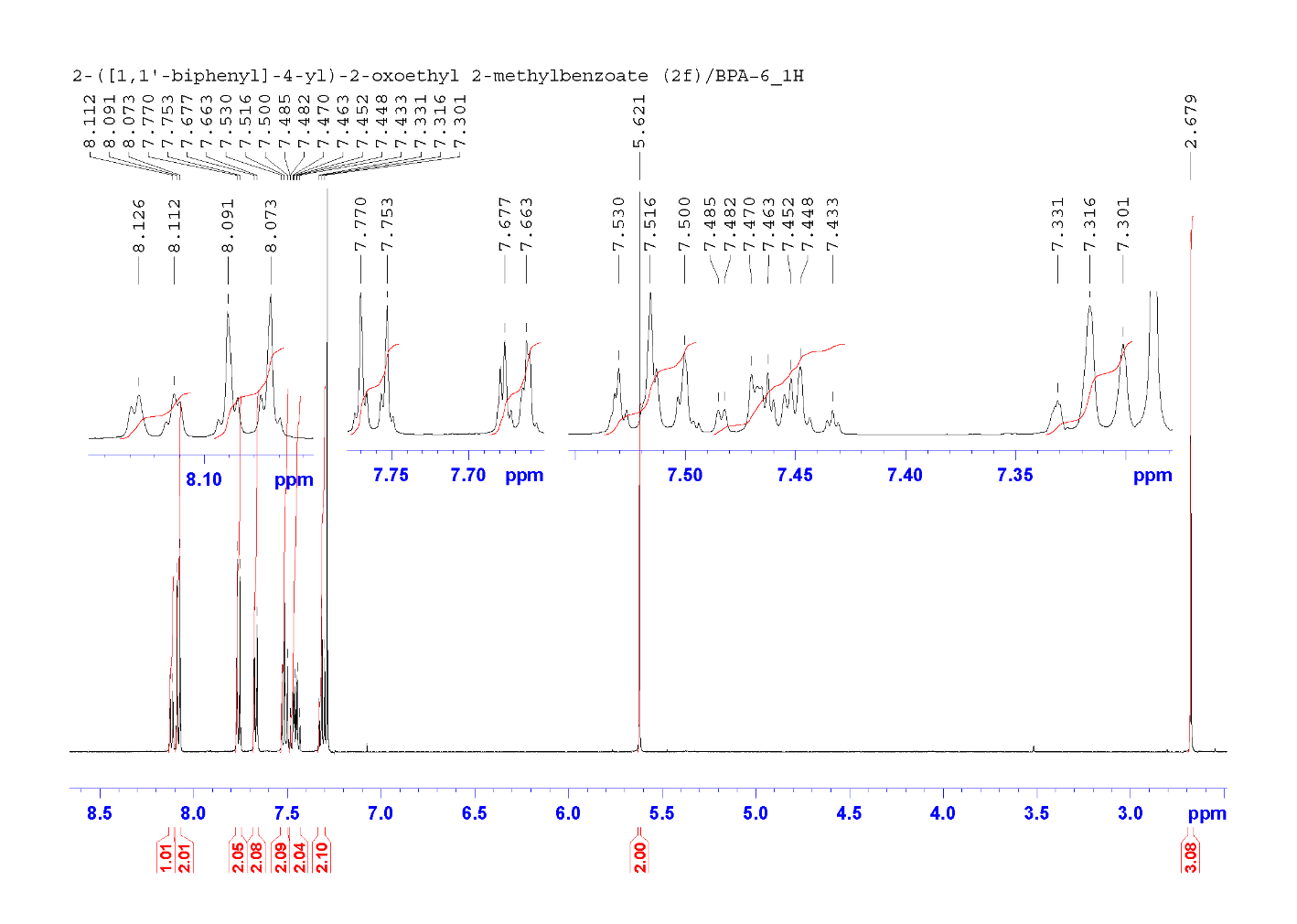


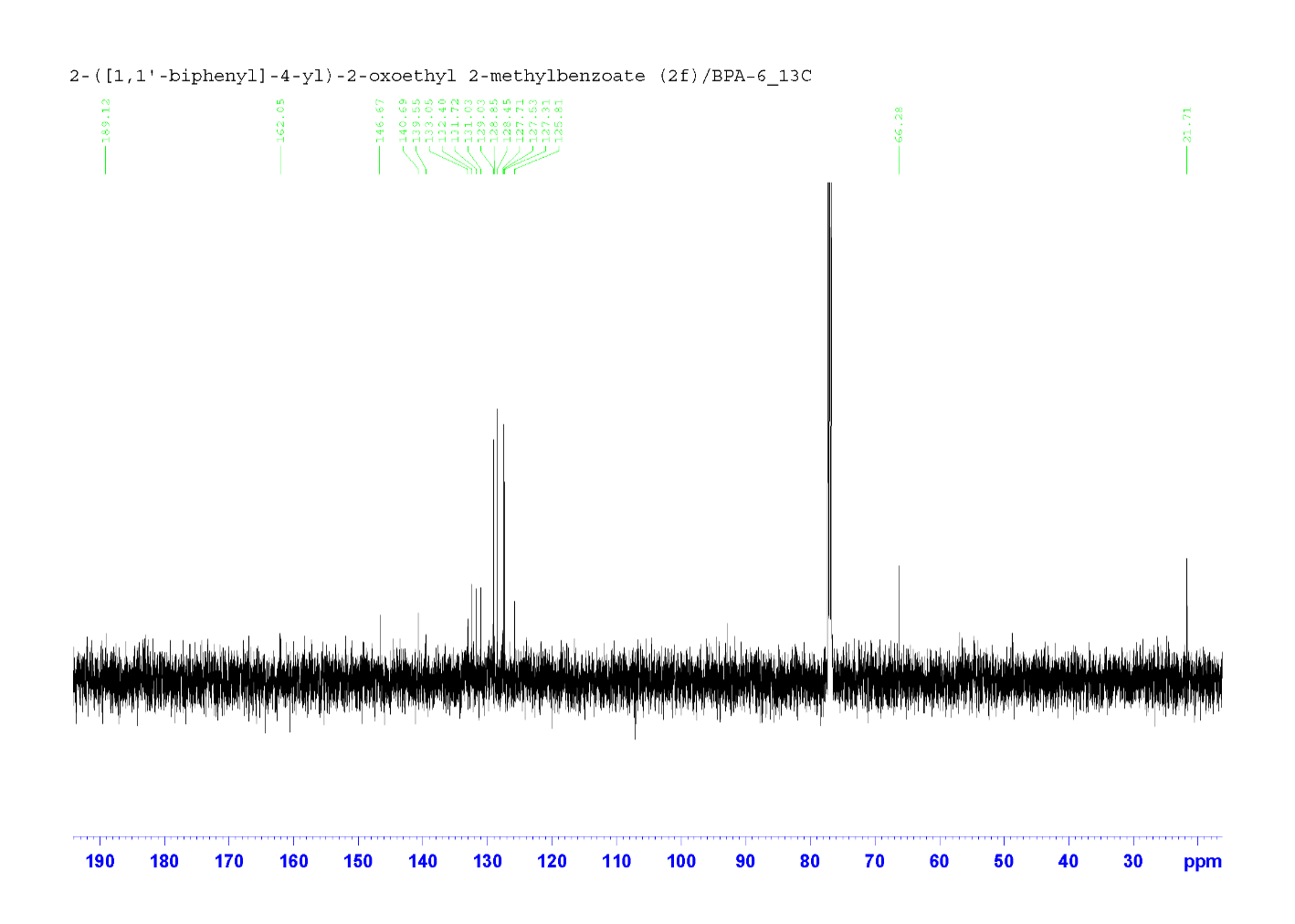


*2-([1,1'-biphenyl]-4-yl)-2-oxoethyl 2-methylbenzoate* (**2f**)/BPA_6_FTIR

## 1.7 2-([1,1'-biphenyl]-4-yl)-2-oxoethyl 3-methylbenzoate (**2g**)

**
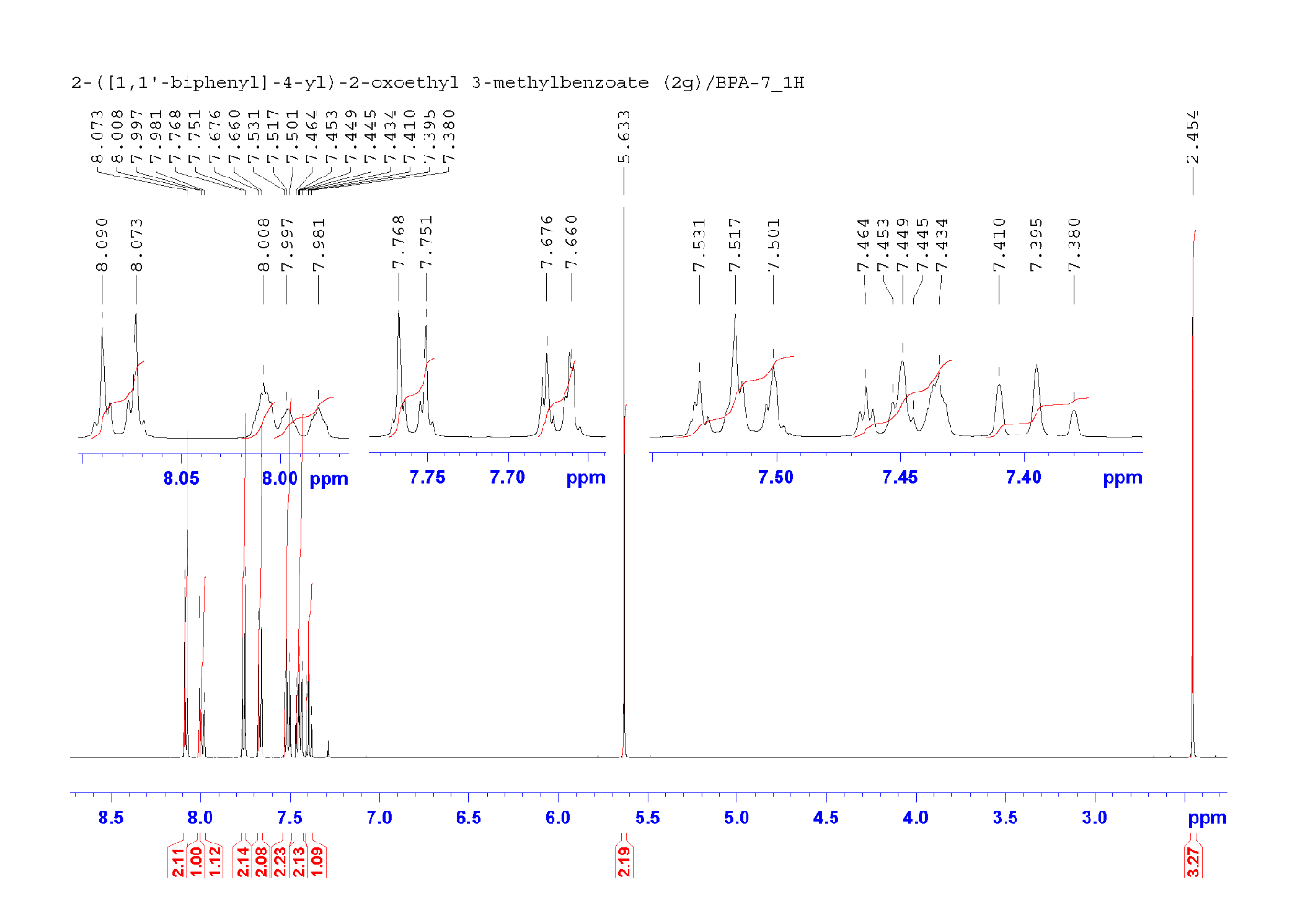
**

**
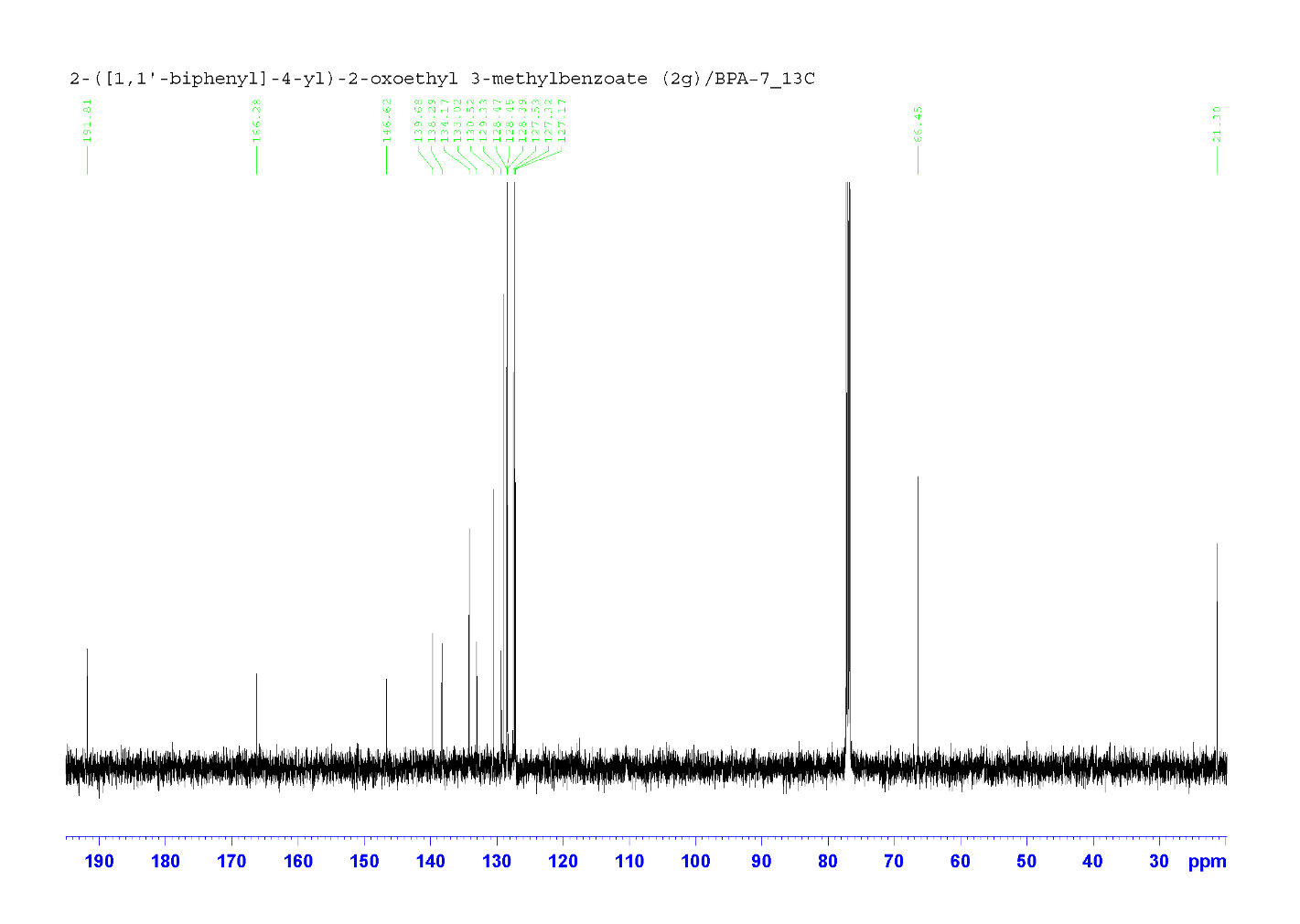
**

*2-([1,1'-biphenyl]-4-yl)-2-oxoethyl 3-methylbenzoate* (**2g**)/BPA_7_FTIR

## 1.8 2-([1,1'-biphenyl]-4-yl)-2-oxoethyl 4-methylbenzoate (**2h**)

**
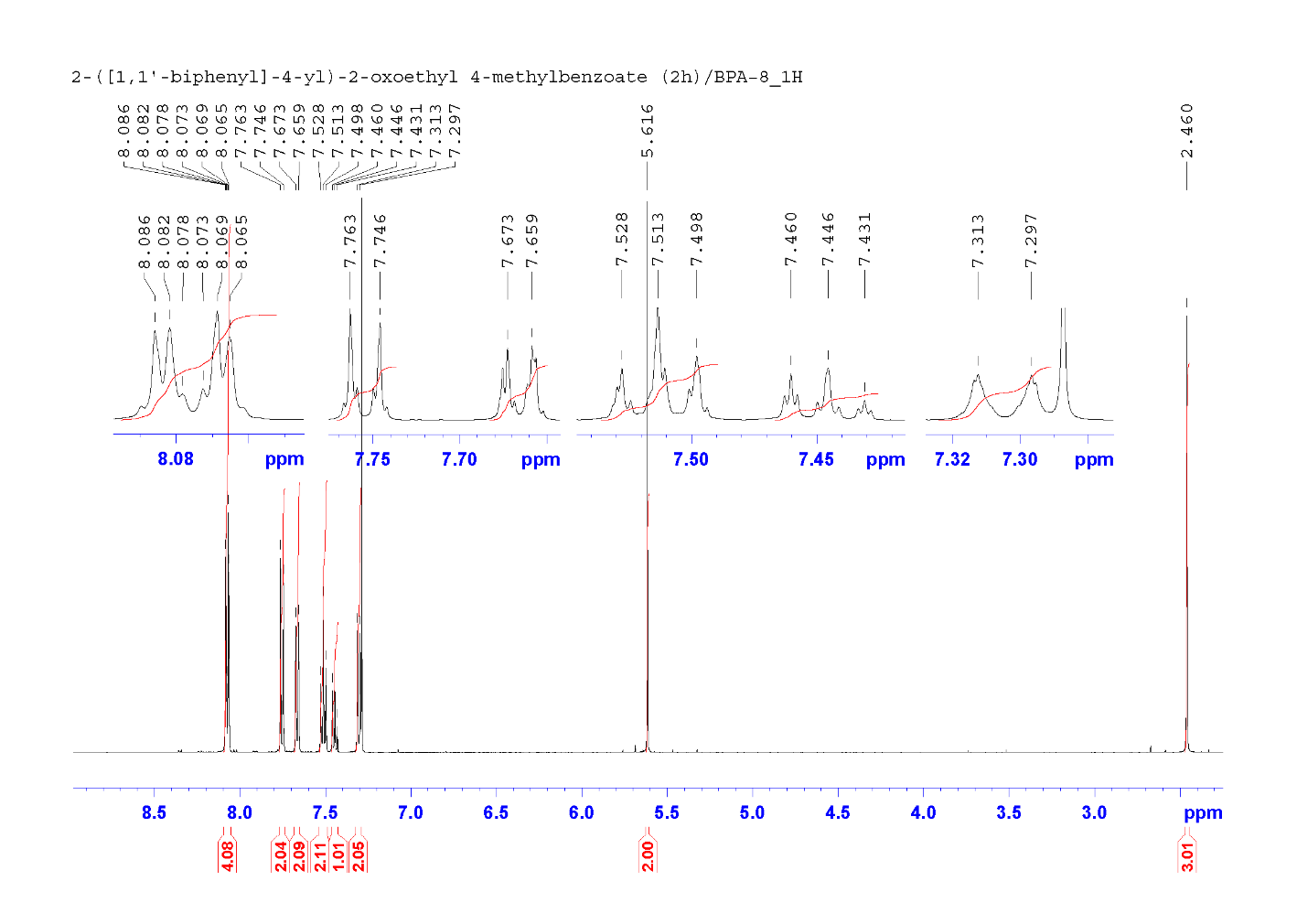
**

**
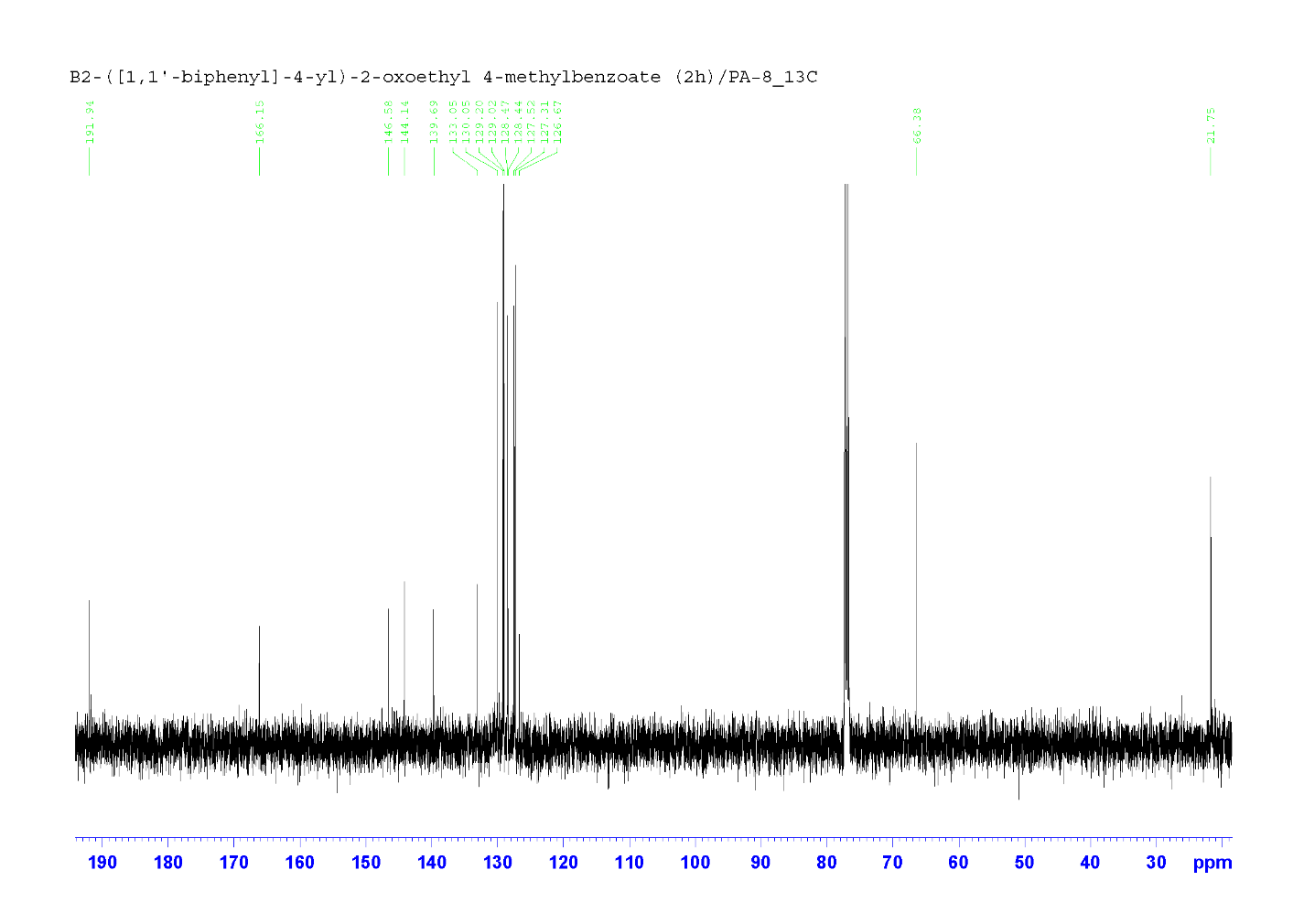
**

*2-([1,1'-biphenyl]-4-yl)-2-oxoethyl 4-methylbenzoate* (**2h**)/BPA_8_FTIR

## 1.9 2-([1,1'-biphenyl]-4-yl)-2-oxoethyl 2-methoxybenzoate (**2i**)

**
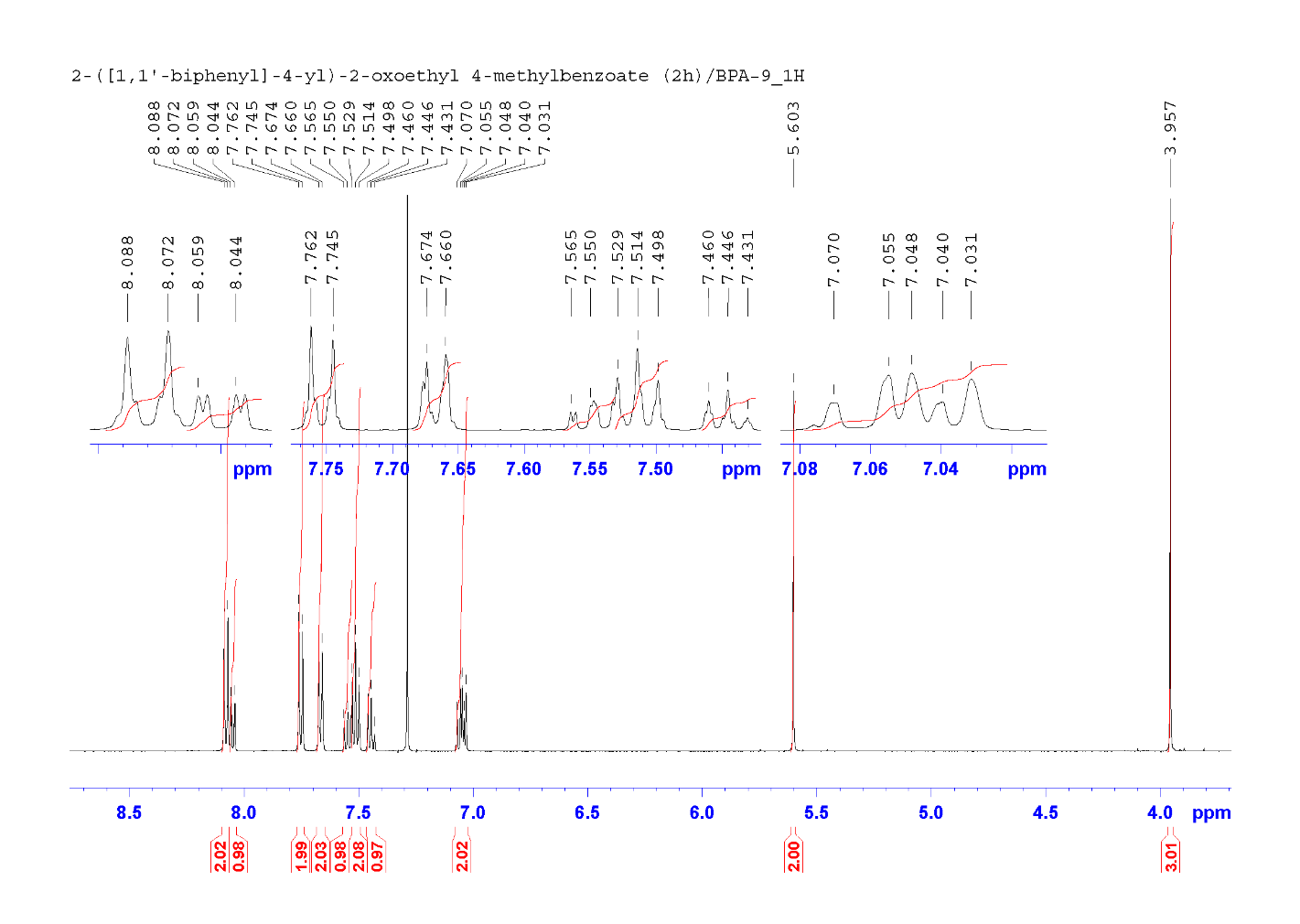
**

**
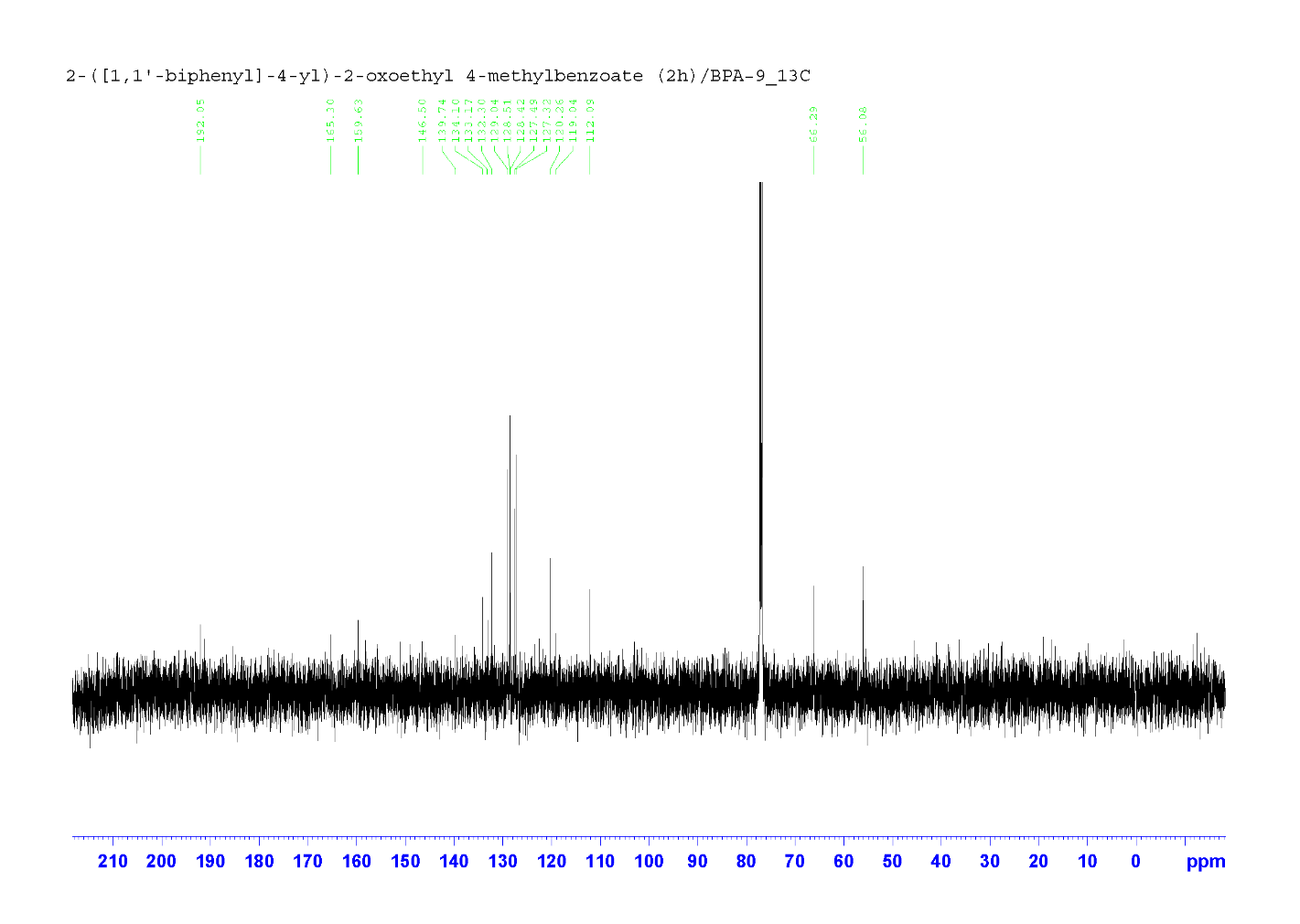
**

*2-([1,1'-biphenyl]-4-yl)-2-oxoethyl 2-methoxybenzoate*(**2i**)/BPA_9_FTIR

## 1.10 2-([1,1'-biphenyl]-4-yl)-2-oxoethyl 3-methoxybenzoate (**2j**)

**
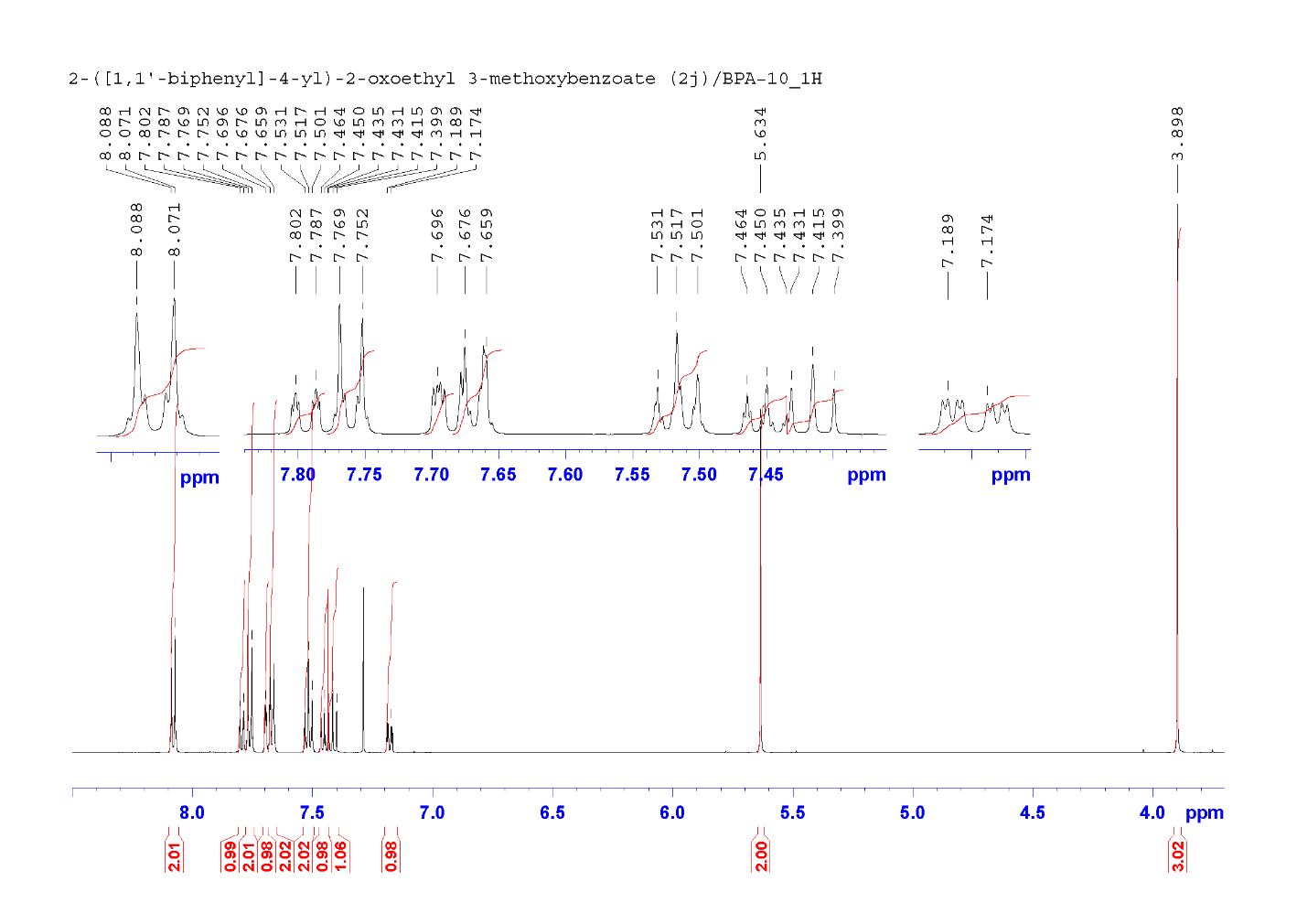
**

**
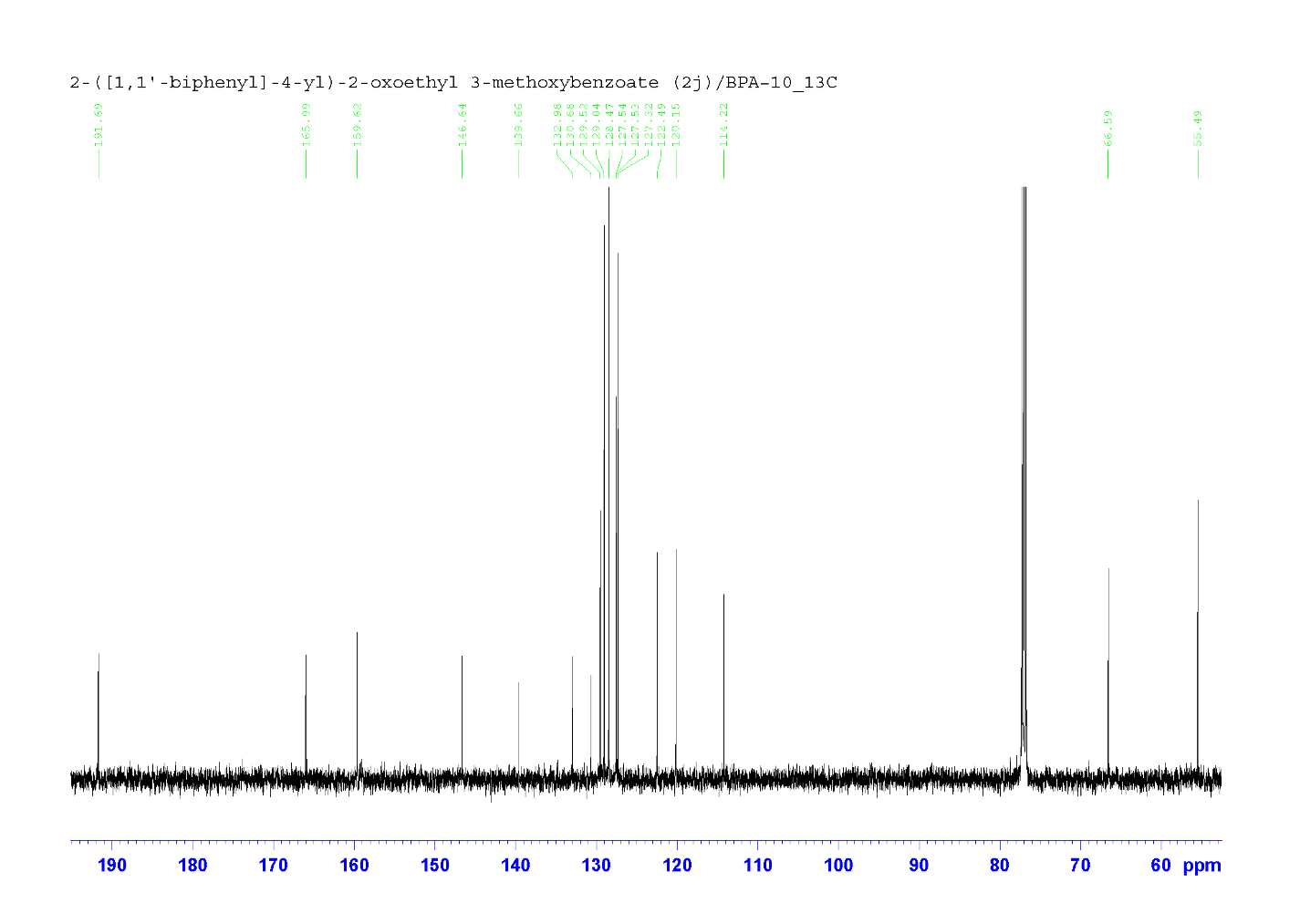
**

*2-([1,1'-biphenyl]-4-yl)-2-oxoethyl 3-methoxybenzoate* (**2j**)/BPA_10_FTIR

## 1.11 2-([1,1'-biphenyl]-4-yl)-2-oxoethyl 4-methoxybenzoate (**2k**)

**
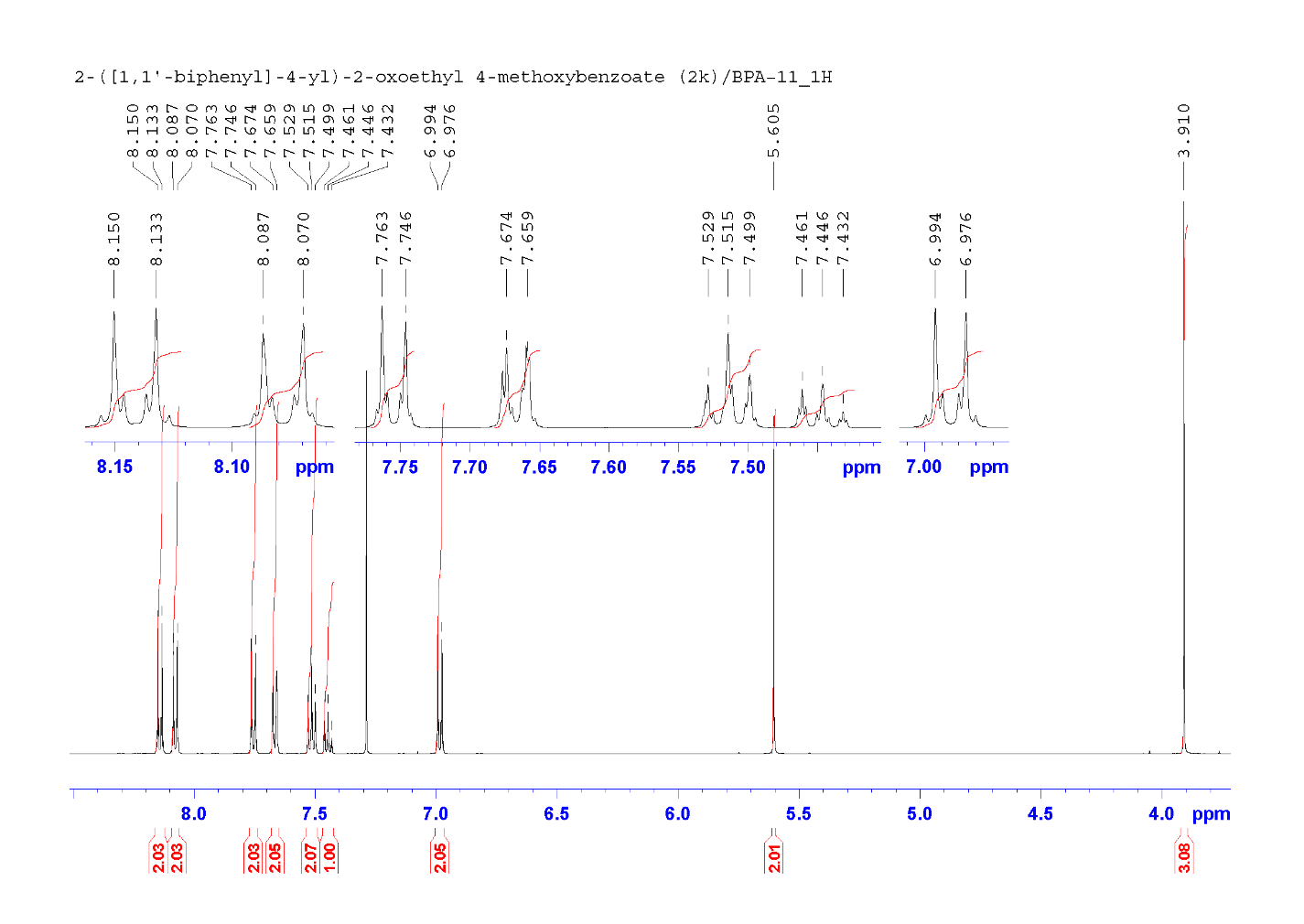
**

**
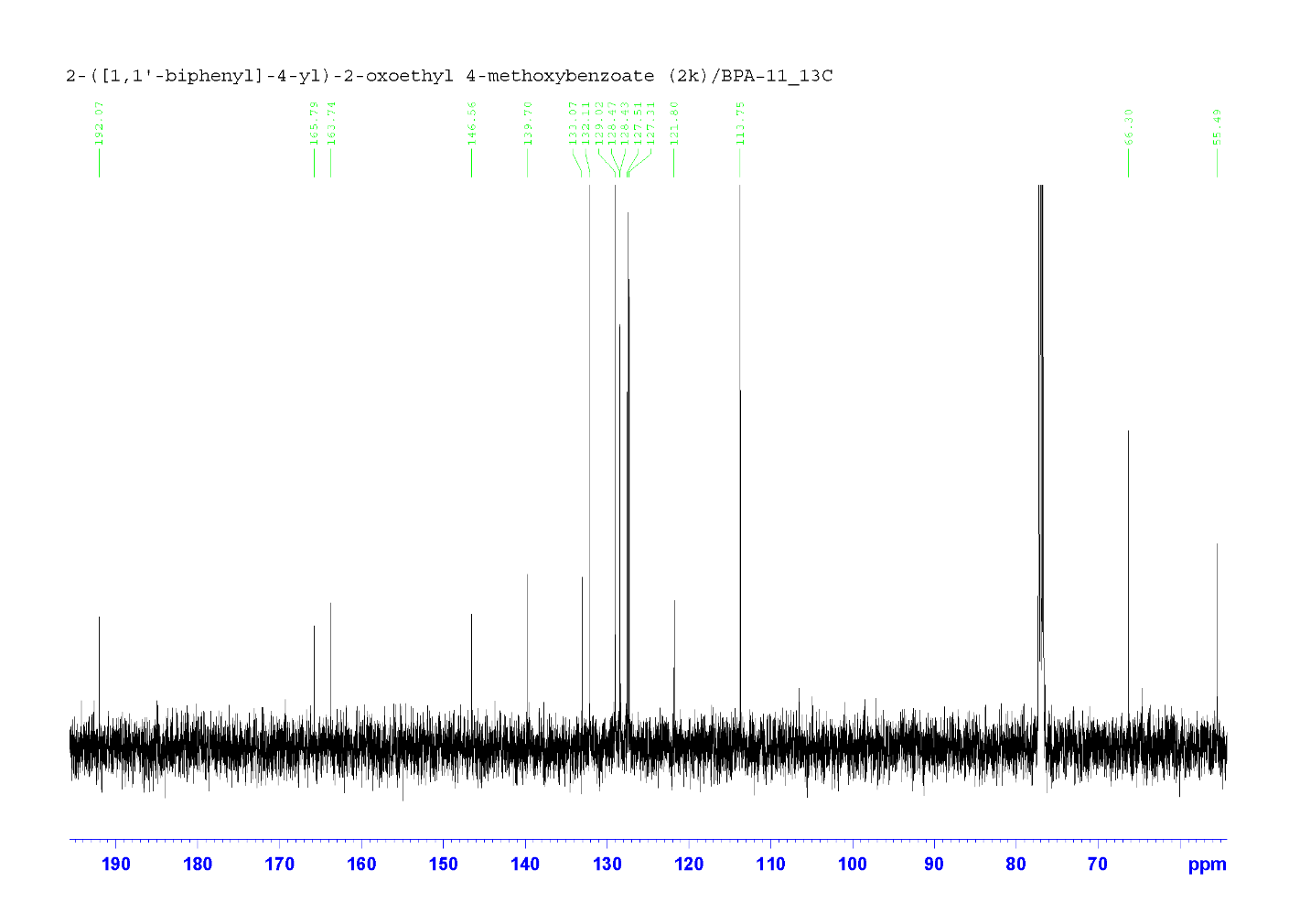
**

*2-([1,1'-biphenyl]-4-yl)-2-oxoethyl 4-methoxybenzoate* (**2k**)/BPA_11_FTIR

## 1.12 2-([1,1'-biphenyl]-4-yl)-2-oxoethyl 2-nitrobenzoate (**2l**)

**
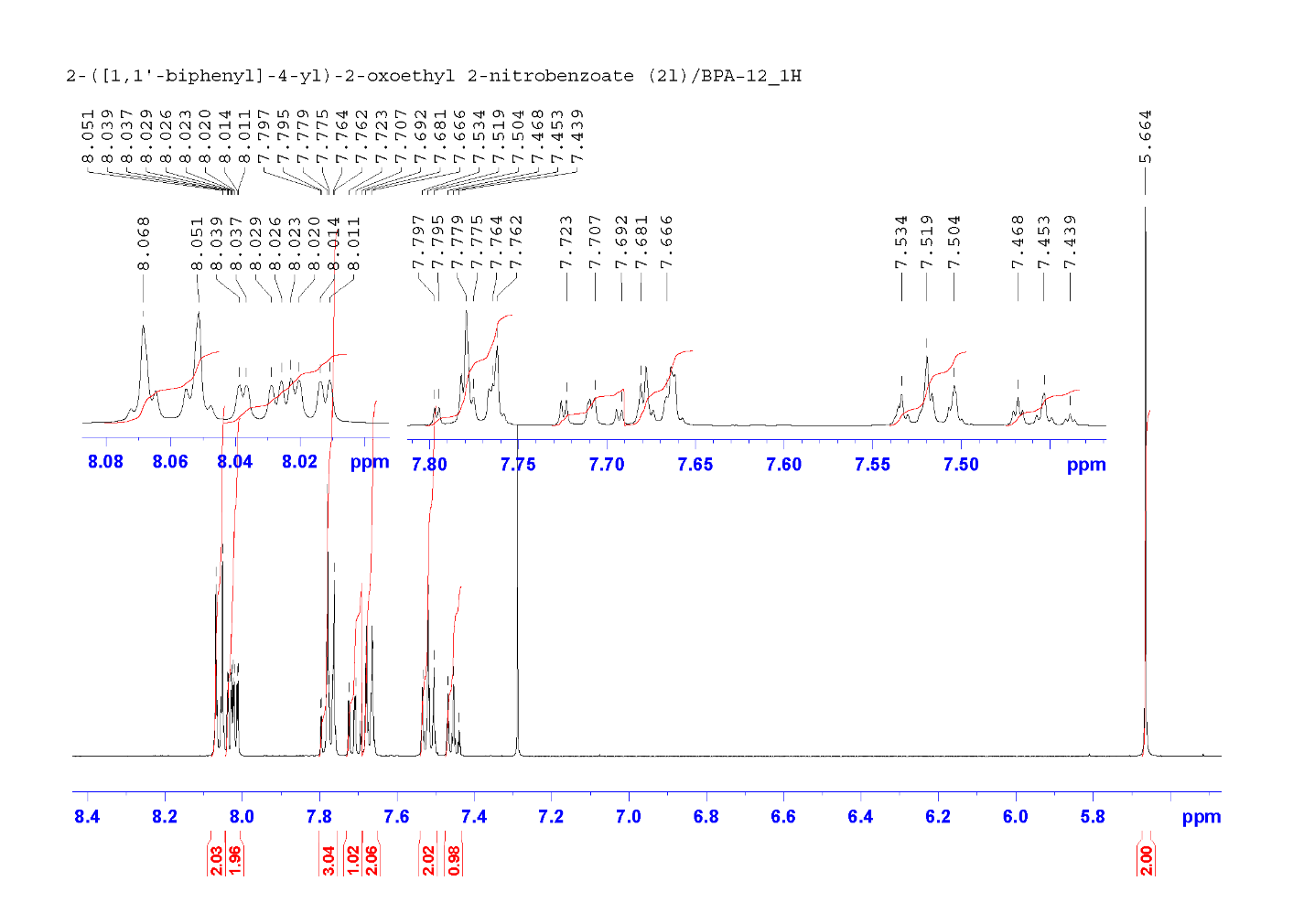
**

**
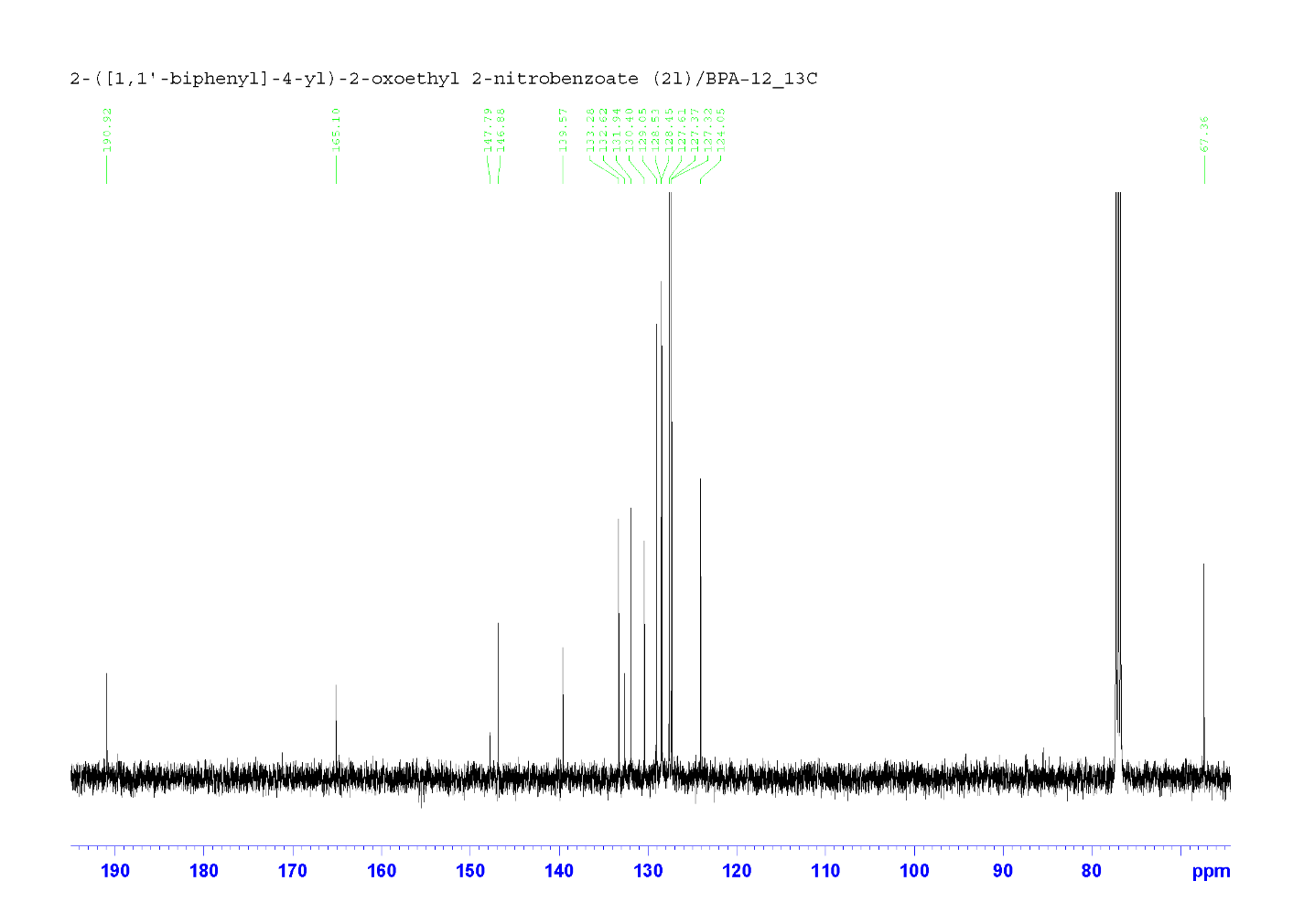
**

*2-([1,1'-biphenyl]-4-yl)-2-oxoethyl 2-nitrobenzoate* (**2l**)/BPA_12_FTIR

## 1.13 2-([1,1'-biphenyl]-4-yl)-2-oxoethyl 3-nitrobenzoate (**2m**)

**
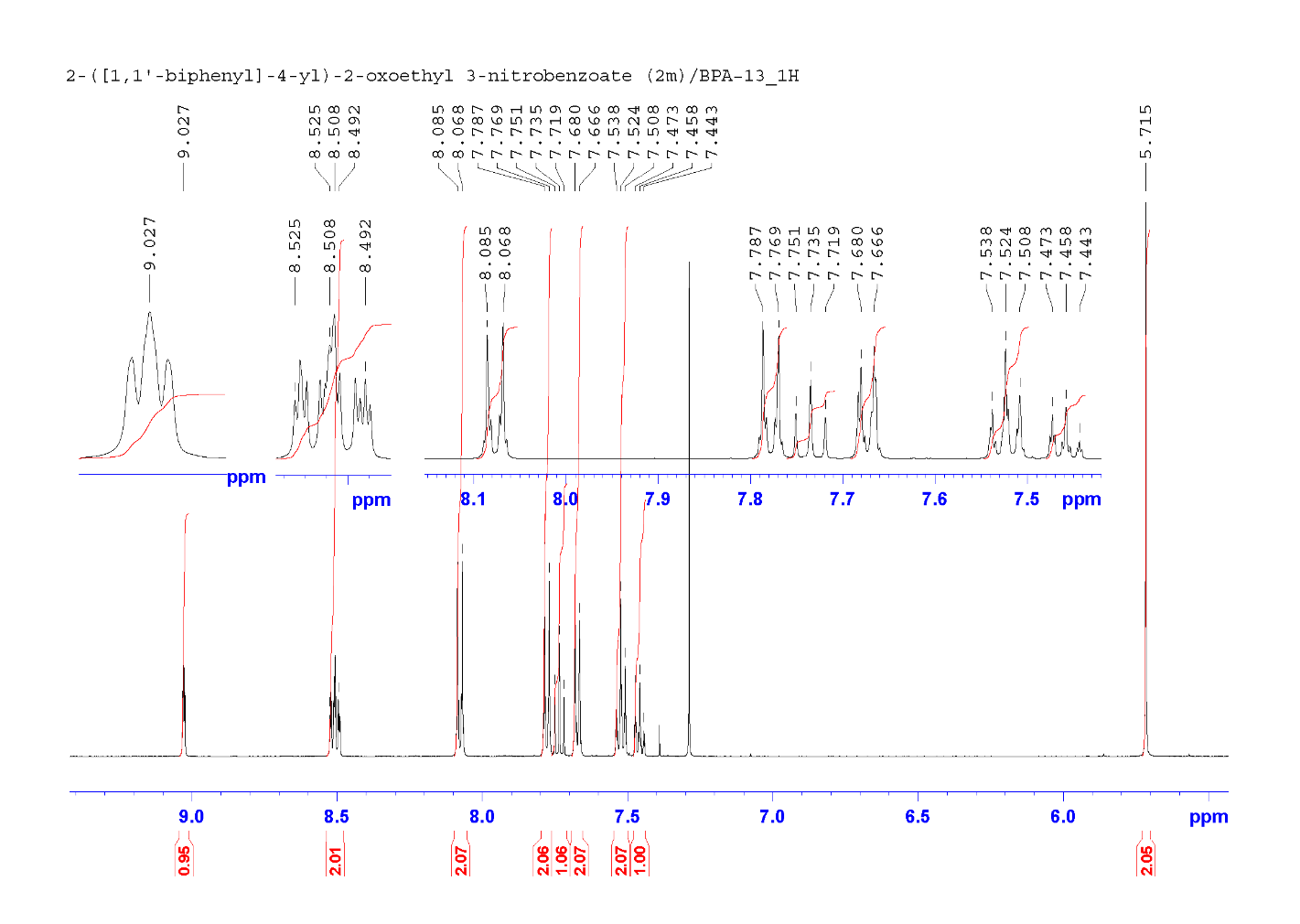
**

**
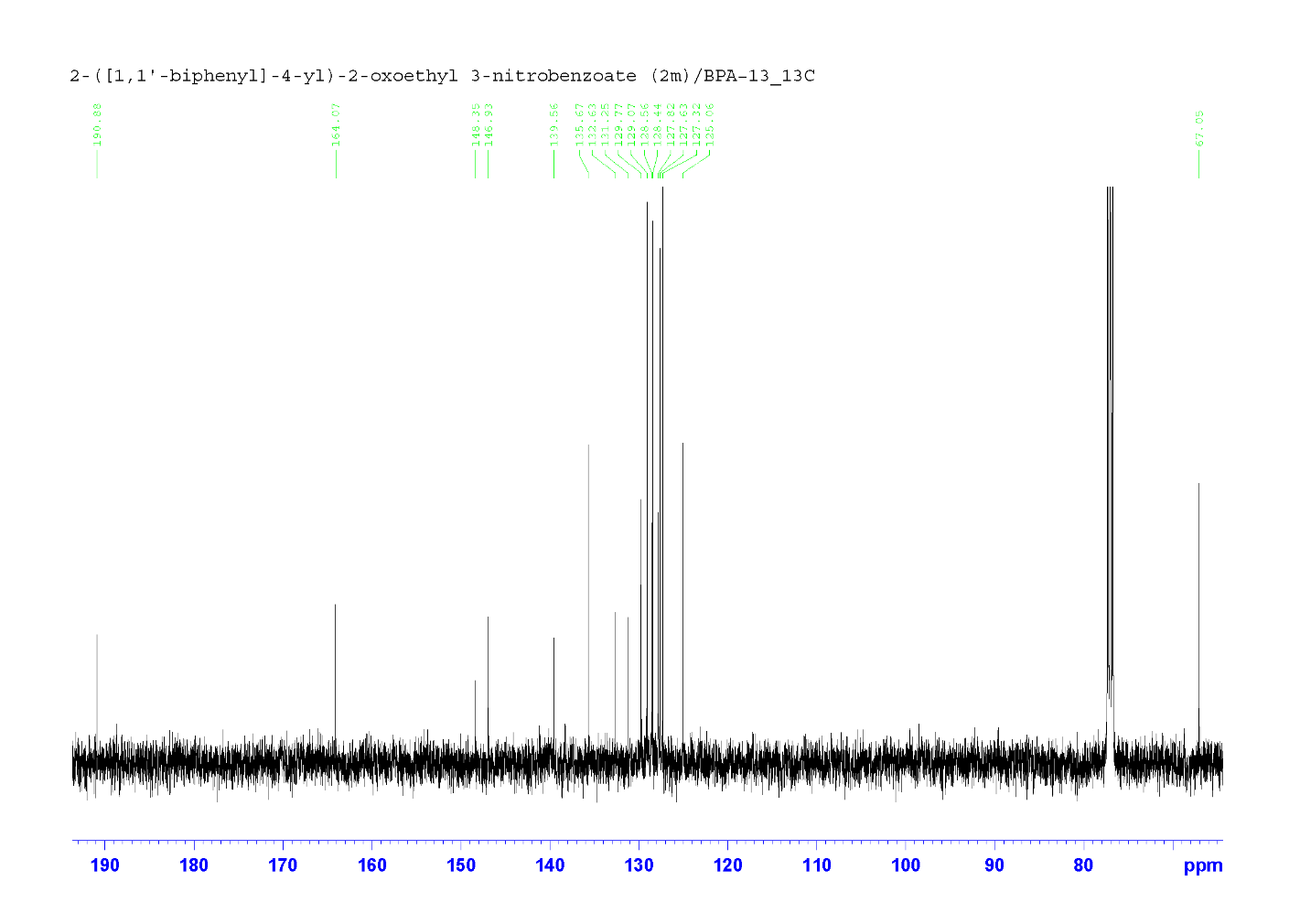
**

*2-([1,1'-biphenyl]-4-yl)-2-oxoethyl 3-nitrobenzoate* (**2m**)/BPA_13_FTIR

## 1.14 2-([1,1'-biphenyl]-4-yl)-2-oxoethyl 4-nitrobenzoate (**2n**)

**
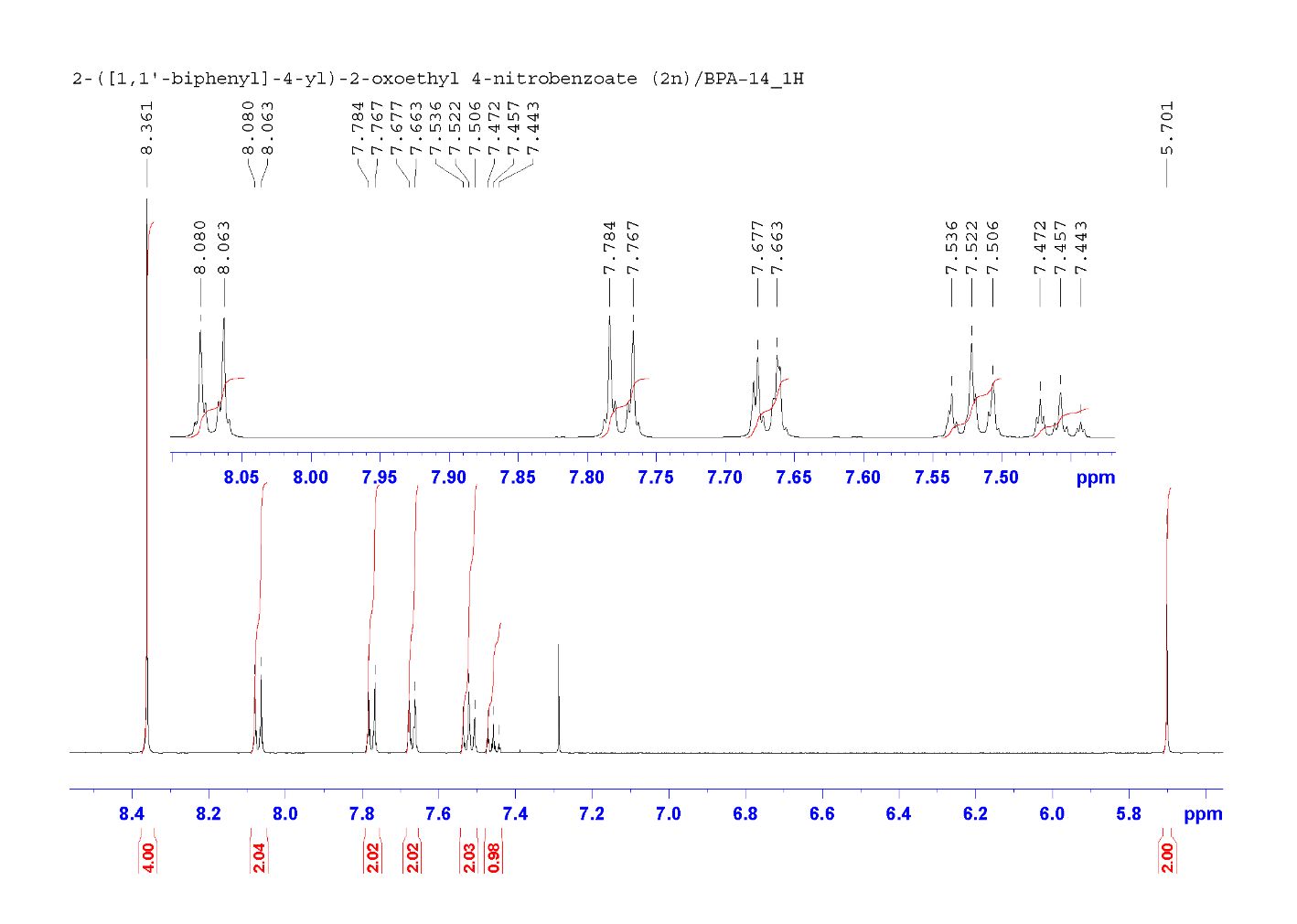
**

**
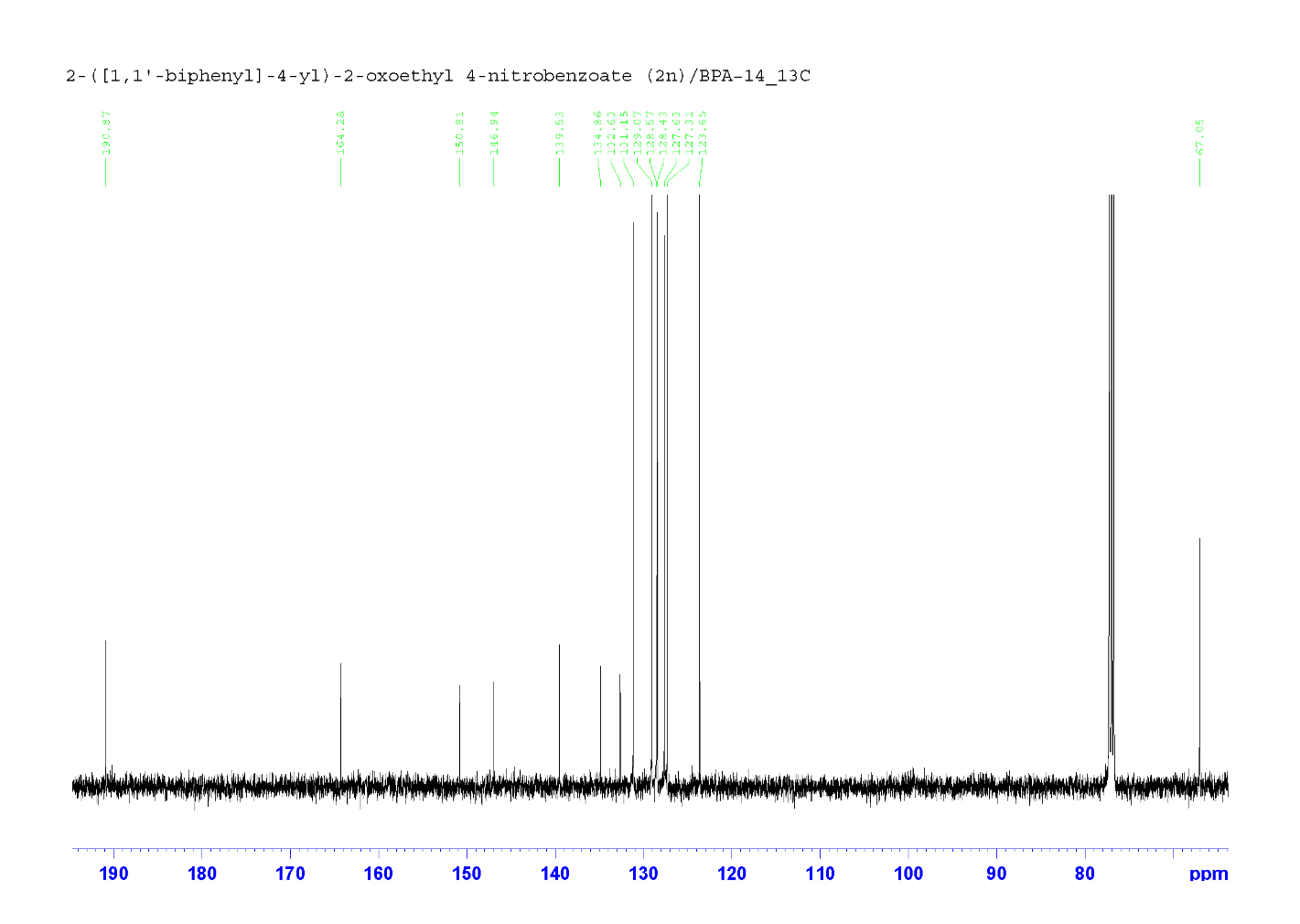
**

*2-([1,1'-biphenyl]-4-yl)-2-oxoethyl 4-nitrobenzoate* (**2n**)/BPA_14_FTIR

## 1.15 2-([1,1'-biphenyl]-4-yl)-2-oxoethyl 2-aminobenzoate (**2o**)

**
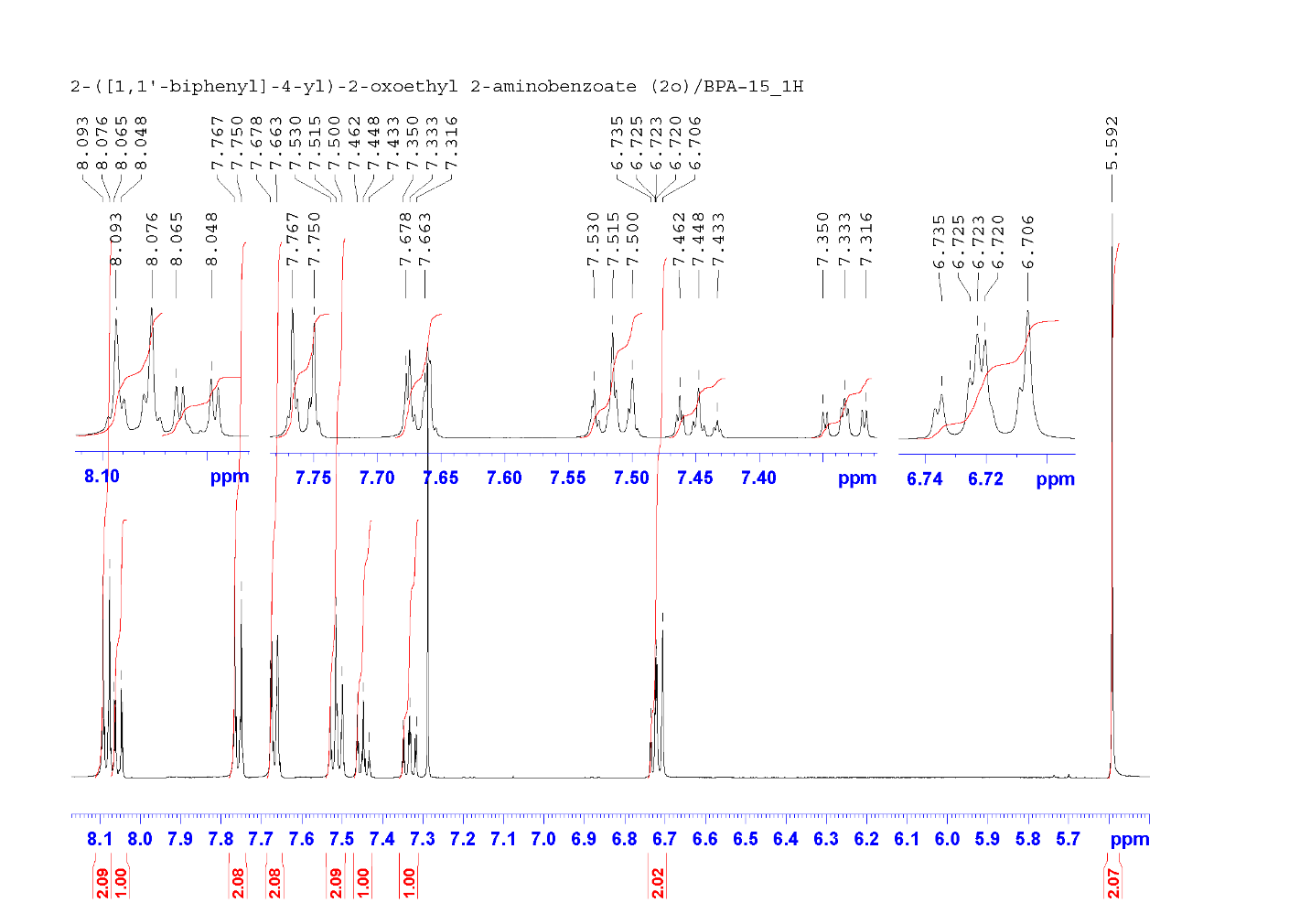
**

**
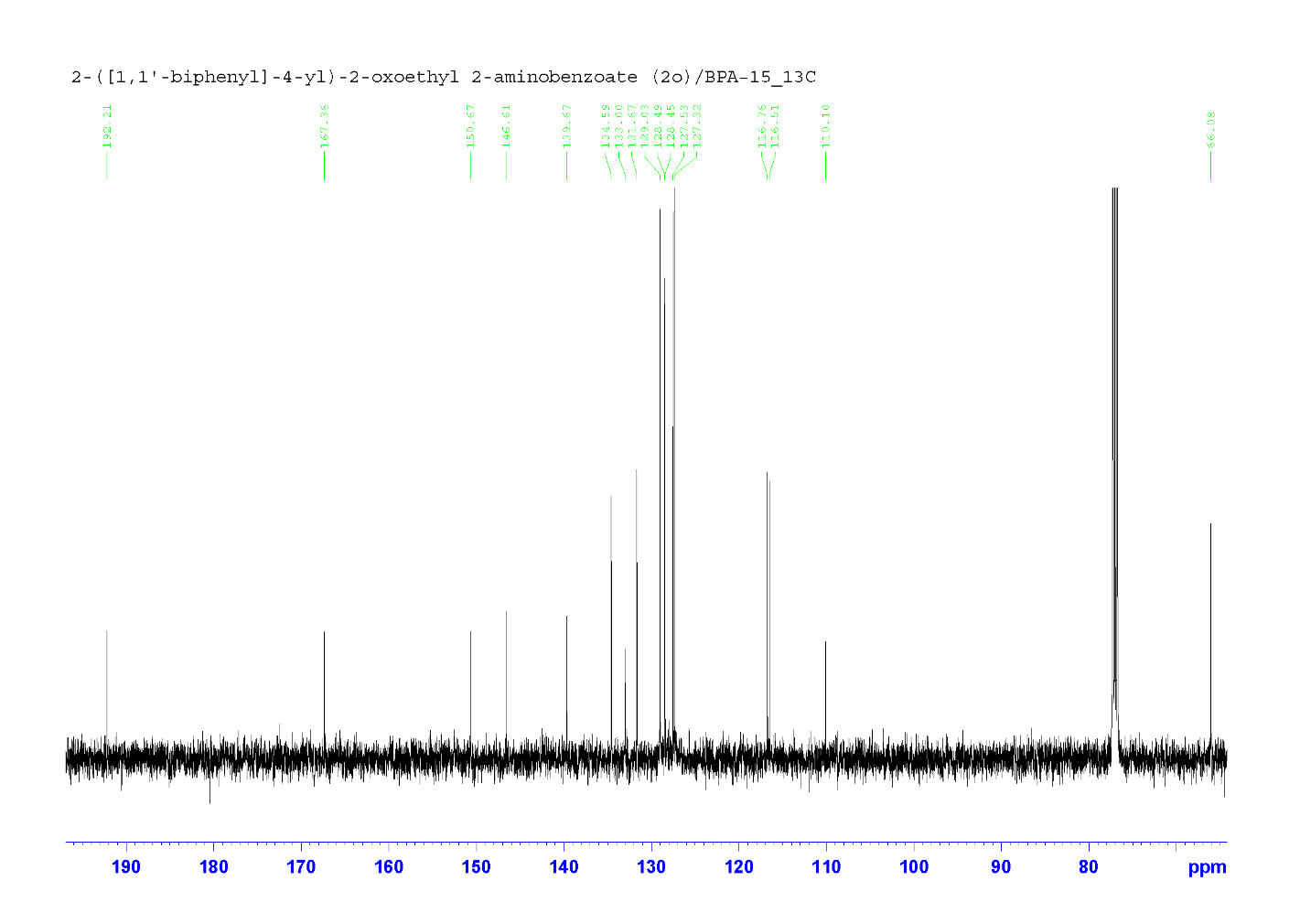
**

*2-([1,1'-biphenyl]-4-yl)-2-oxoethyl 2-aminobenzoate* (**2o**)/BPA_15_FTIR

## 1.16 2-([1,1'-biphenyl]-4-yl)-2-oxoethyl 3-aminobenzoate (**2p**)

**
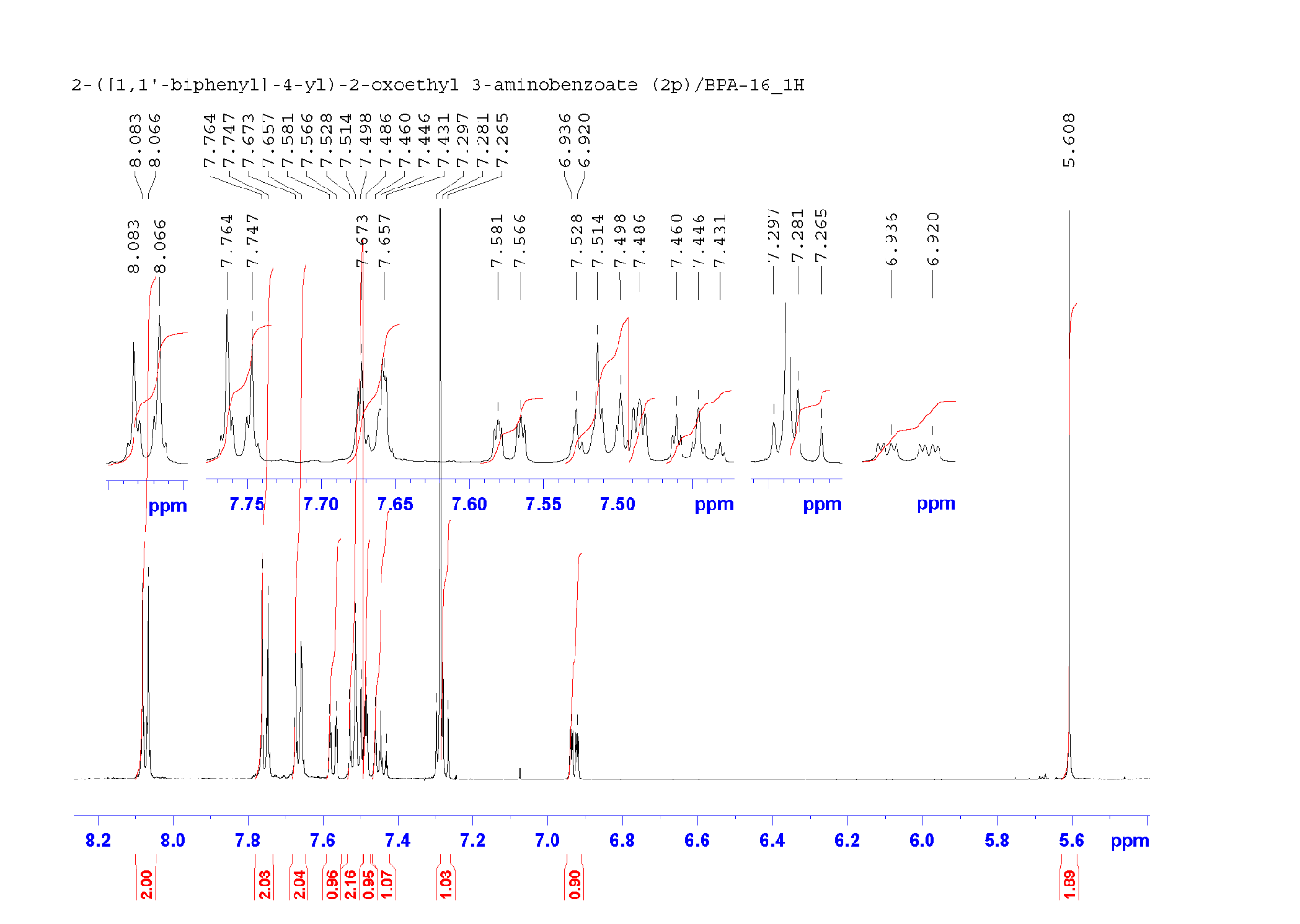
**

**
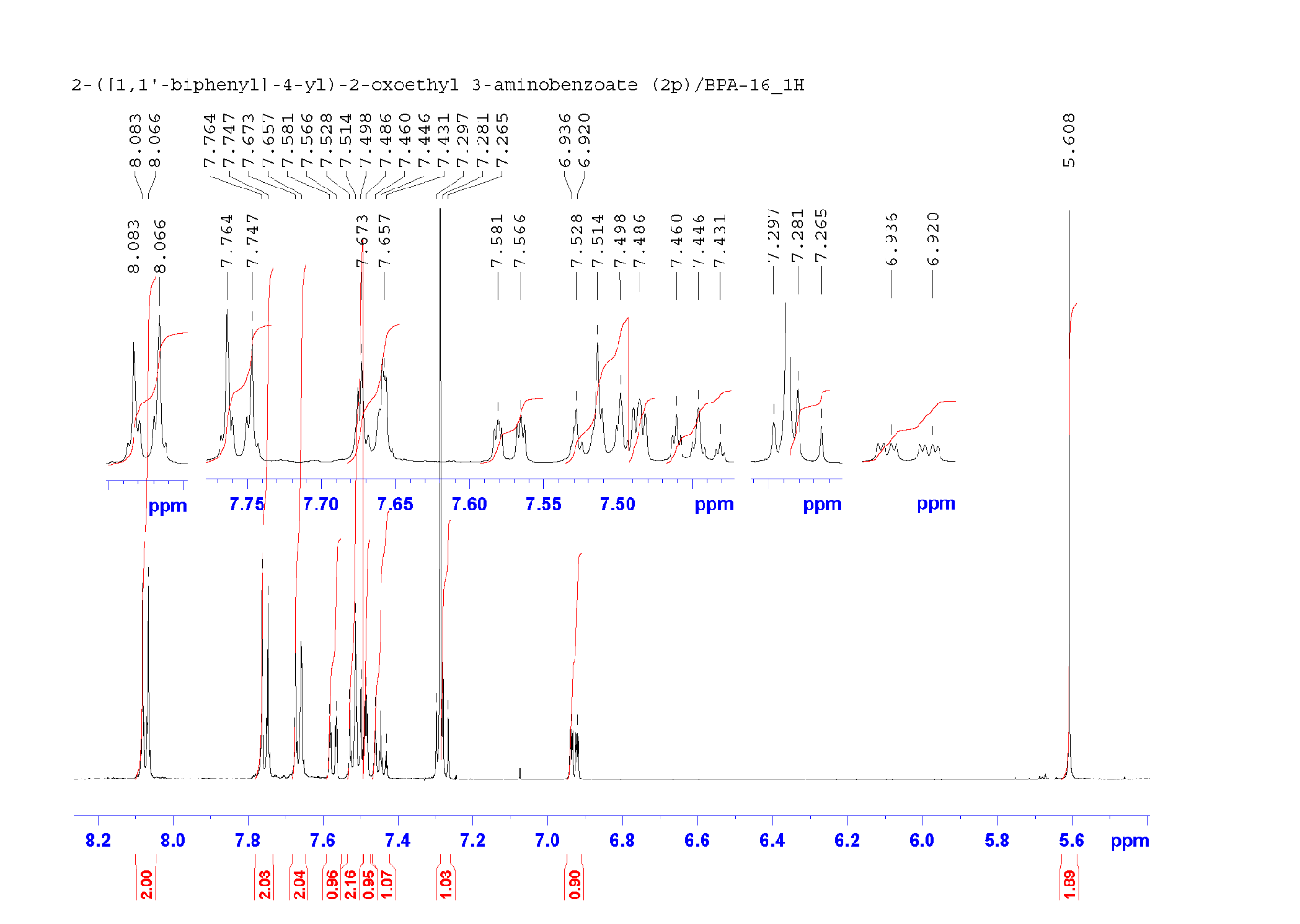
**

*2-([1,1'-biphenyl]-4-yl)-2-oxoethyl 3-aminobenzoate* (**2p**)/BPA_16_FTIR

## 1.17 2-([1,1'-biphenyl]-4-yl)-2-oxoethyl 4-aminobenzoate (**2q**)

**
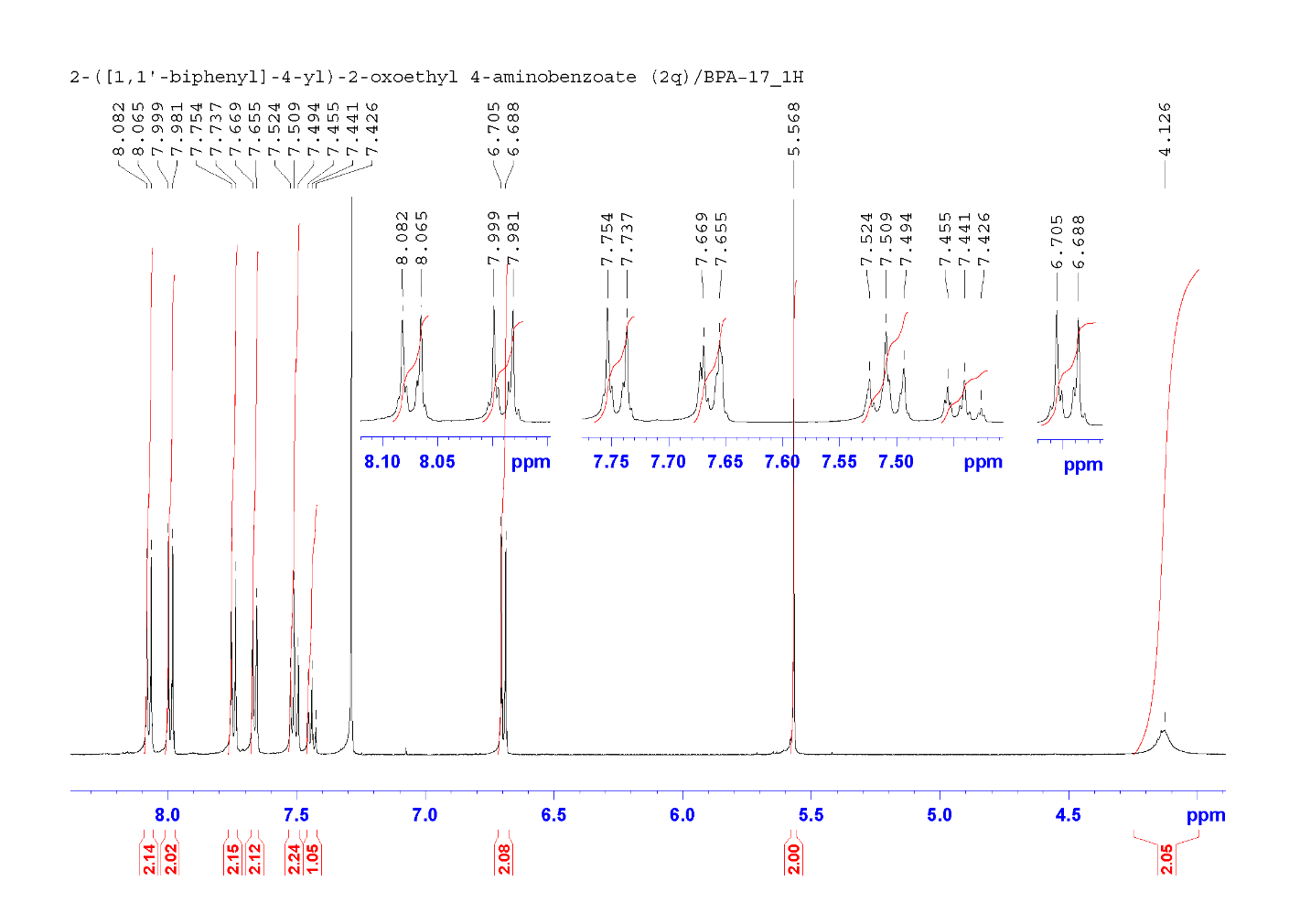
**

**
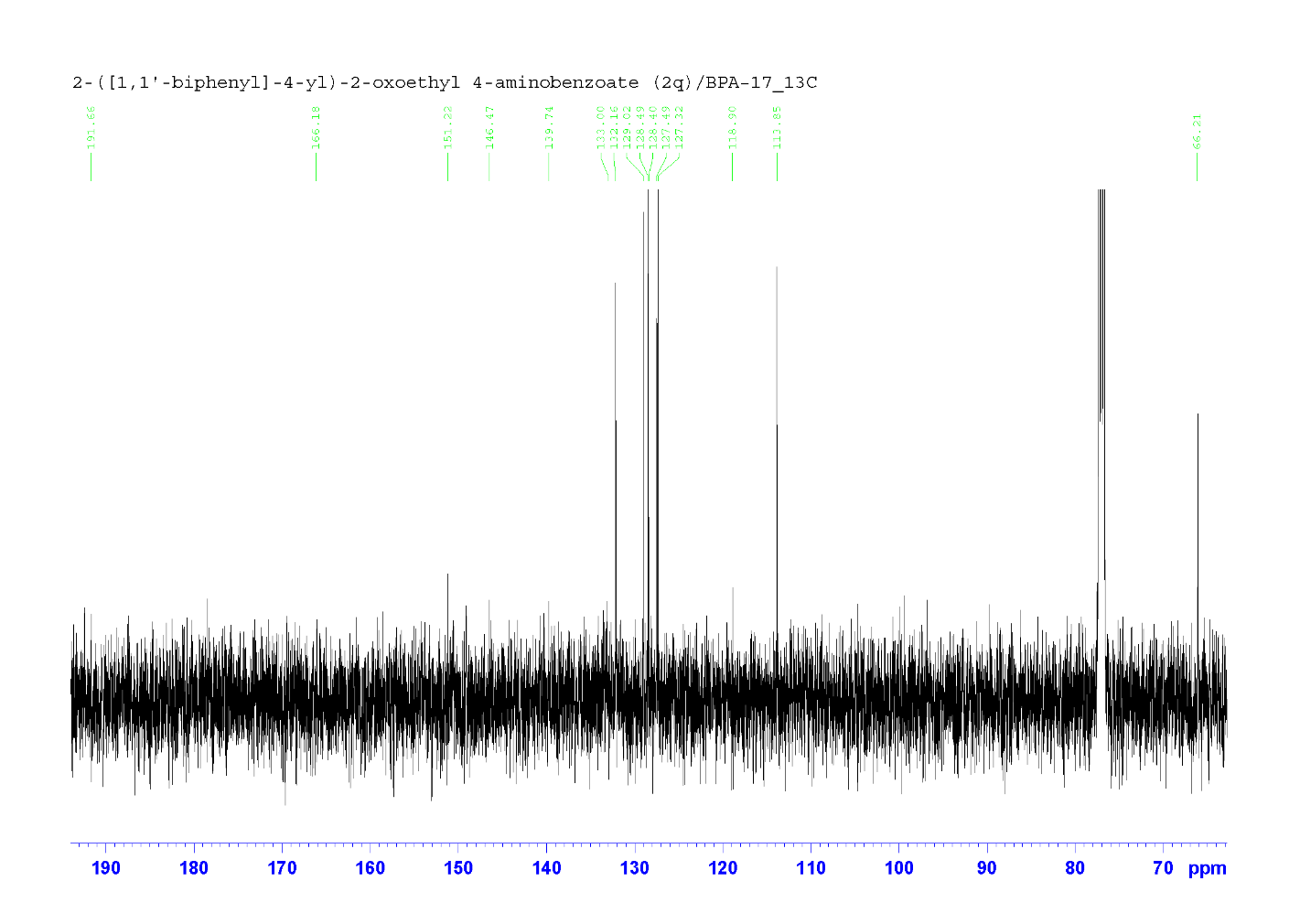
**

*2-([1,1'-biphenyl]-4-yl)-2-oxoethyl 4-aminobenzoate* (**2q**)/BPA_17_FTIR

## 1.18 2-([1,1'-biphenyl]-4-yl)-2-oxoethyl picolinate (**2r**)

**
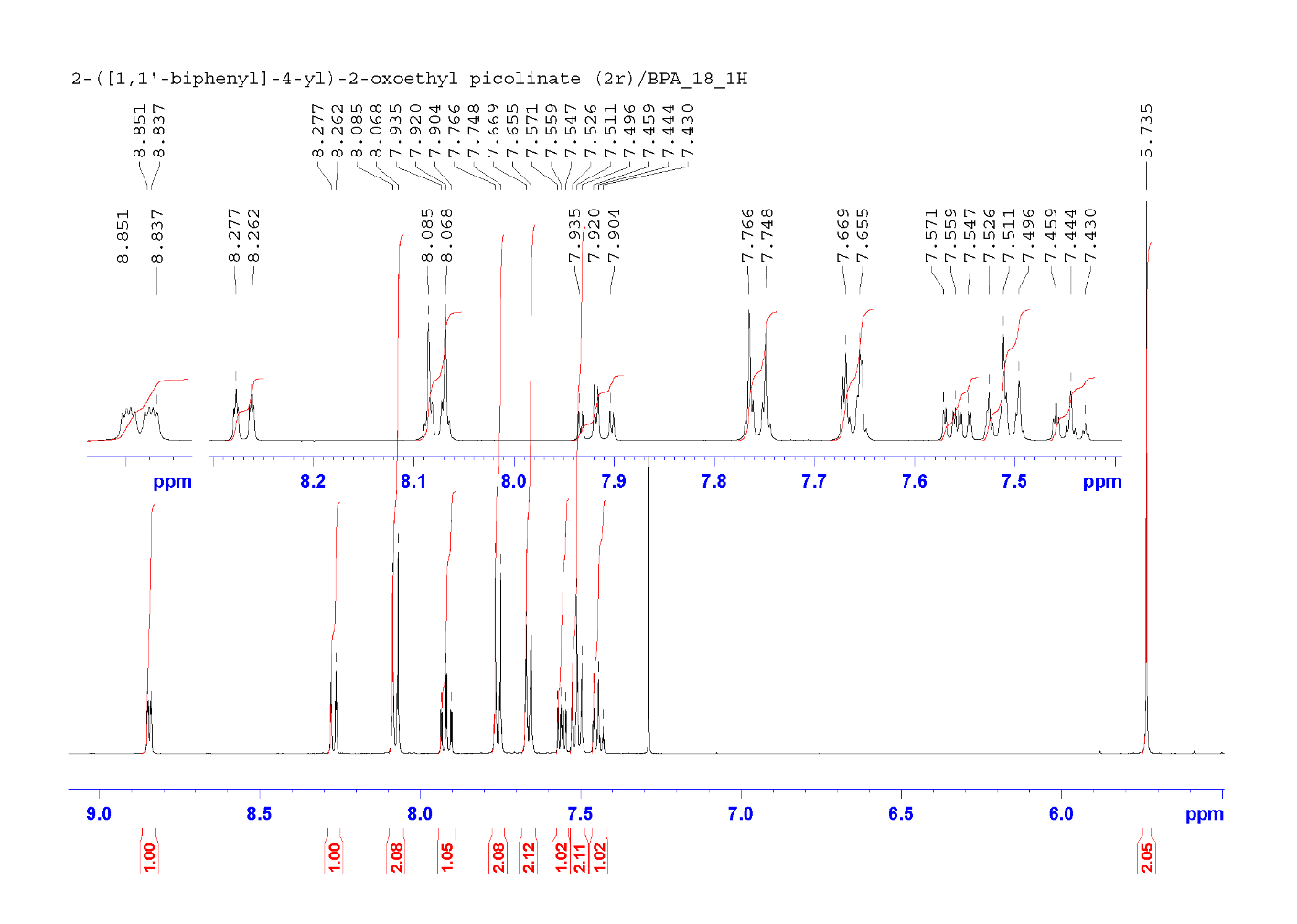
**

**
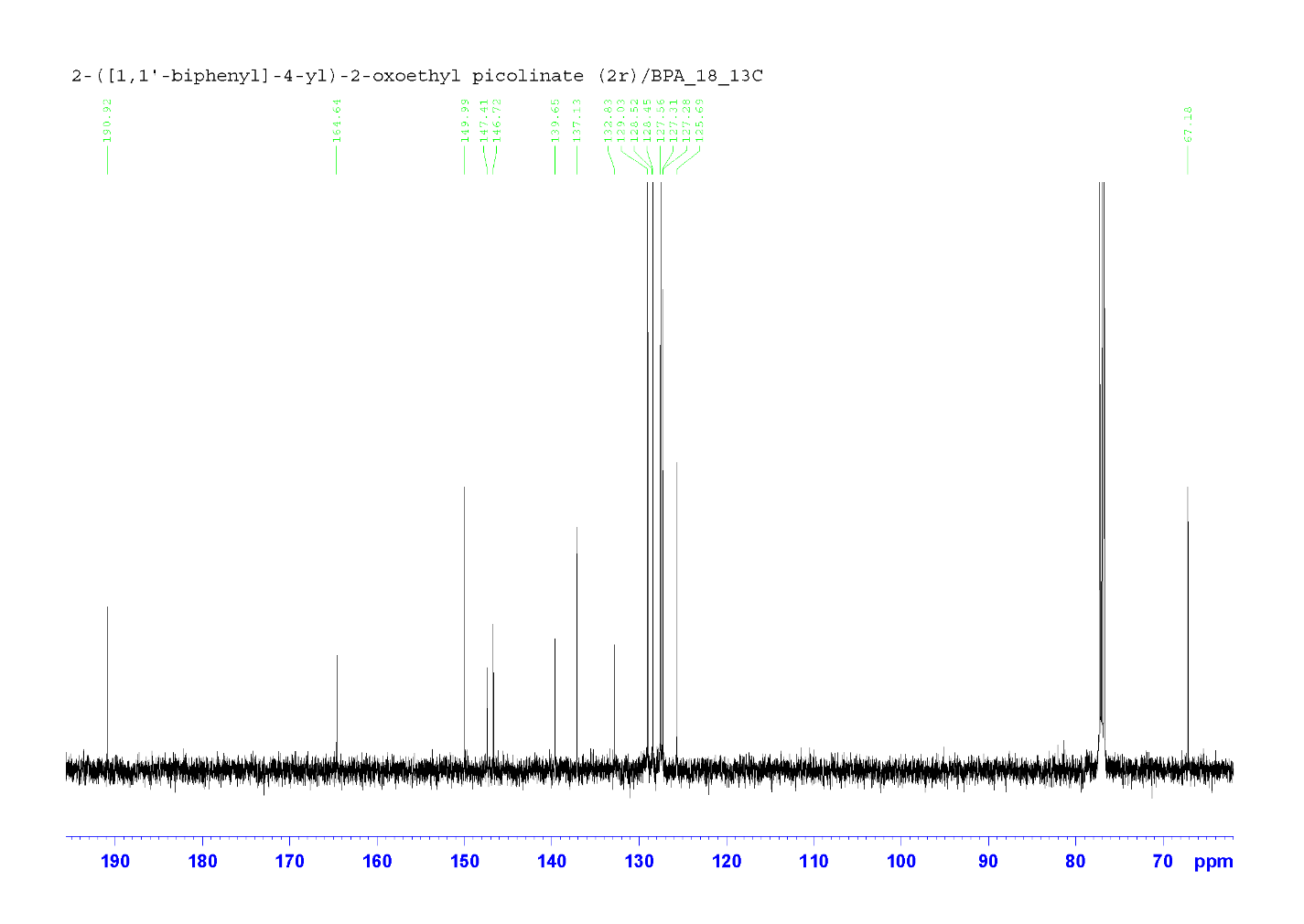
**

*2-([1,1'-biphenyl]-4-yl)-2-oxoethyl picolinate* (**2r**)/BPA_18_FTIR

## 1.19 2-([1,1'-biphenyl]-4-yl)-2-oxoethyl nicotinate (**2s**)

**
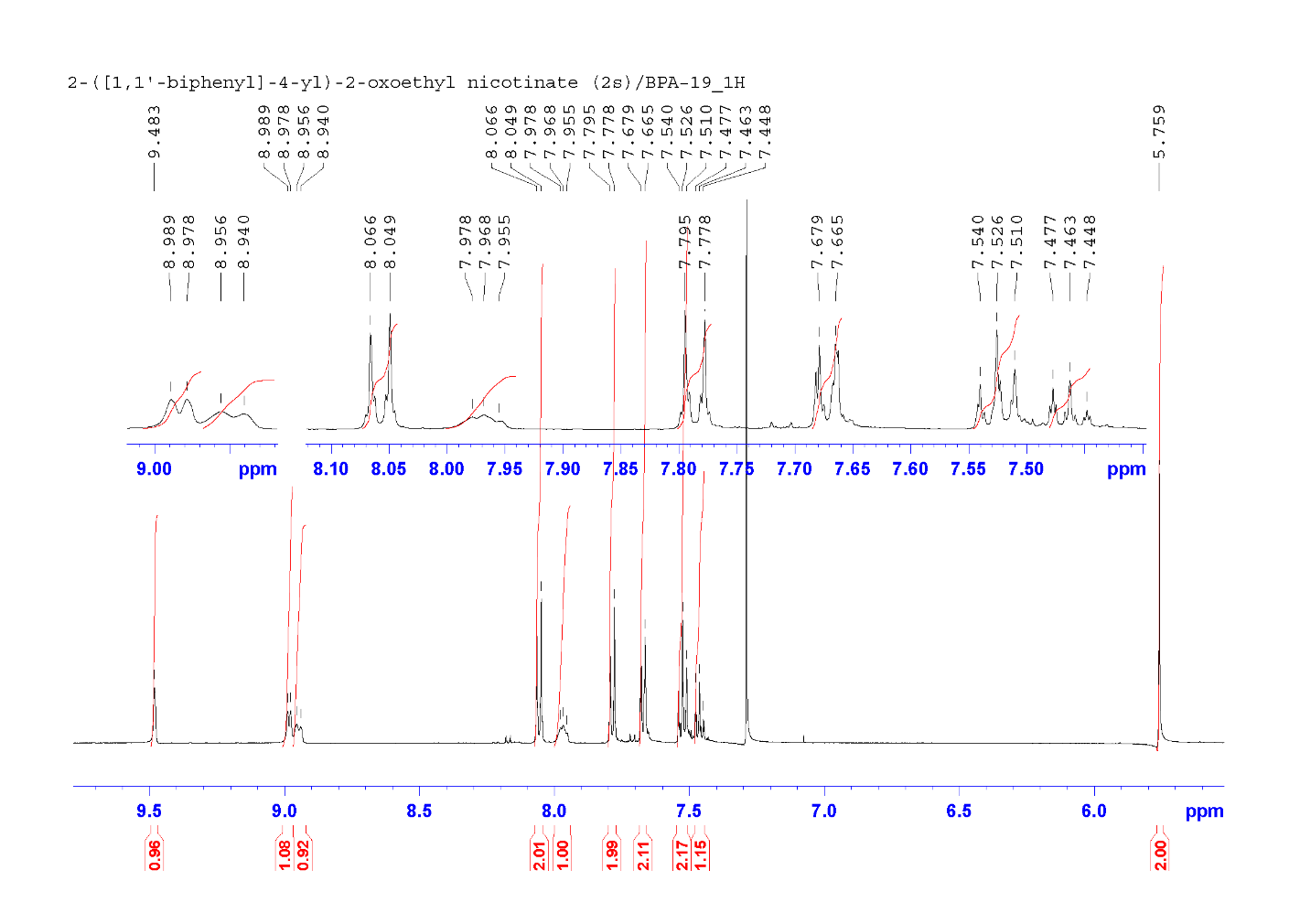
**

**

**

*2-([1,1'-biphenyl]-4-yl)-2-oxoethyl nicotinate* (**2s**)/BPA_19_FTIR

# 2.0 Single crystal X-ray crystallography data

## 2.1 Refined site occupancy

Orientational disorder of biphenyl group is observed in **2c**, **2n**, **2p**, **2q**, and **2r** with refined site-occupancy ratio near to 50: 50 as summarized in Table 2.1.

**Table 2.1.** Refined site-occupancy ratio of disordered biphenyl moiety.

| Compound | Refined site-occupancy ratio |
| --- | --- |
| **2c** | 0.500 : 0.500 |
| **2m** | 0.4998 (13): 0.5002 (13) |
| **2p** | 0.60 (2): 0.40 (2) |
| **2q** | 0.495 (8): 0.505 (8) |
| **2r** | 0.5041 (18): 0.4959 (18) |

## 2.2 Crystal data and parameters

Table 2.2: Crystal data and parameters for structure refinement for **2**(**b**-**e**, **g**, **i**-**l**)

| Compound | **2b** | **2c** | **2d** | **2e** | **2g** | **2i** | **2j** | **2k** | **2l** |
| --- | --- | --- | --- | --- | --- | --- | --- | --- | --- |
| CCDC deposition number | 1476974 | 1476976 | 1476979 | 1476977 | 1476975 | 1476980 | 1476978 | 1476981 | 1476982 |
| Molecular formula | C21H15ClO3 | C_21_H_15_ClO_3_ | C_21_H_15_ClO_3_ | C_21_H_14_Cl_2_O_3_ | C_22_H_18_O_3_ | C_22_H_18_O_4_ | C_22_H_18_O_4_ | C_22_H_18_O_4_ | C_21_H_15_NO_5_ |
| Molecular weight | 350.78 | 350.78 | 350.78 | 385.22 | 330.36 | 346.36 | 346.36 | 346.36 | 361.34 |
| Crystal system | Orthorhombic | Monoclinic | Orthorhombic | Monoclinic | Monoclinic | Orthorhombic | Monoclinic | Monoclinic | Orthorhombic |
| Space group | *Pbca* | *P*2_1_/*c* | *Pbca* | *P*2_1_/*c* | *P*2_1_ | *Pbca* | *P*2_1_/*c* | *P*2_1_ | *Pna*2_1_ |
| *a* (Å) | 11.3973 (19) | 5.0256 (6) | 9.7147 (6) | 12.5429 (13) | 5.2879 (13) | 17.341 (2) | 10.7254 (7) | 8.6594 (14) | 9.8435 (14) |
| *b* (Å) | 8.0357 (14) | 9.2766 (11) | 9.4386 (5) | 8.6583 (9) | 8.975 (2) | 8.9197 (11) | 9.0722 (6) | 5.2690 (8) | 32.312 (5) |
| *c* (Å) | 36.509 (6) | 36.530 (4) | 36.285 (2) | 17.4461 (19) | 17.996 (4) | 22.412 (3) | 17.3681 (11) | 19.270 (3) | 5.3678 (8) |
| α (°) | 90 | 90 | 90 | 90 | 90 | 90 | 90 | 90 | 90 |
| β (°) | 90 | 91.228 (2) | 90 | 108.990 (2) | 93.829 (4) | 90 | 93.3386 (12) | 91.783 (3) | 90 |
| γ (°) | 90 | 90 | 90 | 90 | 90 | 90 | 90 | 90 | 90 |
| *V* (Å^3^) | 3343.7 (10) | 1702.7 (3) | 3327.1 (3) | 1791.5 (3) | 852.2 (4) | 3466.5 (8) | 1687.10 (19) | 878.8 (2) | 1707.3 (4) |
| *Z* | 8 | 4 | 8 | 4 | 2 | 8 | 4 | 2 | 4 |
| *D_calc_* (g cm^−3^) | 1.394 | 1.368 | 1.401 | 1.428 | 1.287 | 1.327 | 1.364 | 1.309 | 1.406 |
| Crystal dimensions (mm) | 0.25 × 0.23 × 0.11 | 0.26 × 0.13 × 0.08 | 0.32 × 0.22 × 0.10 | 0.42 × 0.26 × 0.15 | 0.61 × 0.30 × 0.14 | 0.31 × 0.13 × 0.03 | 0.34 × 0.24 × 0.13 | 0.39 × 0.37 × 0.14 | 0.50 × 0.22 × 0.06 |
| μ (mm^−1^) | 0.25 | 0.24 | 0.25 | 0.38 | 0.09 | 0.09 | 0.09 | 0.09 | 0.1 |
| T_min_/T_max_ | 0.803/0.917 | 0.854/0.939 | 0.808/0.914 | 0.772/0.838 | 0.823/0.956 | 0.798/0.965 | 0.886/0.964 | 0.819/0.955 | 0.868/0.968 |
| Reflections measured | 15748 | 19400 | 28983 | 17975 | 6356 | 57291 | 18878 | 6802 | 9935 |
| Ranges/indices (*h*, *k*, *l*) | −14→14, −7→10, −31→46 | −6→6, −12→12, −48→47 | −13→13, −12→11, −49→49 | −17→17, −10→12, −24→24 | −6→6, −11→11, −23→18 | −22→22, −11→11, −29→29 | −15→14, −12→12, −24→24 | −10→10, −6→5, −23→20 | −13→11, −38→44, −7→7 |
| θ limit (°) | 2.9–22.6 | 2.3–21.7 | 2.3–29.1 | 2.5–22.8 | 2.5–22.8 | 2.2–21.6 | 2.5–33.0 | 2.4–25.0 | 2.2–25.8 |
| Unique reflections | 3625 | 4107 | 4502 | 5255 | 3865 | 4191 | 4877 | 3113 | 4536 |
| Observed reflections [*I*> 2σ(*I*)] | 1673 | 1997 | 3620 | 2725 | 2685 | 2421 | 3969 | 2385 | 3586 |
| Parameters | 226 | 298 | 226 | 235 | 227 | 236 | 236 | 236 | 244 |
| Restraints | 0 | 0 | 0 | 0 | 1 | 0 | 0 | 1 | 1 |
| Goodness of fit on *F*^2^ | 1.02 | 0.94 | 1.03 | 1.03 | 1.03 | 1.02 | 1.03 | 1.05 | 1.03 |
| *R_1_*, *wR_2_* [*I* ≥ 2σ(*I*)] | 0.055, 0.208 | 0.048, 0.156 | 0.039, 0.102 | 0.051, 0.175 | 0.046, 0.139 | 0.052, 0.140 | 0.042, 0.134 | 0.044, 0.114 | 0.036, 0.133 |

Table 2.3: Crystal data and parameters for structure refinement for **2**(**n-s**)

| Compound | **2n** | **2m** | **2o** | **2p** | **2q** | **2r** | **2s** |
| --- | --- | --- | --- | --- | --- | --- | --- |
| CCDC deposition number | 1477101 | 1477102 | 1477103 | 1477107 | 1477104 | 1477105 | 1477106 |
| Molecular formula | C_21_H_15_NO_5_ | C_21_H_15_NO_5_ | C_21_H_17_NO_3_ | C_21_H_17_NO_3_ | C_21_H_17_NO_3_ | C_20_H_15_NO_3_ | C_20_H_15_NO_3_ |
| Molecular weight | 361.34 | 361.34 | 331.36 | 331.36 | 331.36 | 317.33 | 317.33 |
| Crystal system | Triclinic | Monoclinic | Orthorhombic | Triclinic | Monoclinic | Triclinic | Triclinic |
| Space group | *P1 ̅* | *P*2_1_/*c* | *Pbca* | *P1 ̅* | *P*2_1_ | *P1 ̅* | *P1 ̅* |
| *a* (Å) | 4.9326 (3) | 8.6808 (12) | 10.6613 (13) | 5.2933 (6) | 8.3297 (8) | 9.0750 (9) | 9.1620 (8) |
| *b* (Å) | 18.3120 (12) | 5.5549 (8) | 9.8240 (12) | 11.6818 (14) | 5.0651 (5) | 9.5322 (9) | 10.2348 (9) |
| *c* (Å) | 19.1670 (13) | 35.401 (5) | 32.235 (4) | 13.4035 (17) | 19.4672 (19) | 17.8761 (17) | 17.1840 (15) |
| α (°) | 103.2153 (11) | 90 | 90 | 87.855 (3) | 90 | 95.820 (2) | 86.590 (2) |
| β (°) | 97.4078 (8) | 90.118 (2) | 90 | 83.982 (3) | 95.7119 (16) | 91.027 (4) | 77.632 (2) |
| γ (°) | 89.9827 (8) | 90 | 90 | 87.705 (3) | 90 | 103.853 (1) | 87.622 (2) |
| *V* (Å^3^) | 1670.58 (19) | 1707.1 (4) | 3376.1 (7) | 823.12 (17) | 817.26 (14) | 1492.2 (2) | 1570.5 (2) |
| *Z* | 4 | 4 | 8 | 2 | 2 | 4 | 4 |
| *D_calc_* (g cm^−3^) | 1.437 | 1.406 | 1.304 | 1.337 | 1.347 | 1.412 | 1.342 |
| Crystal dimensions (mm) | 0.59 × 0.15 × 0.07 | 0.64 × 0.33 × 0.23 | 0.51 × 0.23 × 0.06 | 0.62 × 0.15 × 0.09 | 0.40 × 0.21 × 0.12 | 0.39 × 0.29 × 0.04 | 0.30 × 0.24 × 0.05 |
| μ (mm^−1^) | 0.1 | 0.1 | 0.09 | 0.09 | 0.09 | 0.1 | 0.09 |
| T_min_/T_max_ | 0.891/0.968 | 0.841/0.935 | 0.807/0.966 | 0.853/0.968 | 0.906/0.962 | 0.865/0.975 | 0.913/0.974 |
| Reflections measured | 44916 | 40816 | 18258 | 16326 | 12380 | 29006 | 42437 |
| Ranges/indices (*h*, *k*, *l*) | −6→6, −23→23, −24→24 | −12→12, −8→8, −51→51 | −14→12, −13→13, −30→44 | −7→7, −15→15, −18→18 | −10→10, −6→6, −25→25 | −12→12, −13→13, −24→24 | −11→11, −13→13, −22→22 |
| θ limit (°) | 2.2–27.5 | 2.3–30.3 | 2.9–22.1 | 2.3–21.8 | 2.5–27.6 | 2.6–29.2 | 2.3–20.3 |
| Unique reflections | 7687 | 5508 | 4506 | 4391 | 3818 | 8005 | 7274 |
| Observed reflections [*I*> 2σ(*I*)] | 5773 | 3933 | 1975 | 2019 | 3369 | 5388 | 3495 |
| Parameters | 632 | 245 | 234 | 271 | 271 | 578 | 433 |
| Restraints | 16 | 0 | 0 | 0 | 1 | 0 | 0 |
| Goodness of fit on *F*^2^ | 0.98 | 1.05 | 0.96 | 1.05 | 1.05 | 1.06 | 1.01 |
| *R_1_*, *wR_2_* [*I* ≥ 2σ(*I*)] | 0.042, 0.143 | 0.053, 0.165 | 0.048, 0.160 | 0.055, 0.208 | 0.036, 0.087 | 0.057, 0.185 | 0.054, 0.172 |

## 2.3 Ortep diagram and atom numbering


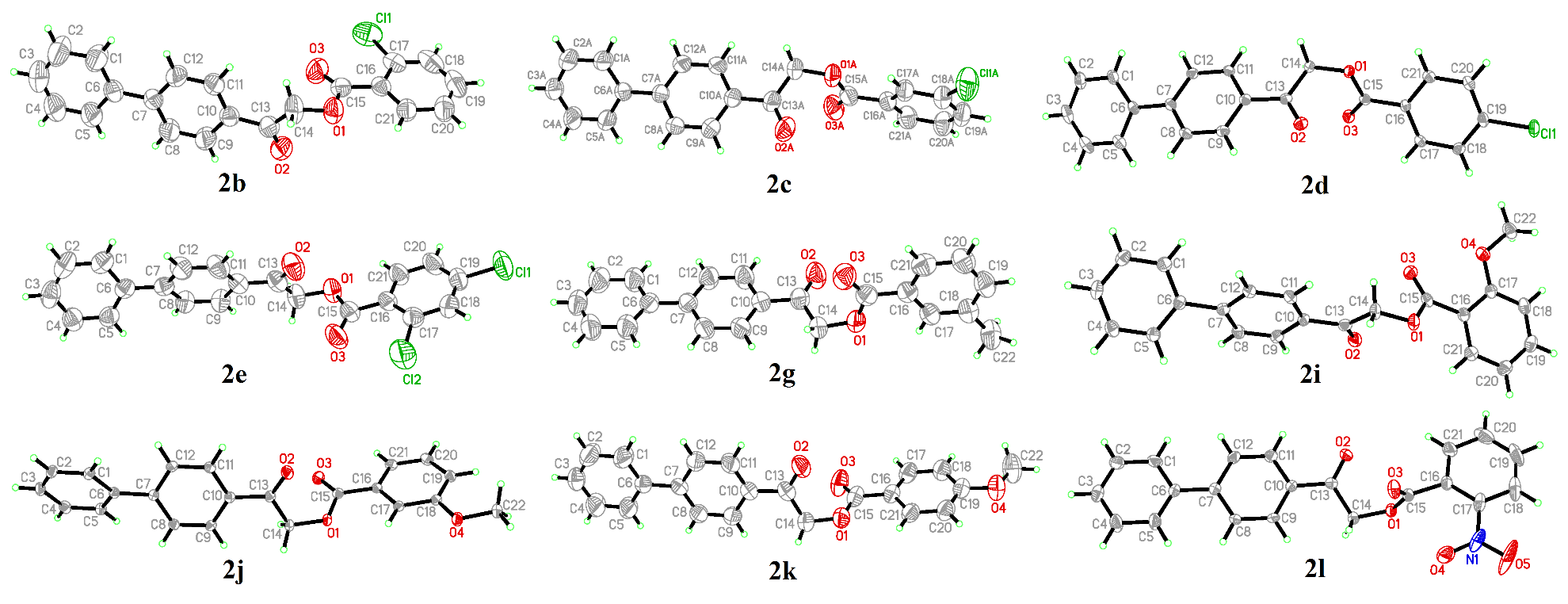
**Figure 2.1:** The molecular structures of **2**(**b**-**e**, **g**, **i**-**l**)with atom numbering schemes and 50% probability displacement ellipsoids. Atoms of the disordered moiety (**2c**) were omitted for clarity.


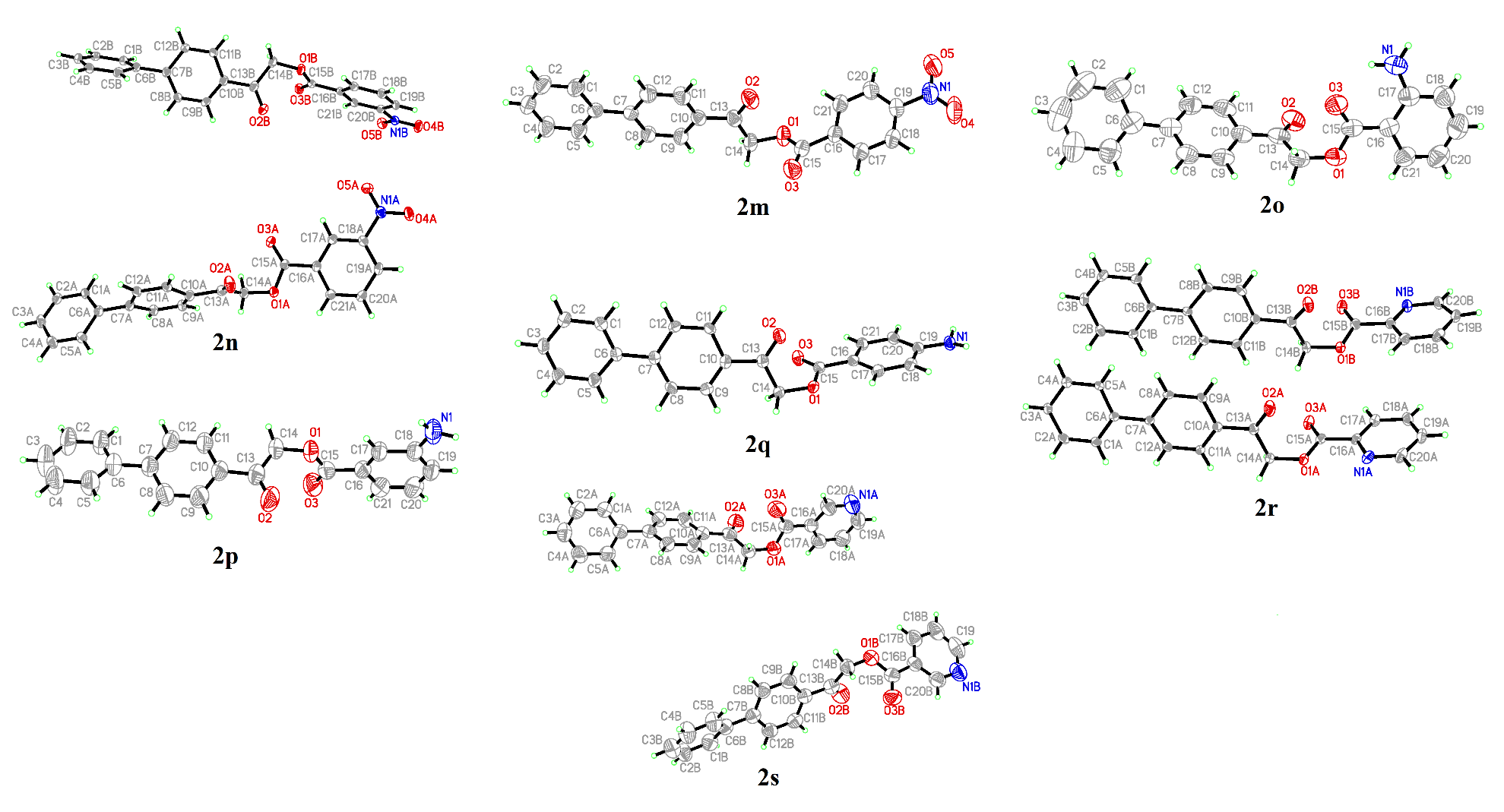


**Figure 2.2:** The molecular structures of **2**(**n-s**)with atom numbering schemes and 50% probability displacement ellipsoids. Atoms of the disordered moiety (**2m**, **2p**, **2q**, **2r**) were omitted for clarity.

## 2.4 Supramolecular feature

In the crystal packing of **2b**, molecules are linked into a chain along *b*-axis by C14—H14*B*···O2 hydrogen bonds (Figure 2.3a). Those chains are linked by slipped parallel π···π interactions involving inversion related 2-chlorobenzene rings [*Cg*1···*Cg*1^i^ = 3.564 (2) Å; *Cg*1 is the centroid of the chlorobenzene rings (C16-C21); interplanar distances = 3.438 Å; slippage = 0.938; symmetry code: (i) 1-*x*, -*y*, 1-*z*.] (Figure 2.3b). While in the crystal of 2c, molecules are linked into a centrosymmetric dimer *via* a weak C4*A*—H4*AA*···O3*A* hydrogen bond and forming a $R_{2}^{2}$(26) ring motive. Those dimers are linked into a 2D sheet along [-102] direction thru a week C19*A*—H19*A*···O2*A* hydrogen bond (Figure 2.4).


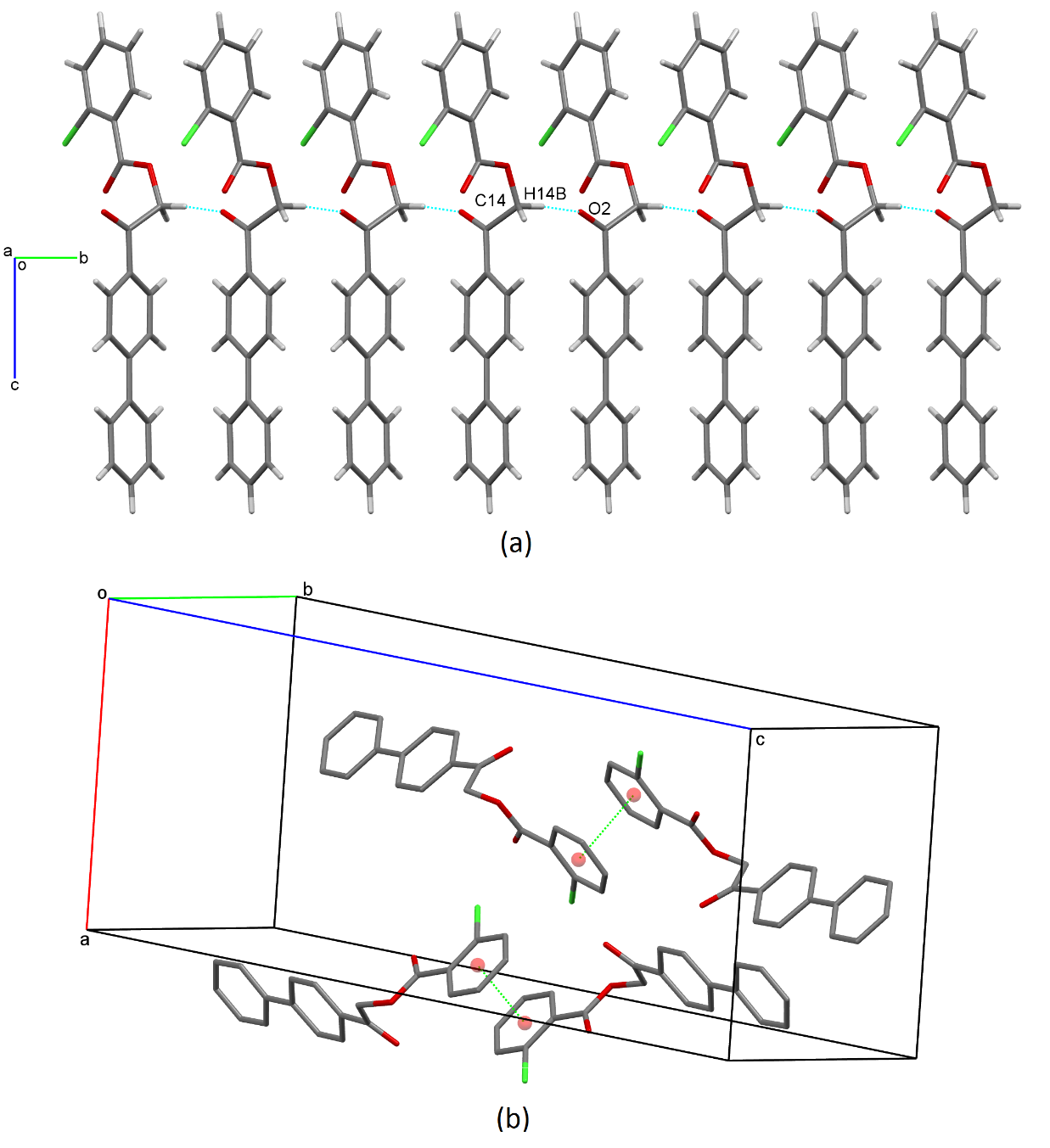


**Figure 2.3: (a)** Partial packing diagram of **2b** shows hydrogen-bonded column along *b*-axis. **(b)** Partial packing diagram of **2b** showing the inversion related π···π interactions.


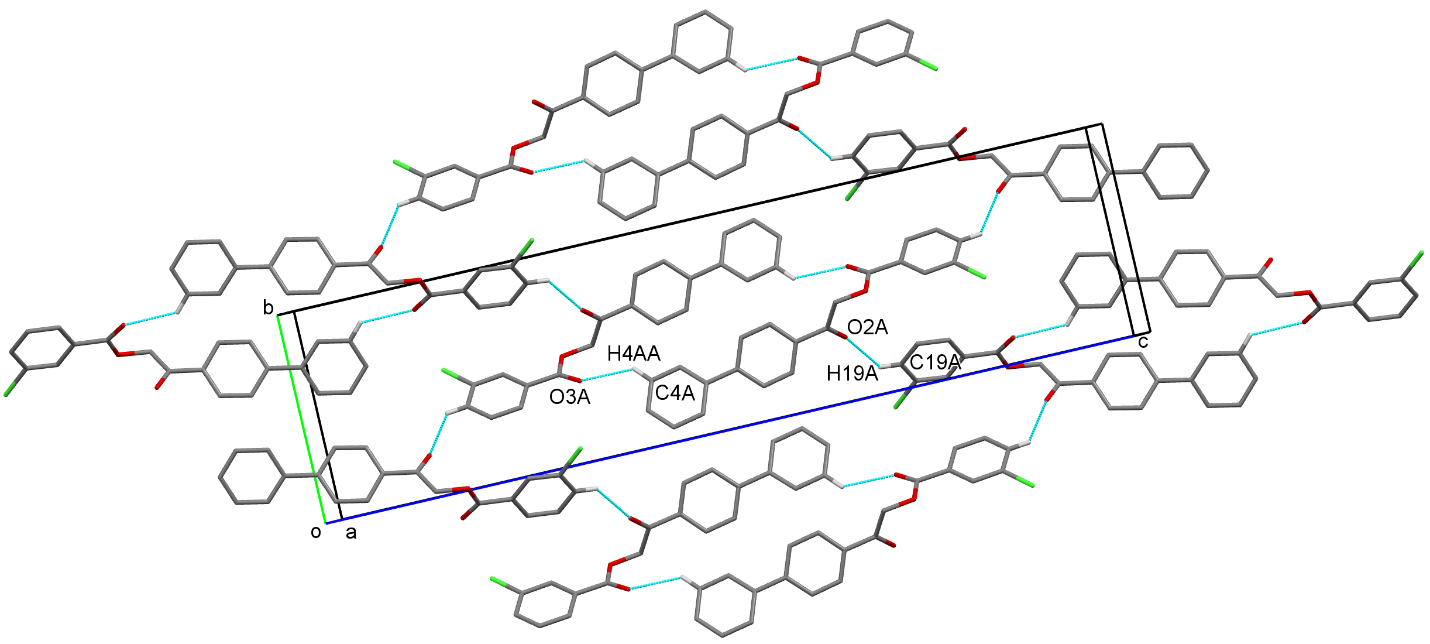


**Figure 2.4**: Partial packing diagram of **2c** showed the 2D sheet along [-102] direction.

In the crystal packing of **2d**, molecules are linked into a centrosymmetric dimer via a weak C17—H17*A*···O3 hydrogen bond and formed a $R_{2}^{2}$(10) ring motive. While atom O3 served as a bidentate acceptor to link those dimers into a 2D plate parallel to *a,b*-plane through C11—H11*A*···O3 hydrogen bond (Figure 2.5). Molecules in 2d were further stabilized by C12—H12*A*···*Cg*1, C—H…π interaction [*Cg*1 is the centroid of the chlorobenzene ring (C16-C21)]. While in the crystal packing of **2e**, molecules also linked into a centrosymmetric dimer vial C18—H18*A*···O2 hydrogen bond (Figure 2.6a). Those dimer were linked into a 3D network *via* a weak C9—H9*A*···Cl2 hydrogen bond (Figure 2.6b). In this crystal packing, weak C—H···π interaction [C21—H21*A*···*Cg*2, hydrogen and acceptor’s distance (H…A distance) = 3.00Å, *Cg*2 is the centroid of benzene ring (C1-C6)] were also observed.


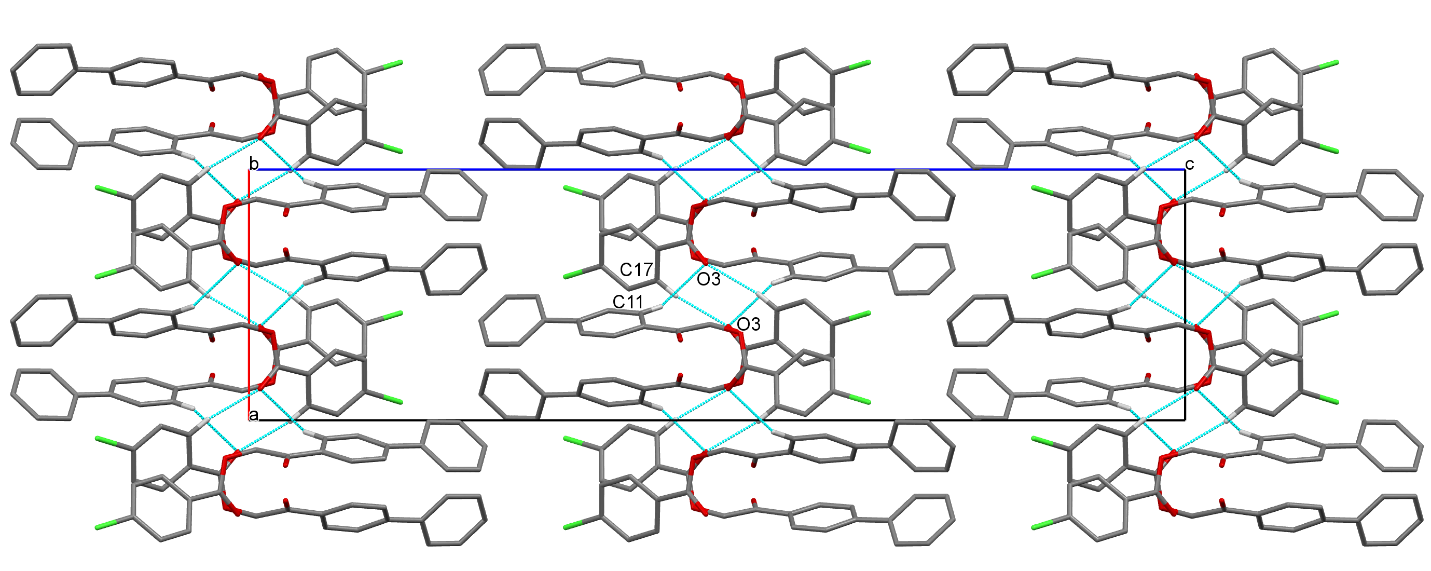


**Figure 2.5**: Packing diagram of **2b** viewed along the c-axis showing 2D plate parallel to *a,b*-plane.

**
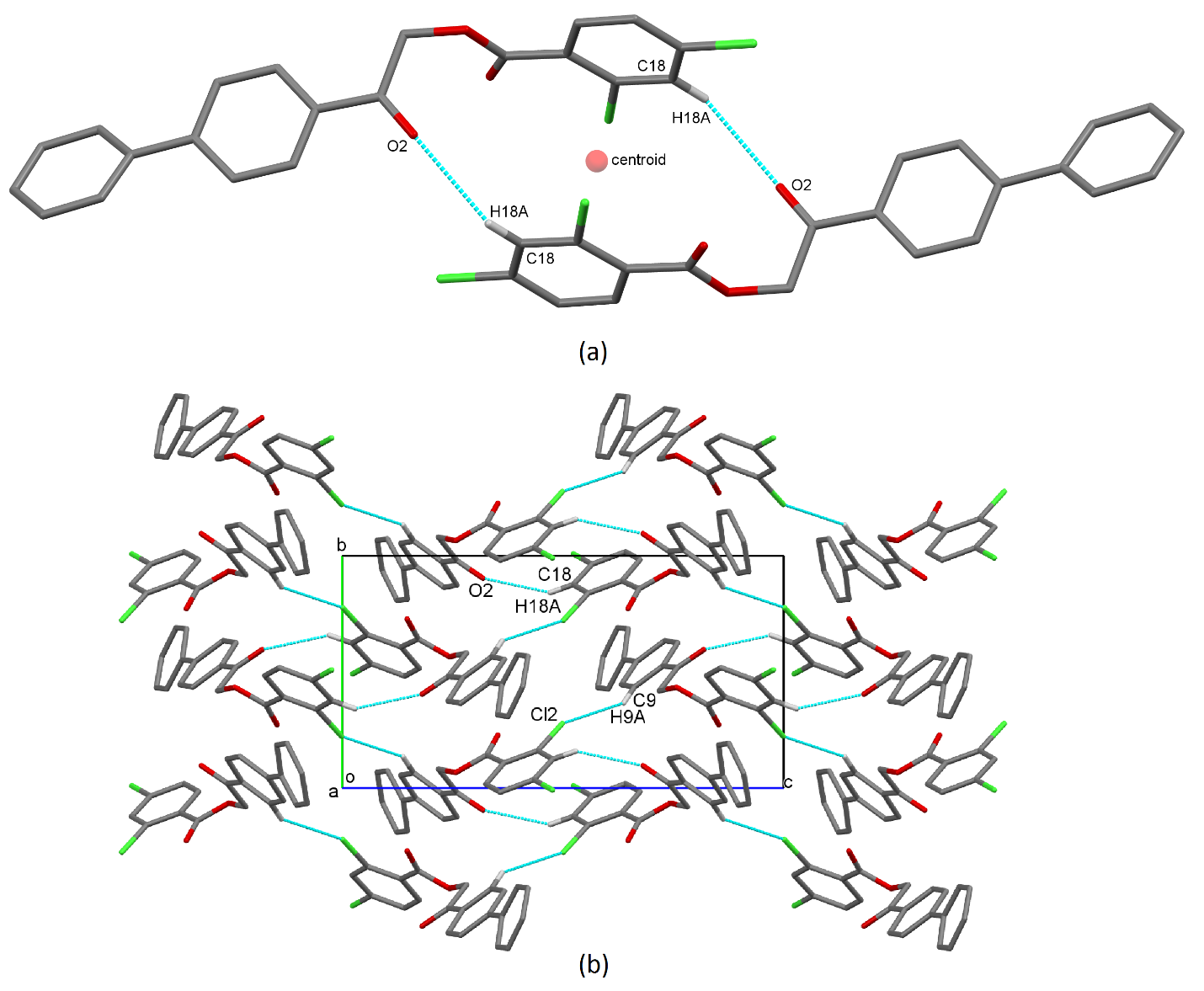
**

**Figure 2.6:(a)** Centrosymmetric dimer of **2e**.**(b)** Packing diagram of **2e** viewed along *a*-axis. Hydrogen atoms that are not involved in hydrogen bonds were omitted for clarity.

Compound **2g** is observed to possess no significant hydrogen bond. It is stabilized by the formation of C17—H17*A*···O1 intra-hydrogen bond and a short contact between H19*A*···O2 which linked those molecules into chain along *b*-axis. As for the crystal packing of **2i**, molecules of **2i** are linked an inversion related dimers via C12—H12*A*···O4 hydrogen bond. Those dimers are linked into a 3D network through C18—H18*A*···O3 and C21—H21*A*···O2 hydrogen bonds (Figure 2.7a). This crystal structure was further stabilize by weak C—H···π interactions involving C1—H1*A*···*Cg*2, C14—H14*B*···*Cg*3 and C22—H22*B*···*Cg*3 with H…A distance 2.98 Å, 2.73 Å and 2.98 Å, respectively [*Cg*2 and *Cg*3 are the centroid of benzene ring C1-C6 and C7-C12] (Figure 2.7b).


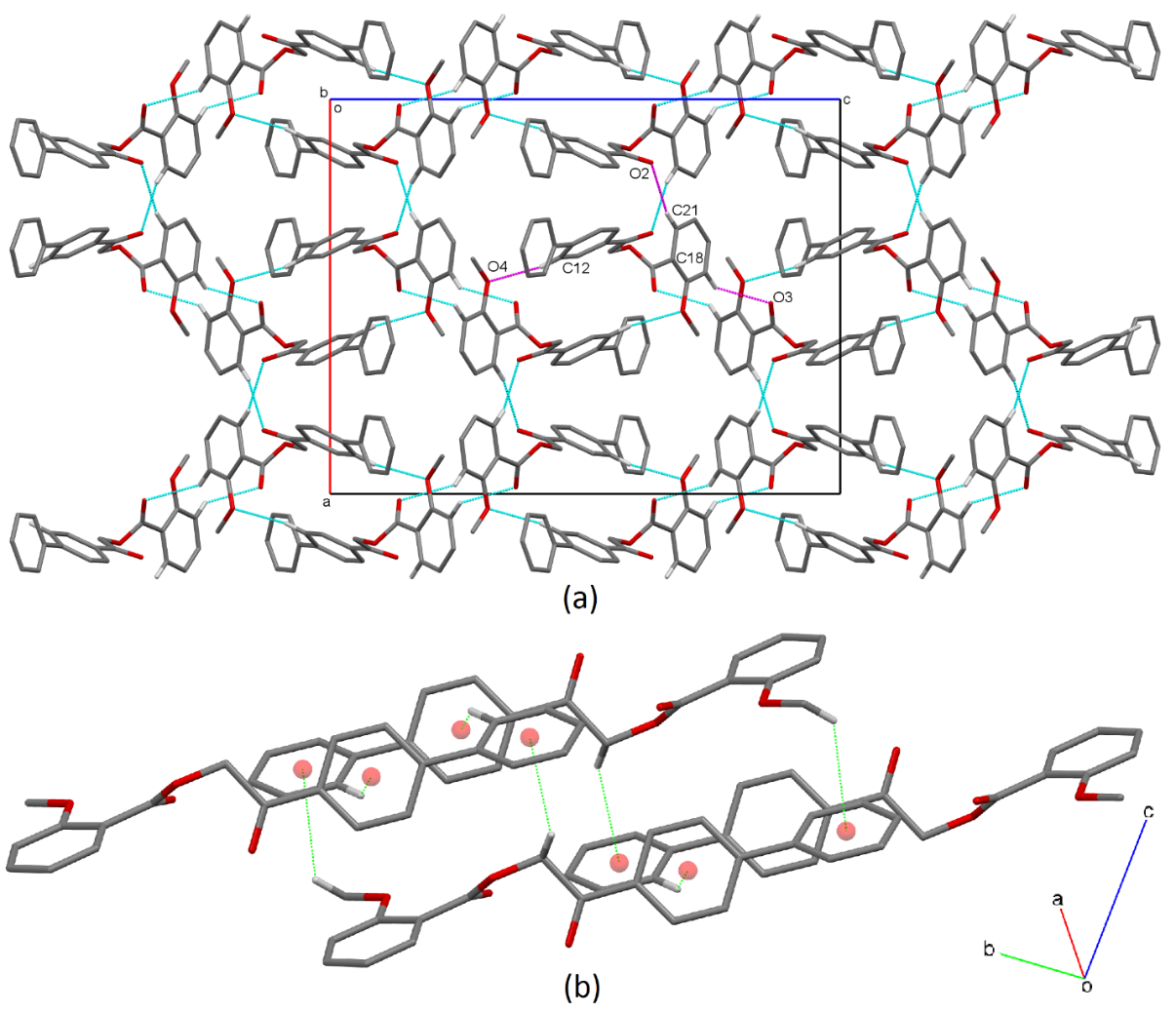


**Figure 2.7**: (a) Packing diagram of **2i** viewed along *b-*axes. (b) C—H…π interaction within crystal packing of **2i.**

In the crystal structure of **2j**, molecules are linked into a zig-zag chain along *a*-axis via a weak C19—H19*A*···O2 hydrogen bond (Figure 2.8a). This chain is further connected in to a 3D network by C14—H14*A*···*Cg*2 and C2—H2*A*···*Cg*3, C—H…π interaction (Figure 2.8b). Although there are no C—H···π or π···π interaction observed in the crystal packing of **2k**, the crystal packing was alleviated by C14—H14*A*···O2 and C20—H20*A*···O2 hydrogen bond and linked those molecules into a column along *b*-axis (Figure 2.9).


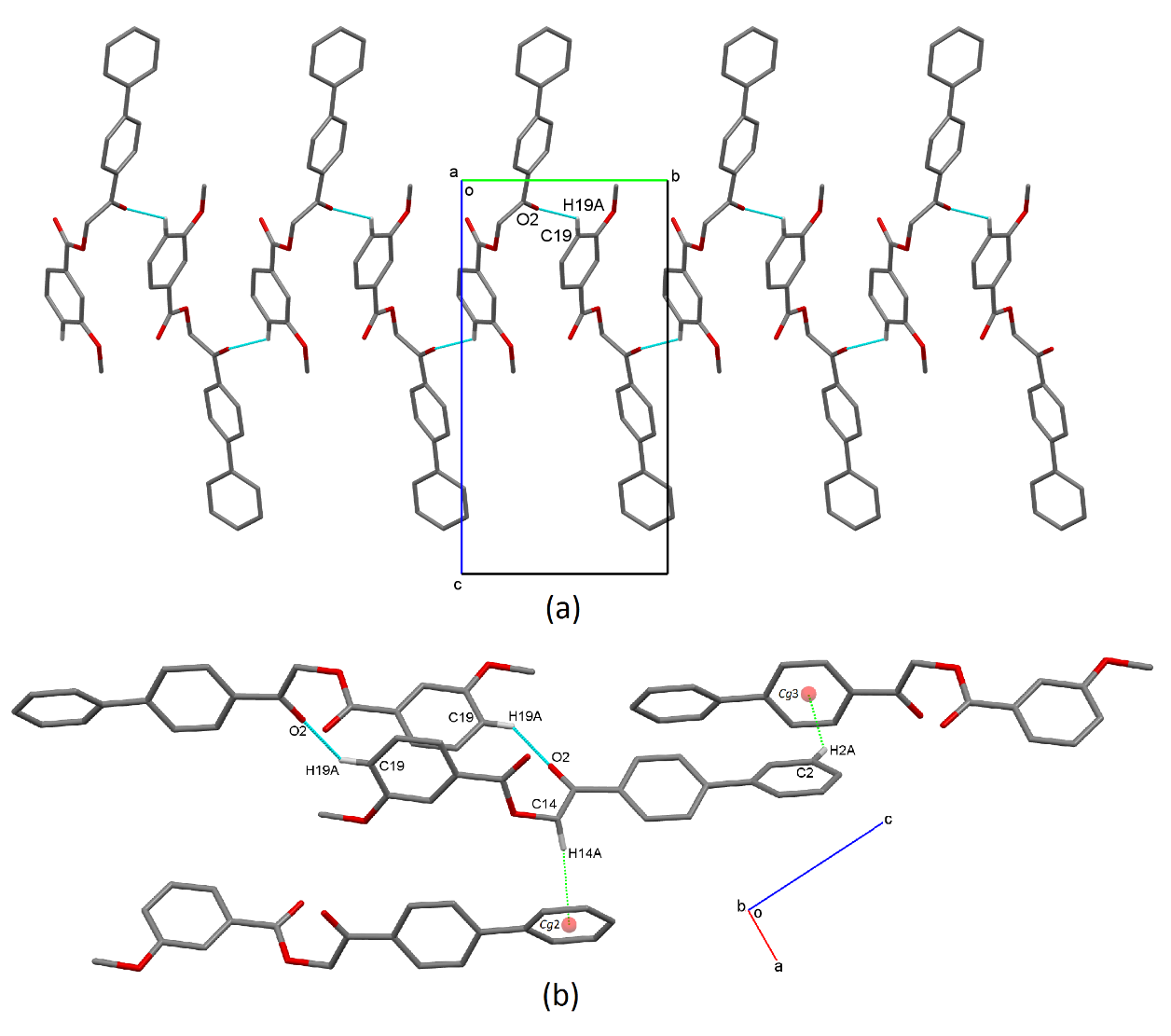


**Figure 2.8:**(a) Partial packing diagrams of **2j** viewed along *a*-axis forming of a zig-zag chain. (b) C—H…π interaction within crystal packing of **2j.**


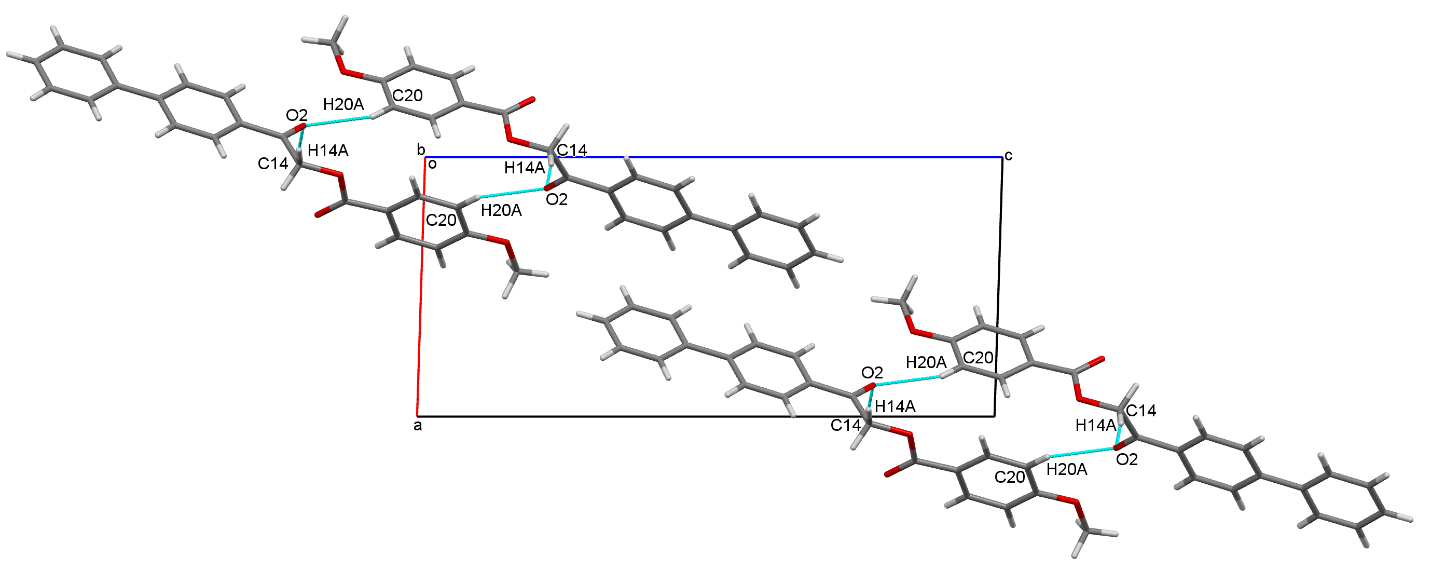


**Figure 2.9**: Partial packing diagram of **2k** forming a column along *b*-axis.

Similar to the crystal packing of **2b**, molecules in the crystal of **2l** were linked in to a chain via C14—H14*B*···O2 hydrogen bond parallel to [130] (Figure 2.10a). However, those chains were further connected by C4—H4*A*···O3 and C19—H19*A*···O4 hydrogen bond into a 3D network (Figure 2.10b). The similar C12—H14···O2 hydrogen bonds were also observed in the crystal packing of **2m** which linking the molecules along *a*-axis. While the asymmetry units in compound **2m** were connected into a centrosymmetric dimer through C17—H17···O5 hydrogen bond. Molecules *A* and molecules *B*dimer are interconnected into a …*AABBAA*… pattern layer along *b*-axis by C20—H20···O2 hydrogen bond (Figure 2.11). C1—H1···*Cg*3, C—H···π interactions were also observed in the crystal packing of **2m**.


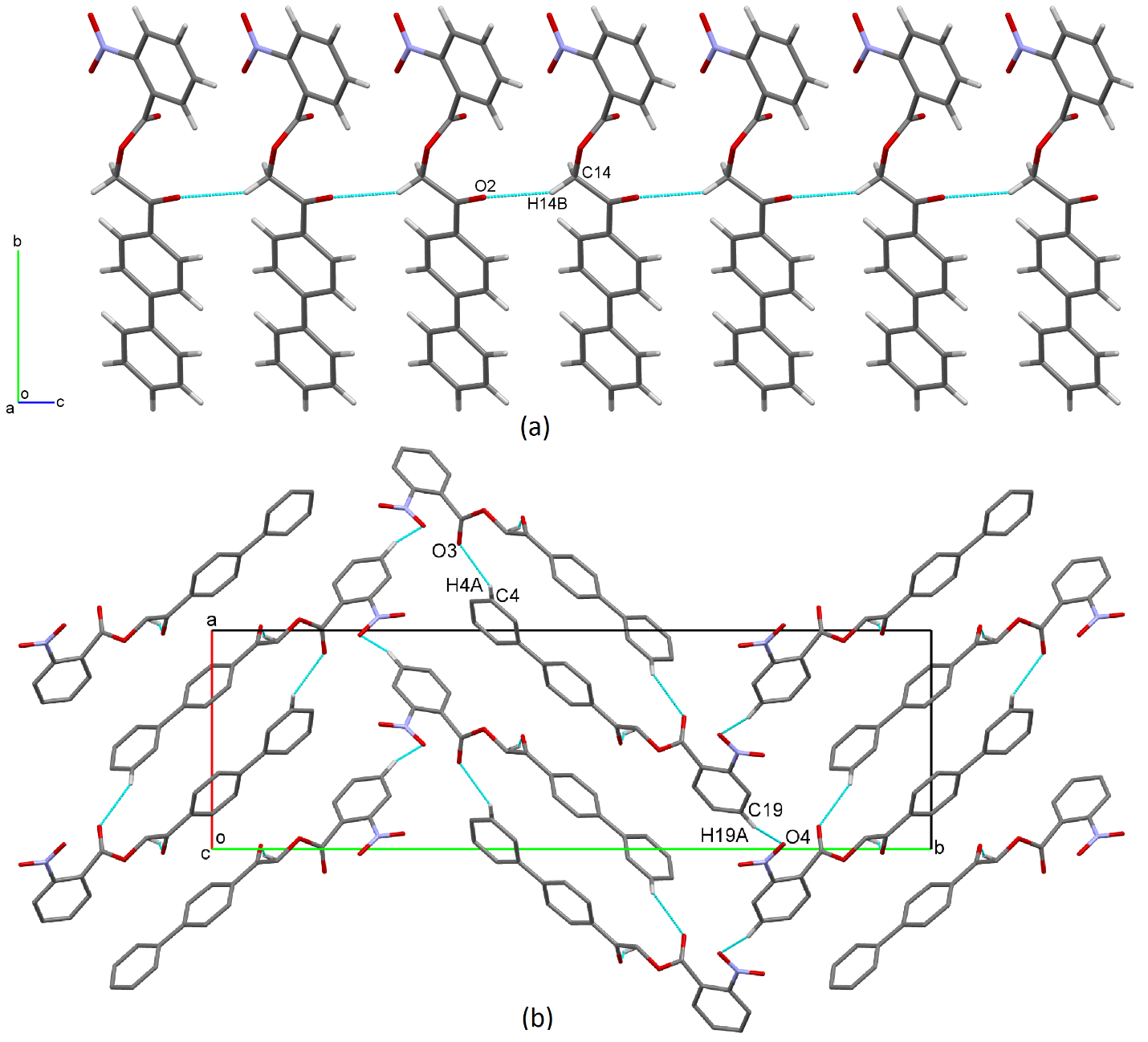


**Figure 2.10**: Partial packing diagram of **2l** viewed along (a) *a-* and (b) *c-*axes.


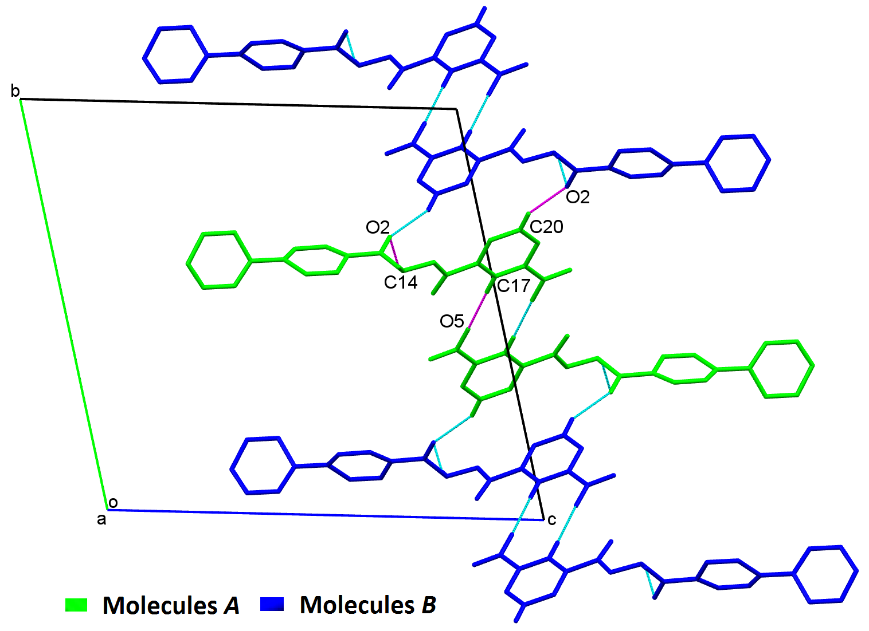


**Figure 2.11**: Partial packing of **2m** forming …*AABBAA*… pattern layer viewed along *a*-axis.

In the crystal of **2n**, molecules are linked into dimers *via* C17—H17*A*···O3 and C20—H20*A*···O5 hydrogen bond forming a $R_{2}^{2}$(10) ring motive’s chains parallel to [-1,2,0] direction. Those chains were further stabilized by C8—H8*A*···*Cg*3, C12—H12*A*···*Cg*2 and C14—H14*B*···*Cg*3, C—H···π interaction (Figure 2.12).


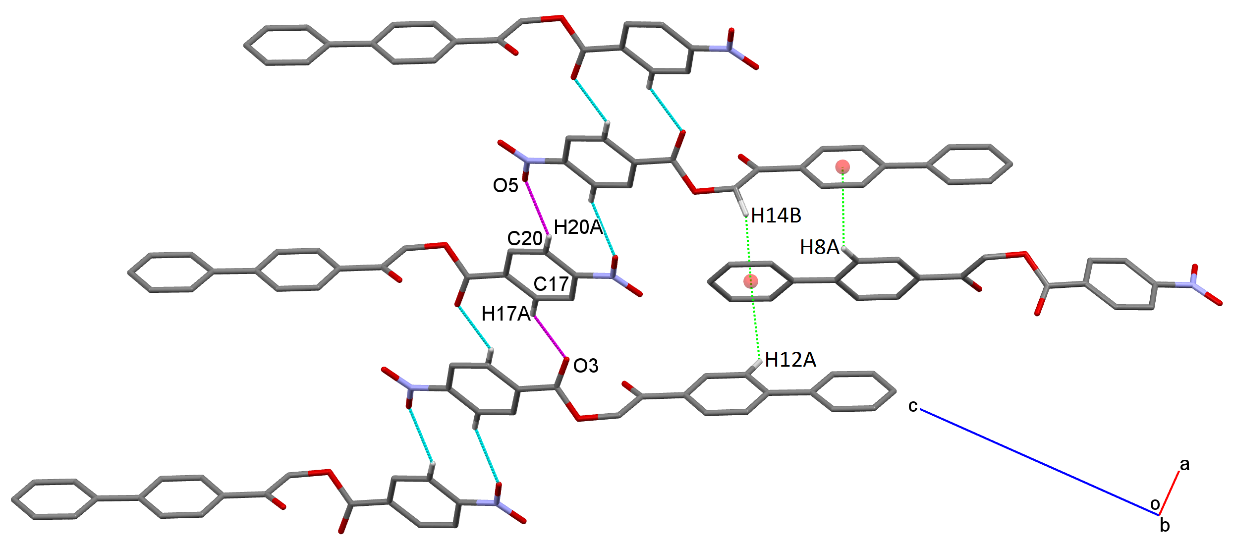


**Figure 2.12**: Partial packing diagram of **2n** viewed along *b-*axes.

In both crystal of **2o** and **2p**, molecules are linked into an inversion related dimer by N1—H1N1···O hydrogen bond forming a $R_{2}^{2}$(12) and $R_{2}^{2}$(14) ring motives respectively (Figure 2.13a). Meanwhile, in **2o** molecules are connected into a herring bone chain by N1—H2N1···O2 hydrogen bond along *a*-axis (Figure 2.13b). While, there are another dimer appear in the crystal packing of **2p***via* N1—H2N1···O2 hydrogen bond and those dimer were linked into a column parallel to *a*-axis by C14—H14*B*···O3 hydrogen bond. Those column were interconnected by C1—H1*A*···*Cg*1, C—H···π interaction (Figure 2.14) [hydrogen and acceptor’s distance (H…A distance) = 2.92 Å]. Similar with other para-substituted compound (**2k** and **2n**), molecules of compound **2q** (4-aminobenzene substituent) were linked into infinite chain *via* C14—H14A···O2 hydrogen bond. This chains are further connected into 3D network by N1—H1N1···O3, N1—H2N1···N1 and C4—H4*A*···O2 hydrogen bonds (Figure 2.15).


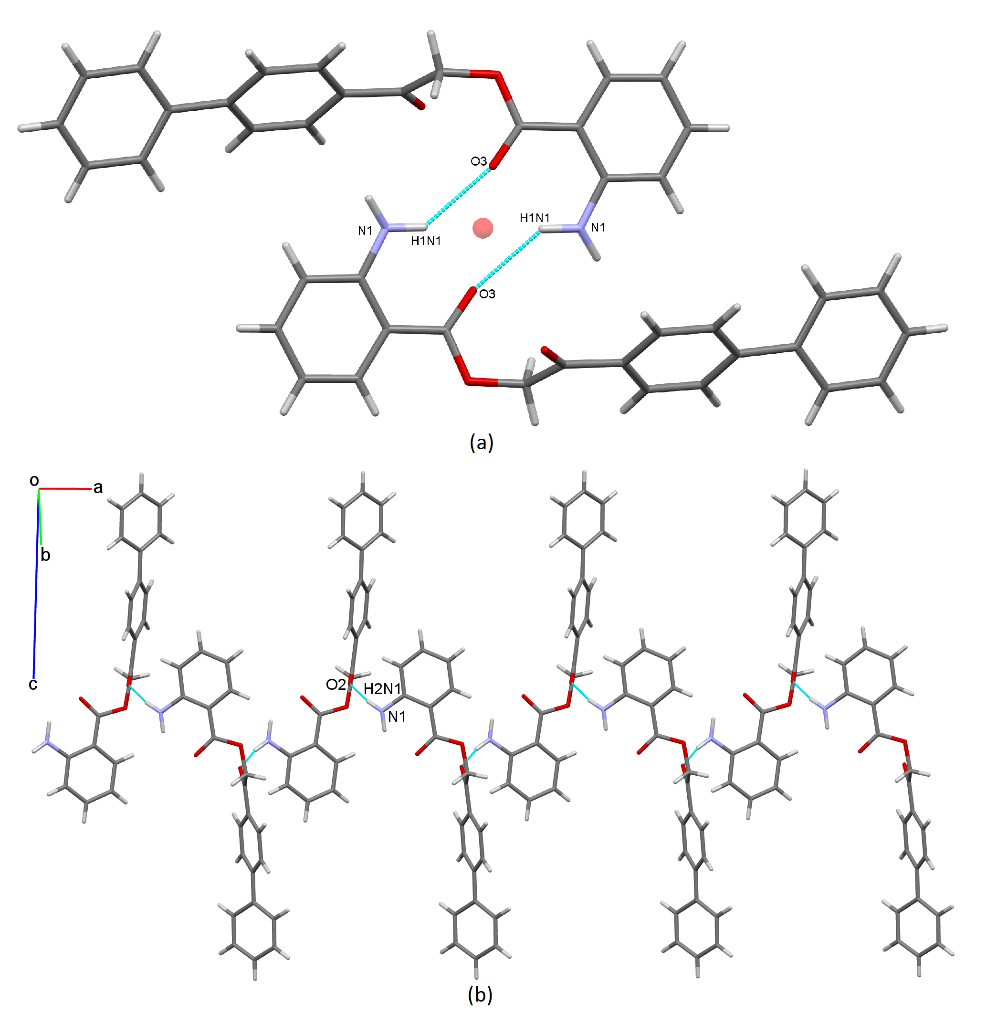


**Figure 2.13:(a)** Centrosymmetric dimer of **2o**.**(b)** Partial packing diagram of **2o** viewed along *b*-axis forming of a herring bone chain.


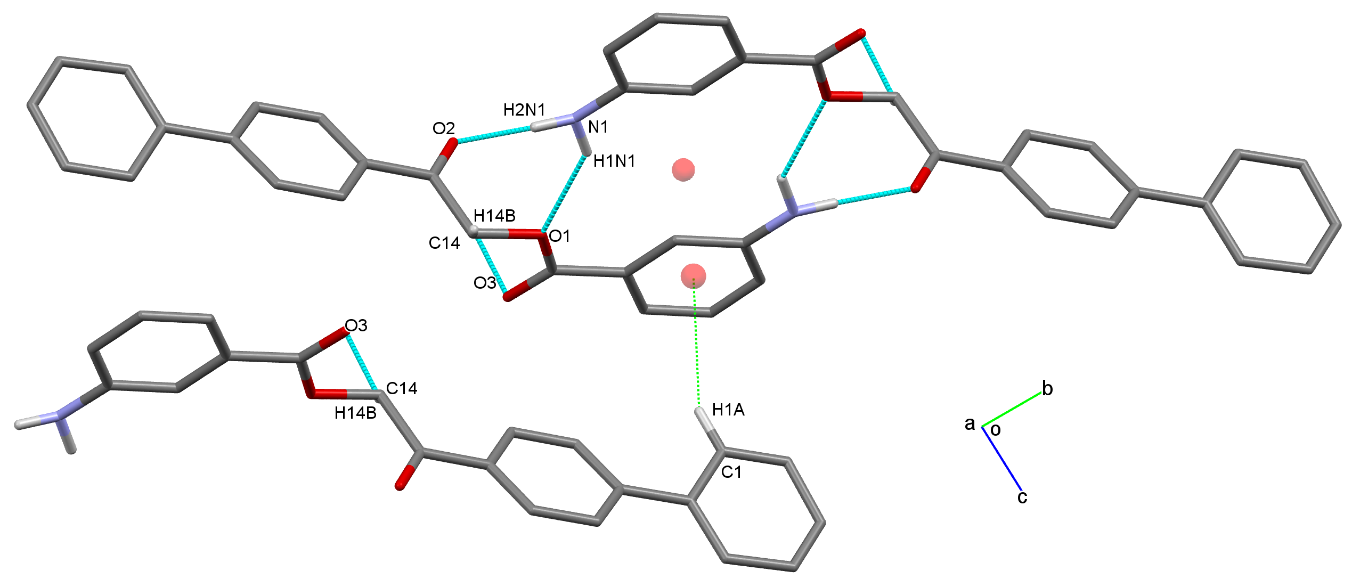


**Figure 2.14**: Partial packing diagram of **2p** which forms infinites linear column along *a*-axis.


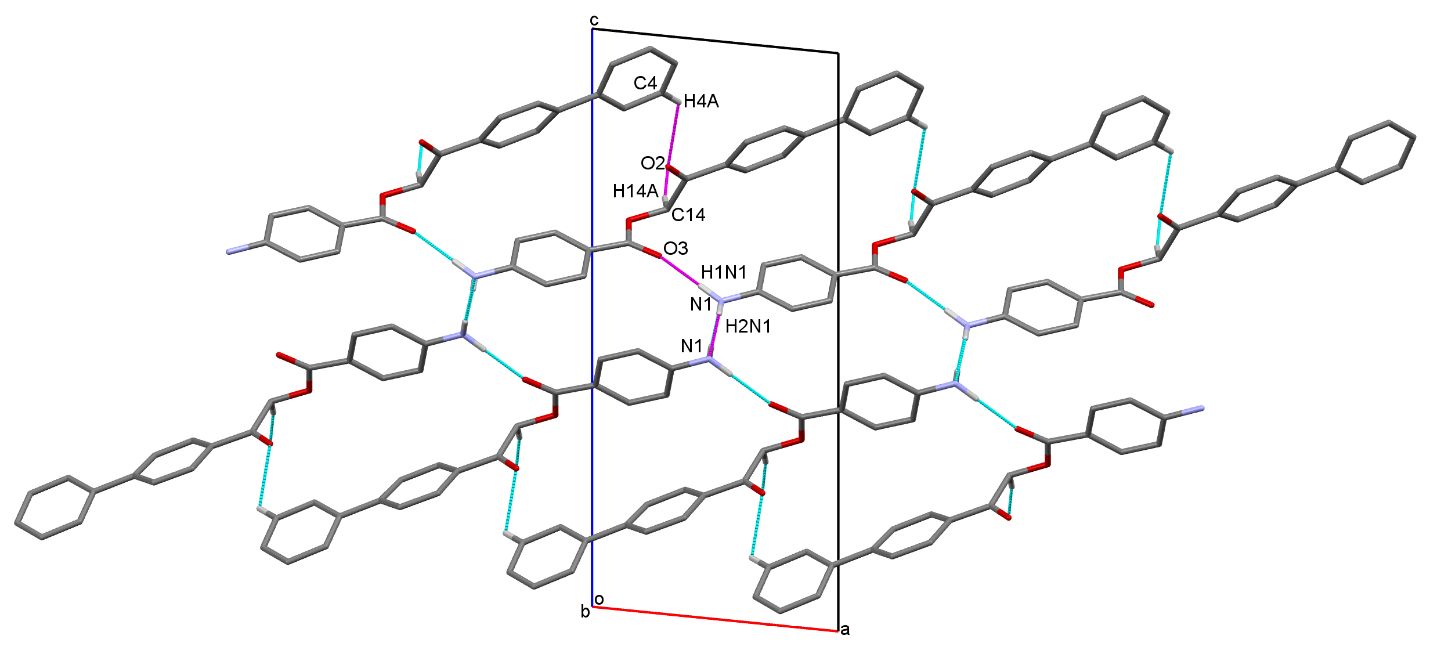


**Figure 2.15**: Packing diagram of **2q** viewed along *a*-axis.

Molecule *A* and *B* in the crystal packing of **2r** share the same types of hydrogen bonds. Both molecules are linked into centrosymmetric dimers by C19—H10···O2 hydrogen bond and interconnected into a …*AABBAA*… pattern plane through C14—H14···O2 and C19—H19···O2 hydrogen bond (Figure 2.16). Meanwhile, two asymmetric unit of **2s** possess different hydrogen bonds. In the crystal of **2s**, molecule *A* is connected with two adjacent molecule *B* by C14*A*—H14*A*···O2*B* and C14*A*—H14*B*···O3*B* hydrogen bond. Whereas, molecule *B* is connected to two molecule *A* through C8*B*—H8*BA*···O3*A* and C14*B*—H14*D*···O2*A* hydrogen bond. However, those hydrogen bond also linked the molecule into a …*AABBAA*… pattern plane parallel to [001] direction. Along the plane, molecule *A* is stacked together by C2*A*—H2*AA*···*Cg*1 and C17*A*—H17*A*···*Cg*3, C—H···π interaction(Figure 2.17). All intermolecular interactions are summarized in section 2.2.


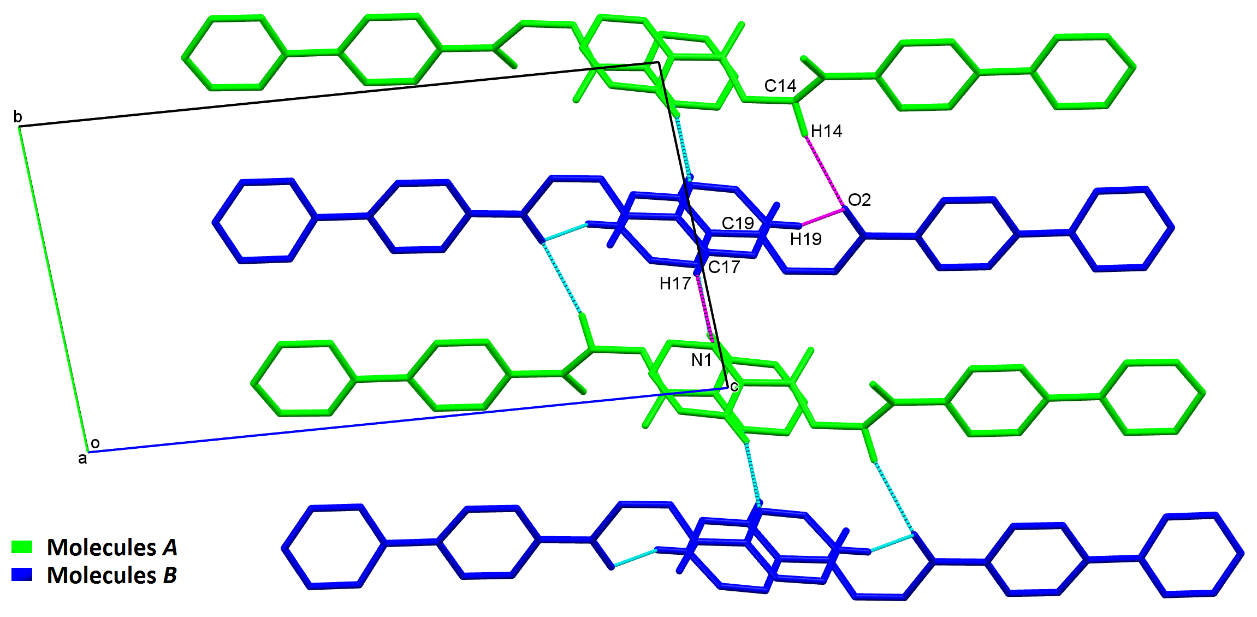


**Figure 2.16**: Partial packing diagram of **2r** viewed along *a*-axis.


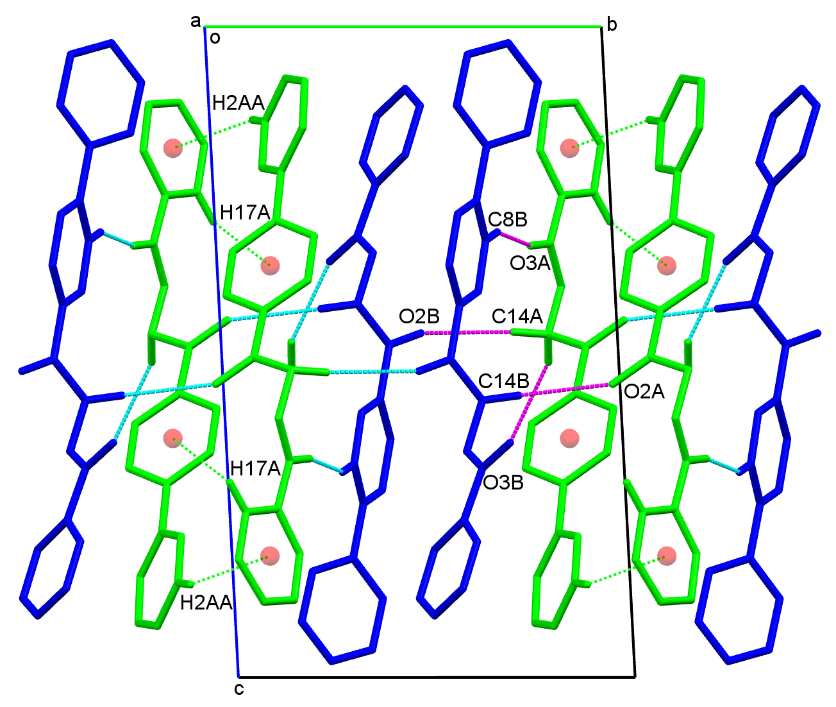


**Figure 2.17**: Partial packing diagram of **2s** viewed along *a*-axis.

## 2.5 Hydrogen-bond geometry

Table 2.4: Hydrogen bond geometries for **2**(**b**-**e**,**g**,**i**-**s**).

| D—H···*A* | D—H (Å) | H···*A* (Å) | D···*A* (Å) | D—H···*A* angles (˚) | Symmetry code |
| --- | --- | --- | --- | --- | --- |
| **2b** |  |  |  |  |  |
| C14—H14*B*···O2 | 0.97 | 2.51 | 3.415 | 155 | 3/2-*x*,1/2+*y*,*z* |
| *Cg*1···*Cg*1 |  |  | 3.564 |  | 1-*x*,-*y*,1-*z* |
| **2c** |  |  |  |  |  |
| C4*B*—H4*BA*···O3*A* | 0.93 | 2.48 | 3.210 | 135 | 1-*x*,1-*y*,1-*z* |
| C19*A*—H19*A*···O2*A* | 0.93 | 2.59 | 3.242 | 127 | 2-*x*,1/2+*y*,3/2-*z* |
| **2d** |  |  |  |  |  |
| C11—H11*A*···O3 | 0.95 | 2.54 | 3.424 | 154 | -1/2+*x*,3/2-*y*,1-*z* |
| C17—H17*A*···O3 | 0.95 | 2.35 | 3.290 | 170 | 2-*x*,1-*y*,1-*z* |
| C12—H12*A*···*Cg*3 | 0.95 | 2.74 | 3.595 | 150 | -1/2+*x*,3/2-*y*,1-*z* |
| **2e** |  |  |  |  |  |
| C9—H9*A*···Cl2 | 0.93 | 2.79 | 3.611 | 148 | *x*,1/2-*y*,-1/2+*z* |
| C18—H18*A*···O2 | 0.93 | 2.60 | 3.424 | 149 | -*x*,-*y*,1-*z* |
| C21—H21*A*···*Cg*1 | 0.93 | 3.00 | 3.729 | 137 | -*x*,-*y*,1-*z* |
| **2g** |  |  |  |  |  |
| no H-bond observed |  |  |  |  |  |
| **2i** |  |  |  |  |  |
| C12—H12*A*···O4 | 0.95 | 2.53 | 3.445 | 163 | -*x*,2-*y*,-*z* |
| C18—H18*A*···O3 | 0.95 | 2.56 | 3.279 | 132 | -*x*,-1/2+*y*,1/2-*z* |
| C21—H21*A*···O2 | 0.95 | 2.55 | 3.149 | 122 | 1/2-*x*,-1/2+*y*,*z* |
| C11—H1*A*···*Cg*2 | 0.95 | 2.98 | 3.711 | 134 | -*x*,3-*y*,-*z* |
| C14—H14*B*···*Cg*1 | 0.99 | 2.73 | 3.506 | 136 | *x*,-1+*y*,*z* |
| C22—H22*B*···*Cg*1 | 0.98 | 2.98 | 3.655 | 127 | -*x*,2-*y*,-*z* |
| **2j** |  |  |  |  |  |
| C19—H19*A*···O2 | 0.95 | 2.53 | 3.185 | 126 | 2-*x*,-1/2+*y*,1/2-*z* |
| C2—H2*A*···*Cg*2 | 0.95 | 2.83 | 3.639 | 144 | 1-*x*,1/2+*y*,-1/2-*z* |
| C14—H14*A*···*Cg*1 | 0.99 | 2.67 | 3.506 | 142 | *x*,1/2-*y*,1/2+*z* |
| **2k** |  |  |  |  |  |
| C14—H14*A*···O2 | 0.97 | 2.56 | 3.514 | 169 | *x*,-1+*y*,*z* |
| C20—H20*A*···O2 | 0.93 | 2.53 | 3.420 | 159 | -*x*,-1/2+*y*,-*z* |
| **2l** |  |  |  |  |  |
| C4—H4*A*···O3 | 0.95 | 2.54 | 3.344 | 143 | 2-*x*,1-*y*,-1/2+*z* |
| C14—H14*B*···O2 | 0.99 | 2.45 | 3.348 | 150 | *x*,*y*,-1+*z* |
| C19—H19*A*···O4 | 0.95 | 2.58 | 3.376 | 142 | 1/2+*x*,3/2-*y*,1+*z* |
| **2n** |  |  |  |  |  |
| C14*A*—H14*A*···O2*A* | 0.99 | 2.59 | 3.537 | 160 | -1+*x*,*y*,*z* |
| C14*B*—H14*D*···O2*B* | 0.99 | 2.59 | 3.534 | 160 | 1+*x*,*y*,*z* |
| C17*A*—H17*A*···O5*A* | 0.95 | 2.46 | 3.406 | 173 | 2-*x*,1-*y*,2-*z* |
| C17*B*—H17*B*···O5*B* | 0.95 | 2.46 | 3.402 | 173 | 1-*x*,-*y*,2-*z* |
| C20*A*—H20*A*···O2*B* | 0.95 | 2.39 | 3.279 | 156 | 1-*x*,1-*y*,2-*z* |
| C20*B*—H20*B*···O2*A* | 0.95 | 2.39 | 3.280 | 156 | 2-*x*,1-*y*,2-*z* |
| C1*A*—H1*AA*···*Cg*4 | 0.95 | 2.90 | 3.777 | 154 | -*x*,1-*y*,1-*z* |
| C1*B*—H1*BA*···*Cg*9 | 0.95 | 2.90 | 3.774 | 154 | 2-*x*,-*y*,1-*z* |
| **2m** |  |  |  |  |  |
| C17—H17*A*···O3 | 0.93 | 2.44 | 3.229 | 143 | 2-*x*,2-*y*,-*z* |
| C20—H20*A*···O5 | 0.93 | 2.53 | 3.345 | 147 | 3-*x*,-*y*,-*z* |
| C8—H8*A*···*Cg*2 | 0.93 | 2.95 | 3.686 | 134 | 2-*x*,1/2+*y*,1/2-*z* |
| C12—H12*A*···*Cg*1 | 0.93 | 2.95 | 3.633 | 131 | 1-*x*,-1/2+*y*,1/2-*z* |
| C14—H14*B*···*Cg*1 | 0.97 | 2.81 | 3.692 | 151 | 2-*x*,1/2+*y*,1/2-*z* |
|  |  |  |  |  |  |
|  |  |  |  |  |  |
| D—H···*A* | D—H (Å) | H···*A* (Å) | D···*A* (Å) | D—H···*A* angles (˚) | Symmetry code |
| **2o** |  |  |  |  |  |
| N1—H2N1···O2 | 0.91 | 2.25 | 3.059 | 149 | -1/2+x,-1/2-y,1-z |
| N1—H1N1···O3 | 0.93 | 2.24 | 3.008 | 140 | 1-x,-y,1-z |
| **2p** |  |  |  |  |  |
| N1—H1N1···O1 | 0.94 | 2.54 | 3.347 | 144 | 1-x,-y,-z |
| N1—H2N1···O2 | 0.88 | 2.24 | 3.084 | 162 | -x,-y,-z |
| C14—H14*B*···O3 | 0.97 | 2.57 | 3.384 | 142 | 1+x,y,z |
| C1—H1*A*···*Cg*4 | 0.93 | 2.92 | 3.671 | 152 | 1-x,1-y,-z |
| **2q** |  |  |  |  |  |
| N1—H1N1···O3 | 0.90 | 2.10 | 2.979 | 163 | 1+x,y,z |
| N1—H2N1···N1 | 0.90 | 2.34 | 3.241 | 166 | 3-x,-1/2+y,1-z |
| C4—H4*A*···O2 | 0.95 | 2.59 | 3.421 | 126 | -1+x,1+y,z |
| C14—H14*A*···O2 | 0.99 | 2.50 | 3.482 | 172 | x,1+y,z |
| **2r** |  |  |  |  |  |
| C14*B*—H14*A*···O2*A* | 0.97 | 2.59 | 3.455 | 149 | x,y,z |
| C14*A*—H14*C*···O2*B* | 0.97 | 2.36 | 3.324 | 174 | x,1+y,z |
| C17*B*—H17*A*···N1*A* | 0.93 | 2.60 | 3.410 | 146 | 2-x,1-y,2-z |
| C17*A*—H17*B*···N1*B* | 0.93 | 2.49 | 3.305 | 147 | 1-x,-y,2-z |
| C19*A*—H19*B*···O2*A* | 0.93 | 2.50 | 3.284 | 142 | 2-x,1-y,2-z |
| C19*B*—H19*A*···O2*B* | 0.93 | 2.70 | 3.394 | 132 | 2-x,-y,2-z |
| **2s** |  |  |  |  |  |
| C14*A*—H14*A*···O2*B* | 0.97 | 2.49 | 3.454 | 173 | 1+x,y,z |
| C14*A*—H14*B*···O3*B* | 0.97 | 2.42 | 3.275 | 147 | x,1-y,1-z |
| C8*B*—H8*BA*···O3*A* | 0.93 | 2.34 | 3.224 | 158 | 1-x,1-y,1-z |
| C14*B*—H14*D*···O2*A* | 0.97 | 2.53 | 3.503 | 178 | -1+x,1+y,z |
| C2*A*—H2*AA*···*Cg*1 | 0.93 | 2.83 | 3.646 | 147 | 2-x,-y,1-z |
| C17*A*—H17*A*···*Cg*3 | 0.93 | 2.82 | 3.634 | 147 | 1-x,-y,1-z |

*Cg*1 = C16-C17-C18-C19-C20-C21 / C16*A*-C17*A*-C18*A*-C19*A*-C20*A*-C21*A* ring.

*Cg*2 = C1-C2-C3-C4-C5-C6 ring.

*Cg*3 = C7-C8-C9-C10-C11-C12 / C7*A*-C8*A*-C9*A*-C10*A*-C11*A*-C12*A* ring.

# 3.0 Anti-tyrosinase Assay

## 3.1 Percentage of inhibition

Table 3.1: Percentage of tyrosinase inhibition for **2**(**i, o**, **p**, **r** and **s**).

|  |  | **50 ug/mL** | | **100 ug/mL** | | **250 ug/mL** | |
| --- | --- | --- | --- | --- | --- | --- | --- |
| **Sample** | | MEAN | RSD % | MEAN | RSD % | MEAN | RSD % |
| Kojic Acid | | 48.91 | 1.1390485 | 53.26 | 7.882125 | 57.22 | 5.35663665 |
| 2i | | 35.28 | 3.4058671 | 51.66 | 8.3131070 | 48.12 | 2.7537485 |
| 2o | | 37.97 | 6.5089155 | 45.70 | 8.3086178 | 48.13 | 5.5099479 |
| 2p | | 40.51 | 4.7332969 | 51.10 | 0.53751313 | 57.33 | 2.81214628 |
| 2r | | 42.97 | 4.80954852 | 52.20 | 5.6759858 | 58.90 | 3.452536 |
| 2s | | 46.24 | 5.5034545 | 50.91 | 1.23247923 | 60.34 | 1.65025267 |


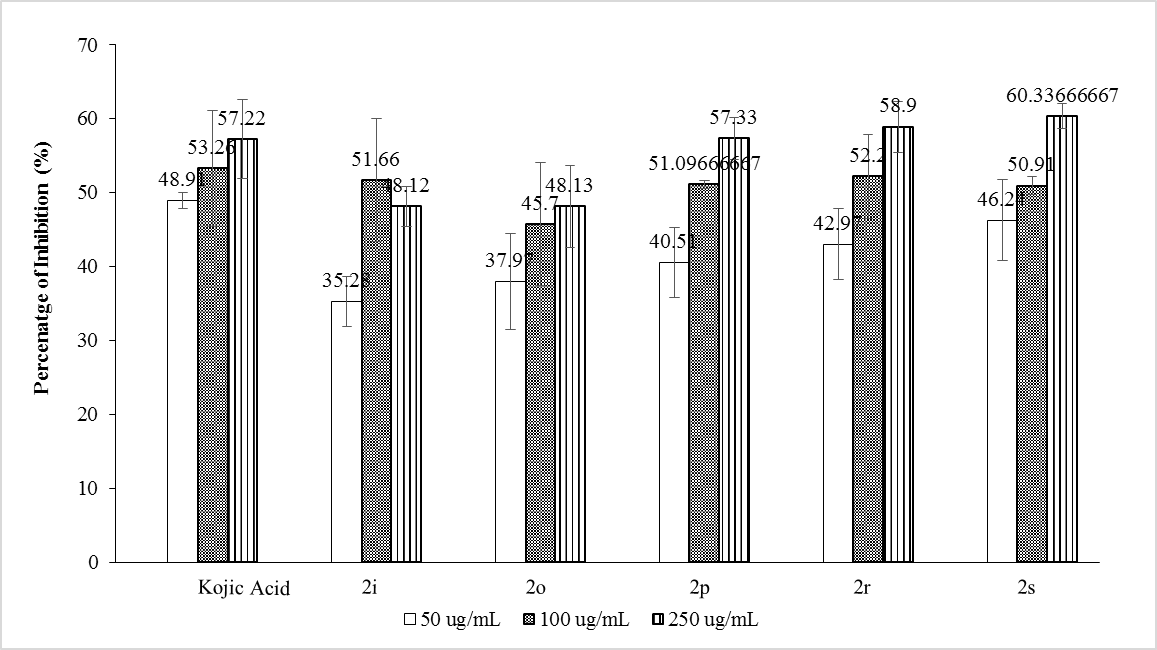


**Figure 3.1**: Inhibition of tyrosinase enzyme. The data represent the percentage of tyrosinse inhibition (mean±SD), n=3, p<0.05,compare with standard compound, kojic acid
